# Dissecting Domain-Specific Evolutionary Pressure Profiles of Transient Receptor Potential Vanilloid Subfamily Members 1 to 4

**DOI:** 10.1371/journal.pone.0110715

**Published:** 2014-10-21

**Authors:** Pau Doñate-Macián, Alex Perálvarez-Marín

**Affiliations:** Unitat de Biofísica, Centre d’Estudis en Biofísica, Departament de Bioquímica i de Biologia Molecular, Universitat Autònoma de Barcelona, Bellaterra, Spain; The City University of New York-Graduate Center, United States of America

## Abstract

The transient receptor potential vanilloid family includes four ion channels–TRPV1, TRPV2, TRPV3 and TRPV4–that are represented within the vertebrate subphylum and involved in several sensory and physiological processes. These channels are related to adaptation to the environment, and probably under strong evolutionary pressure. Using multiple sequence alignments as source for evolutionary, bioinformatics and statistical analysis, we have analyzed the evolutionary profiles for TRPV1, TRPV2, TRPV3 and TRPV4. The evolutionary pressure exerted over vertebrate TRPV2 sequences compared to the other channels argues for a positive selection profile for TRPV2 compared to TRPV1, TRPV3 and TRPV4. We have analyzed the selective pressure on specific protein domains, observing a common selective pressure trend for the common TRPV scaffold, consisting of the ankyrin repeat domain, the membrane proximal domain, the transmembrane domain, and the TRP domain. Through a more detailed analysis we have identified evolutionary constraints involved in the subunit contact at the transmembrane domain level. Performing evolutionary comparison, we have translated specific channel structural information such as the transmembrane topology, and the interaction between the membrane proximal domain and the TRP box. We have also identified potential common regulatory domains among all TRPV1-4 members, such as protein-protein, lipid-protein and vesicle trafficking domains.

## Introduction

TRP channel superfamily consists of a set polymodal non-selective oligomeric membrane cationic channels, with large cytoplasmic regulatory domains [1], [2]. These channels are predicted to share a common tetrameric membrane topology around the formation of a pore in the membrane to allow the flux of cations, but there are several differential regulatory domains that allow/block the cation flux through the membrane [2]. These domains are very specialized, and follow an evolutionary pattern that has been reflected in the subfamily classification of the large TRP superfamily. The vanilloid subfamily (TRPV) in vertebrates consists of at least six members (TRPV1-6) [3]. From an evolutionary perspective, there are two subgroups within this subfamily, first, TRPV1-4 which are non-selective cation channels, and second, TRPV5 and TRPV6, which are calcium selective ion channels. Another classification identifies the TRPV1-4 subgroup as thermosensors in mammals: TRPV1 and TRPV2 act as noxious heat sensors (T>43°C), and TRPV3 and TRPV4 as physiological temperature sensors.

Evolutionary studies on TRPV channels, have attempted to gain information on the evolution profile of the family [4], [5], or on the identification of specific domains in TRPV1 [6]. Understanding how evolution drives specialization of functional and structural domains has been and is a bioinformatics challenge [7], [8], especially when the study focus is multi-domain oligomeric membrane proteins, such as TRP channels. When considering membrane proteins, one should take into account protein-protein and lipid-protein contacts, internal transmembrane polar clusters, etc. Evolution information derived from the primary sequence may provide important hints about how a membrane protein is integrated in its environment. Biologically significant positions in a protein can be inferred by identifying directional selection in comparison to neutral selection. Neutral selection indicates low evolutionary pressure and directional selection indicates high evolutionary pressure that can follow two ways: positive selection *versus* negative (purifying) selection events. Purifying selection acts towards function conservation, whereas positive selection argues for environment adaptation or species/tissue dependent function variability, thus selective pressure defines the evolutionary history of a protein. Some studies have used evolutionary constrains to provide general information, such as domain organization and spatial interaction, and even mapping the evolutionary constrains for automated modeling of membrane proteins [9]–[12]. However, to understand specific issues, such as topology, selective pressure on biologically significant residues, or domain conservation, a detailed study and characterization of the system of interest is required.

In this study, we provide a comprehensive depiction of the evolutionary profile of the non-selective cation channels from the TRPV subfamily, i.e. TRPV1, TRPV2, TRPV3, and TRPV4 channels. We analyze the global evolutionary selective pressure for TRPV1-4 channels and the selective pressure exerted on specific domains as a candidate force driving function differentiation.

## Results

### Identifying evolutionary traits among TRPV1-4 channels

To dissect the common evolutionary features among TRPV1-4 sequences, we carried out a computational phylogenetic analysis. First, we retrieved the different sequences for TRPVs available in public databases. We also inspected specific genomes to get the complete protein sequence from some fragment TRPV sequences available in the UNIPROT database [13]. All the protein sequences used in this study are available as Dataset S1. Specifically, from the UNIPROT and NCBI databases (2011) we could curate the TRPV1 full sequence for: *Equus caballus, Salmo salar, Monodelphis domestica, Sarcophilus harrisii* and *Putorius furo*; and the TRPV2 full sequence for: *Tursiops truncatus*, *Dipodormys ordii* and *Gasterosteus aculeatus*.

**Figure 1:**
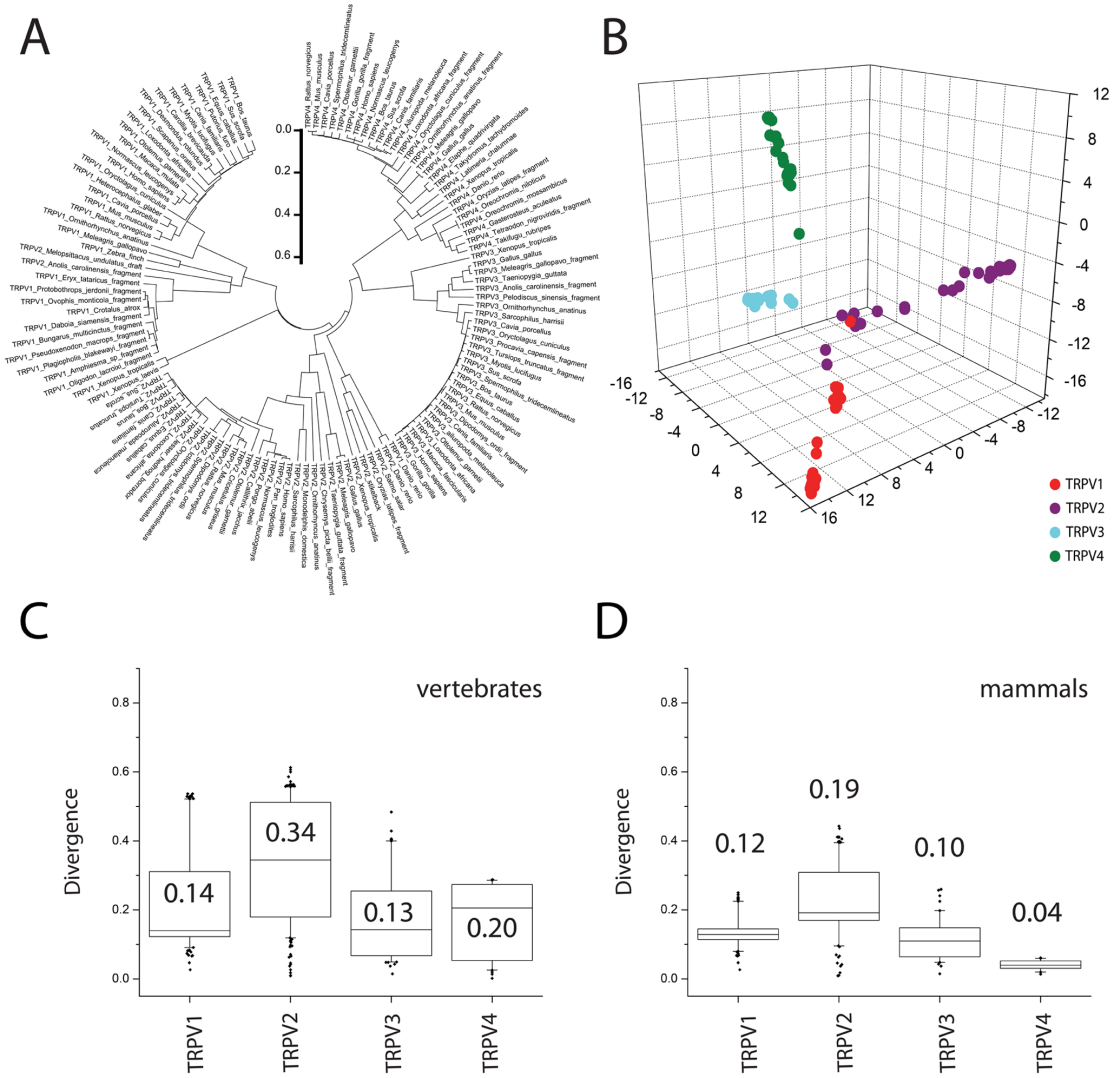
TRPVs phylogeny. **A.** Radial phylogenetic tree for TRPVs. The scale bar indicates evolutionary time in arbitrary units. **B.** Clustering by PCA analysis for TRPVs. Attending to their evolutive distances TRPV1-4 could be clustered into 4 subgroups corresponding to each of the four channels. **C.** Sequence divergence profile for vertebrate TRPVs. **D.** Sequence divergence profile for mammalian TRPVs. The box plots represent the divergence of sequences distribution for each channel. The median value is indicated for each box plot. Refer to the text for specific statistical comparison of the medians.

With all sequences available, we performed the multiple sequence alignment (MSA) that depicted a phylogenetic tree (Fig. 1A). This tree revealed a clear distribution of the channels within the corresponding evolutionary distribution in four defined groups. To provide a more illustrative depiction (Fig. 1B), we clustered the sequences using principal component analysis in JalView [14]. Some TRPV2 fragment sequences (*Melopsittacus undulatus* and *Anolis carolinensis*) were clustered in the TRPV1 subgroup (Fig. 1A). TRPV3 and TRPV4 clusters were clearly identified (Fig. 1B) and originated from the divergent node that defines the fish TRPV1/2 cluster (Fig. 1A).

### TRPVs evolutionary pressure

Using a subset of TRPV channels sequences we compared the conservation distribution for TRPV1 (25 sequences), TRPV2 (28 sequences), TRPV3 (21 sequences), and TRPV4 (22 sequences). We used the directional selection algorithm (FADE) of the HyPhy package [15], [16] to analyze the MSAs for each channel and detect differences in the substitution rate as a rough indicator of positive selection among TRPVs (Table 1). The substitution rate in sequences for vertebrates was TRPV2>TRPV1>TRPV4>TRPV3, however, TRPV4 has a higher number of sites under positive selection (177, Table 1), i.e. positive selected residues compared to the other TRPVs. In the case of mammalian sequences, the substitution rate was TRPV2>TRPV1>TRPV3>TRPV4, and TRPV2 is the channel with higher number of residues under positive selection, whereas TRPV4 shows a strong purifying selective pressure in mammals. To measure the variability within each channel set of sequences we computed the pairwise distances among all sequences for a specific channel and represented them in box plots for divergence frequency distribution (Fig. 1C). We compared all available sequences for each channel depicting the sequence variability in the vertebrate subset (Fig. 1C). The level of sequence divergence among species was highest for TRPV2 (median at 0.34), followed by TRPV4 (0.20), TRPV1 (0.14), and the least divergent is TRPV3 (0.13). There was a bias on the number of sequences available for TRPV channels in the databases depending on the phylogenetic group, where mammals are the most represented. In Fig. 1D, we show the sequence variability for each channel only considering mammals information. Although at lesser extent, the level of sequence divergence was still highest for TRPV2 (median at 0.19), followed by TRPV1 (0.12), TRPV3 (0.11) and TRPV4 (0.04). Results for sequence divergence in Fig. 1C and Fig. 1D agree reasonably well with the directional selection analysis of the MSAs carried out in HyPhy (Table 1).

**Table 1:** Evolutionary analysis of TRPV1-4 channels for directional positive selection based on multiple sequence alignments.

	Vertebrates			
	TRPV1	TRPV2	TRPV3	TRPV4
substitution/site	4.49	8.57	2.43	3.21
residues in alignment	964	915	829	906
sequences	25	28	21	22
sites under positive selection*	100	158	69	177
	**Mammals**			
	**TRPV1**	**TRPV2**	**TRPV3**	**TRPV4**
substitution/site	1.88	3.66	1.19	0.33
residues in alignment	875	821	811	872
sequences	20	22	18	11
sites under positive selection*	72	106	37	13

*considering as significant the top 5% sites in the FADE analysis (0.95 posterior filter).

To determine whether divergence variance among TRPVs could fit into any of these directional evolutionary pressure hypothesis, we performed a pairwise statistical analysis. Divergence among mammals and vertebrates TRPVs was not the same (p-value <2.2·10^−16^). In mammals, divergence was extremely different between TRPV1-TRPV2, TRPV1-TRPV4, TRPV2-TRPV3, TRPV2-TRPV4 and TRPV3-TRPV4 (p-value <2.2·10^−16^), while divergence between TRPV1-TRPV3 was less dissimilar (p-value = 0.003). In vertebrates, divergence was extremely different between TRPV1-TRPV2, TRPV2-TRPV3 and TRPV2-TRPV4 (p-value <2.2·10^−16^), divergence was different between TRPV1-TRPV3 (p-value = 0.017) and there was no difference in divergence between TRPV1-TRPV4 and TRPV3-TRPV4 (p-value = 0.999).

**Figure 2:**
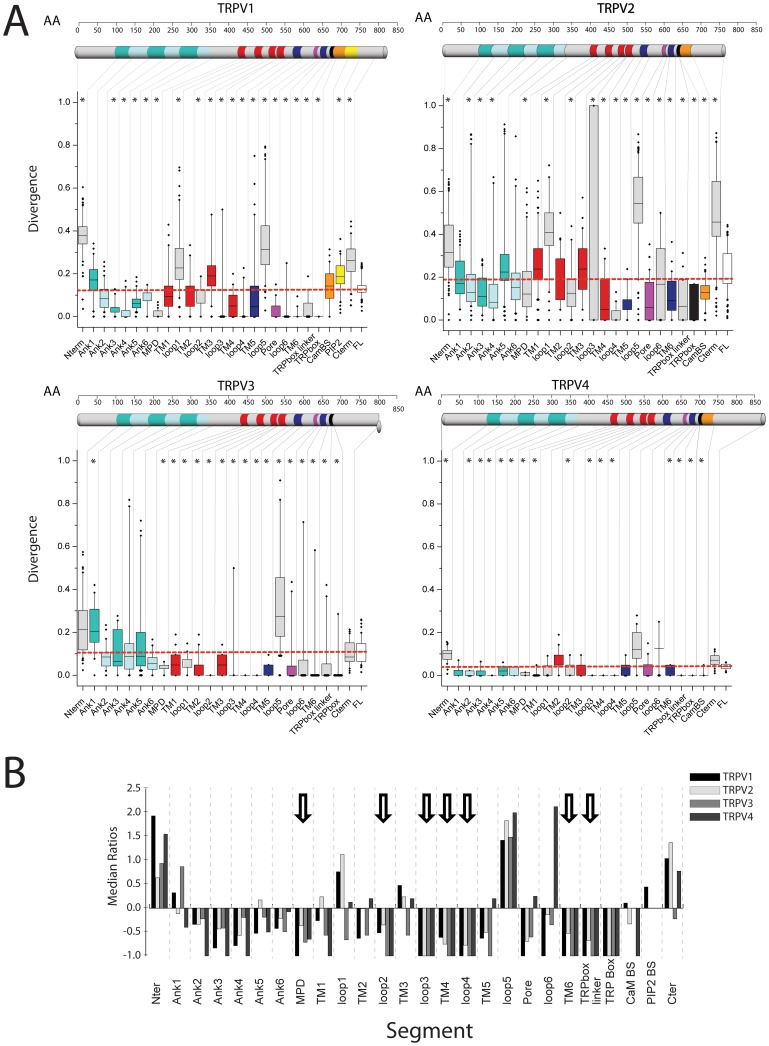
Domain-specific conservation profile for TRPV channels. **A.** Box plots showing the sequence divergence for specific domains of TRPV1-4 channels. The domain topology for the channels is indicated as a cartoon bar. The color-coding for each domain in the cartoon bar is represented in the box plot coloring. **B.** Plot of the normalized ratio for the medians for each domain segment. The Y-axis indicates the conservation (negative values) or divergence (positive values) of the domain in respect to the full-length protein conservation (value 0).

**Figure 3:**
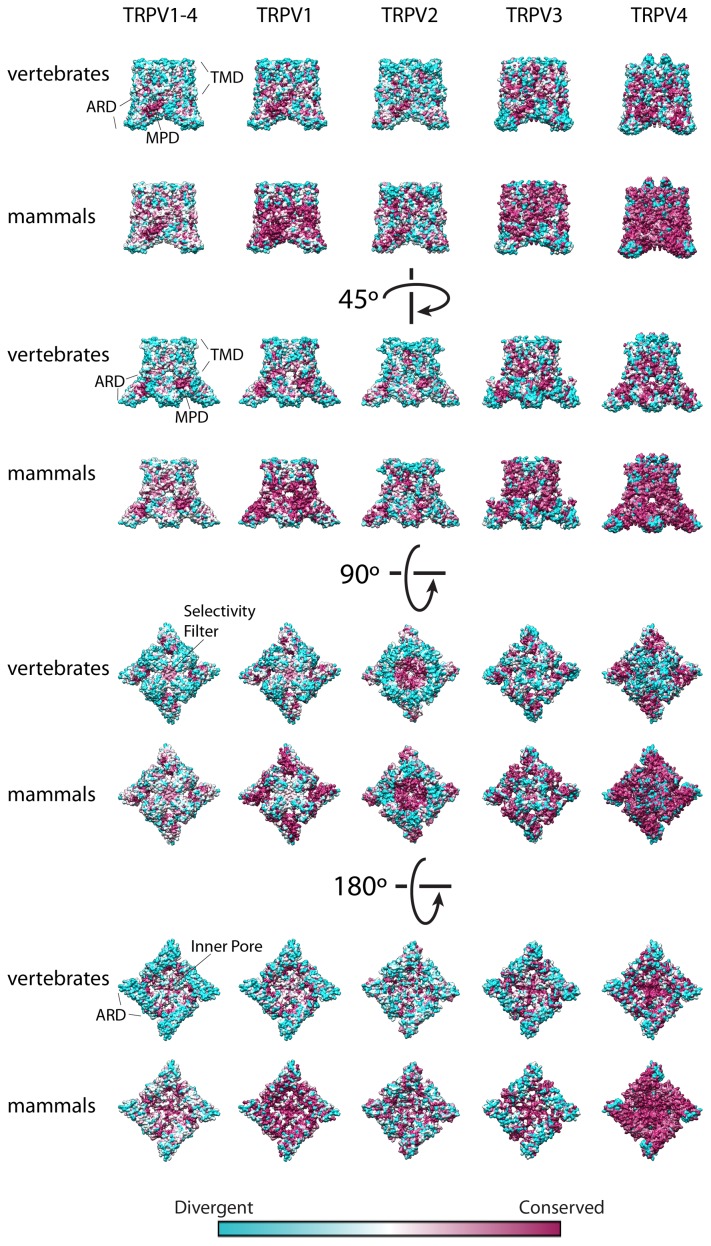
Tridimensional Conservation plots for TRPV1-4 comparing vertebrate and mammalian sequences. Conservation degree for each amino acid position was plotted on the solved structure for TRPV1 (pdb code 3J5P) for the MSAs for TRPV1-4 and TRPV1. For the conservation plot of TRPV2, TRPV3 and TRPV4 homology models were built based on the coordinates of TRPV1 (pdb code 3J5P). The conservation ranges from cyan (divergent) to magenta (conserved). Specific domains are indicated: TMD, transmembrane domain; ARD, ankyrind repeat domain; MPD, membrane proximal domain.

In Fig. 2A we show the evolutionary pressure exerted on specific domains (box plot for each domain, Table 2 for the list of domains) for mammalian TRPVs compared to the median of the evolutionary pressure exerted on the full-length mammalian TRPV sequences (dashed line). A statistically significant difference in the divergence of a specific domain compared to the full-length sequence is indicated with an asterisk (Fig. 2A). For clarity, we provide an additional representation highlighting the differences between positive *versus* purifying evolutionary pressures exerted over the different TRPV domains for mammals as a comparison of medians (Fig. 2B). Defining as a zero level the median value for the full-length TRPV sequence, we provide a ratio to identify the median positive values as divergent, and negative values as conserved for specific protein domains when compared to the full-length sequence (see Methods section for details). We indicate with an arrow the domains that show statistical differences (divergence/conservation) for all channels derived from the information in Fig. 2A. Since the high-resolution partial structure of TRPV1 has been solved [17], we had the opportunity to map the conservation profiles for the different channels onto the 3D TRPV1 structure or onto models based on this structure (Fig. 3).

**Table 2:** Segment definition for human TRPV1-4 channels based on UNIPROT details*.

Segment	hTRPV1	hTRPV2	hTRPV3	hTRPV4
**Nterm**	1–110	1–71	1–116	1–147
**Ank1** *	111–153	72–114	117–166	148–189
**Ank2** *	154–200	115–161	167–213	190–236
**Ank3** *	201–247	162–207	214–260	237–282
**Ank4** *	248–283	208–243	261–295	283–319
**Ank5** *	284–332	244–292	296–338	320–367
**Ank6** *	333–359	293–319	339–366	368–395
**MPD**	360–433	320–390	367–439	396–465
**TM1**	434–454	391–411	440–460	466–486
**loop1**	455–476	412–434	461–487	487–508
**TM2**	477–497	435–455	488–508	509–529
**loop2**	498–513	456–471	509–523	530–550
**TM3**	514–534	472–492	524–544	551–571
**loop3**	535	493	545	572
**TM4**	536–556	494–514	546–566	573–593
**loop4**	557–579	515–537	567–589	594–616
**TM5**	580–600	538–558	590–610	617–637
**loop5**	601–635	559–595	611–621	638–662
**pore**	636–647	596–609	622–641	663–682
**loop6**	648–659	610–621	642–649	683–690
**TM6**	660–680	622–642	650–670	691–711
**C-loop**	681–696	643–654	671–690	712–731
**TRPbox**	697–702	659–664	691–697	732–738
**Cam BS**	768–802	655–686		812–831
**PIP2**	778–793			
**Cterm**	803–839	687–764	698–790	832–871
**Total length**	839	764	790	871

*Ankyrin repeats were defined according to the crystal structure.

For TRPV1, the domains accounting for higher divergence than the full-length protein (positive selection) are the N-terminus, the 1^st^ extracellular loop, the TM3, the 5^th^ extracellular loop, the PIP2 binding domain and the distal C-terminus (Fig. 2A and Fig. 3). For TRPV2, the positive selection pressure is focused in the N-terminus, the 1^st^ extracellular loop, the 5^th^ extracellular loop, and the very distal C-terminus (Fig. 2A and Fig. 3). For TRPV3, which shows a high conservation profile, the positive selection pressure is focused in the first ankyrin repeat and in the 5^th^ extracellular loop (Fig. 2A and Fig. 3). For TRPV4, the lowest selection pressure is focused in the N-terminus (Fig. 2A and Fig. 3). In TRPVs the positive evolutionary pressure is exerted on the N-terminus and the loop 5 (although is not statistically significant for all the channels, Fig. 2B), while purifying evolutionary pressure is exerted over the membrane proximal domain (MPD), the 2^nd^ intracellular loop, the very short 3^rd^ extracellular loop, the 4^th^ transmembrane segment, the 4^th^ intracellular loop, the 6^th^ transmembrane segment, the TRP box linker (post TM6 segment) and the TRP box.

The TRPV1 structure and the TRPV2-4 models lack information on the N-terminus domain, the 5^th^ extracellular loop and the very distal C-terminus domain. Strikingly, to obtain the high-resolution structure of TRPV1, the 5^th^ extracellular loop (under positive selection) was removed from the sequence as part of the experimental design [17]. The very distal C-terminus domain of TRPV1 (also under positive selection) was not solved, probably because of protein proteolysis, indicating that these domains are not crucial in the TRPV scaffold.

### The transmembrane domain

**Figure 4:**
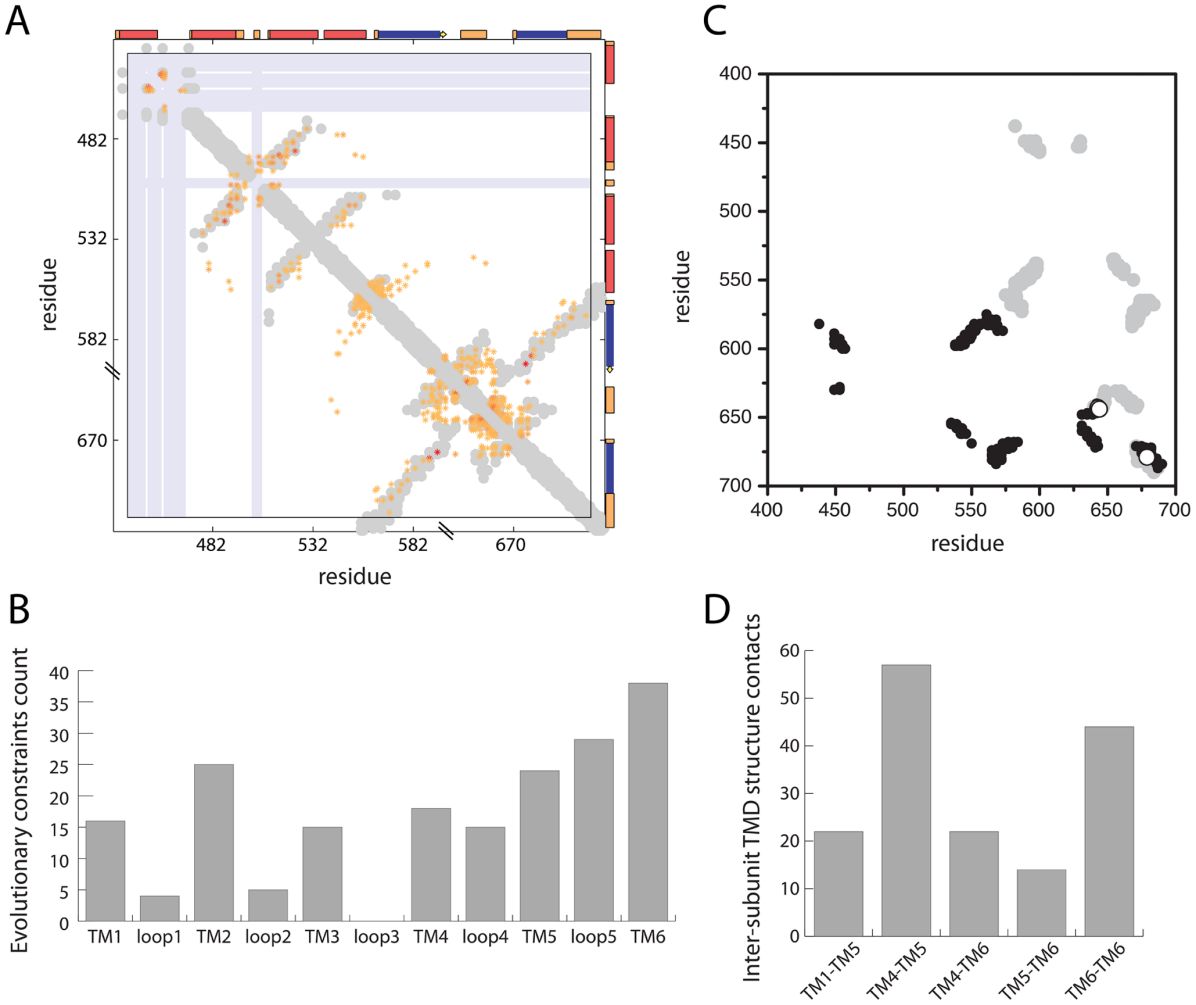
Transmembrane domain analysis for TRPV1-4. **A.** Evolutionary constraints heat map for the TMD of TRPVs using rat TRPV1 as reference sequence. The colored dots indicate the evolutionary constraints. The grey shaded area indicates the tridimensional structure contacts (3J5P). **B.** Histogram indicating the number of evolutionary constraints for each TMD region. **C.** Inter-chain structure contacts for chains A–B, black, for chains A–C, grey, and chains A–D, open circles. **D.** Histogram indicating the number of inter-chain structure contacts for chain A against chain B, C, and D between the indicated transmembrane segments.

The main oligomerization contacts for TRPV channels happen in the transmembrane domain (TMD). The contacts between the different subunits along the TM1-TM6 define the four-fold symmetry, the ion pore, and the selectivity filter. To map evolutionary traits on the TMD we have used EVcouplings [18] using the rat TRPV1 sequence as reference to compare the evolutionary constrains with the TRPV1 tridimensional structure. We have analyzed the evolutionary constraints (EC) on the TMD and the overlapping of the ECs (color dots) and the actual contacts (grey shaded area) in the tridimensional structure (Fig. 4A, Table S1 for a full set of ECs). The cloud of ECs is more disperse than the contacts defined by the tridimensional structure, indicating that the evolutionary traits in TRPV channels not only define the physical contacts. In Fig. 4B we have classified the ECs within the TMD domain. The TM5–TM6 region shows the highest number of ECs, followed by the TM2-loop 2 region. The TM1 and TM3 regions showed a similar number of ECs. To discriminate the residues involved in monomer-to-monomer contacts, we have mapped the subunit A (inter-chain) contacts from the PDB file (3J5P) in Fig. 4C. The contacts between chain A and B and chain A and chain C, are the same (in black and grey respectively). However, the contacts between chain A and chain D (open circles) are fewer and are located in the pore-forming region. Assuming four-fold symmetry, these contacts are equally defined for all subunit interactions (Table S2 for the full set of contacts for the TRPV1 TMD structure). The distribution of the inter-chain contacts corresponds to the TM1–TM5, TM4–TM5, TM4–TM6, TM5–TM6 and TM6–TM6 segments. The segments showing higher number of contacts are between TM4–TM5 and TM6–TM6 segments.

### Cytosolic domains

**Figure 5:**
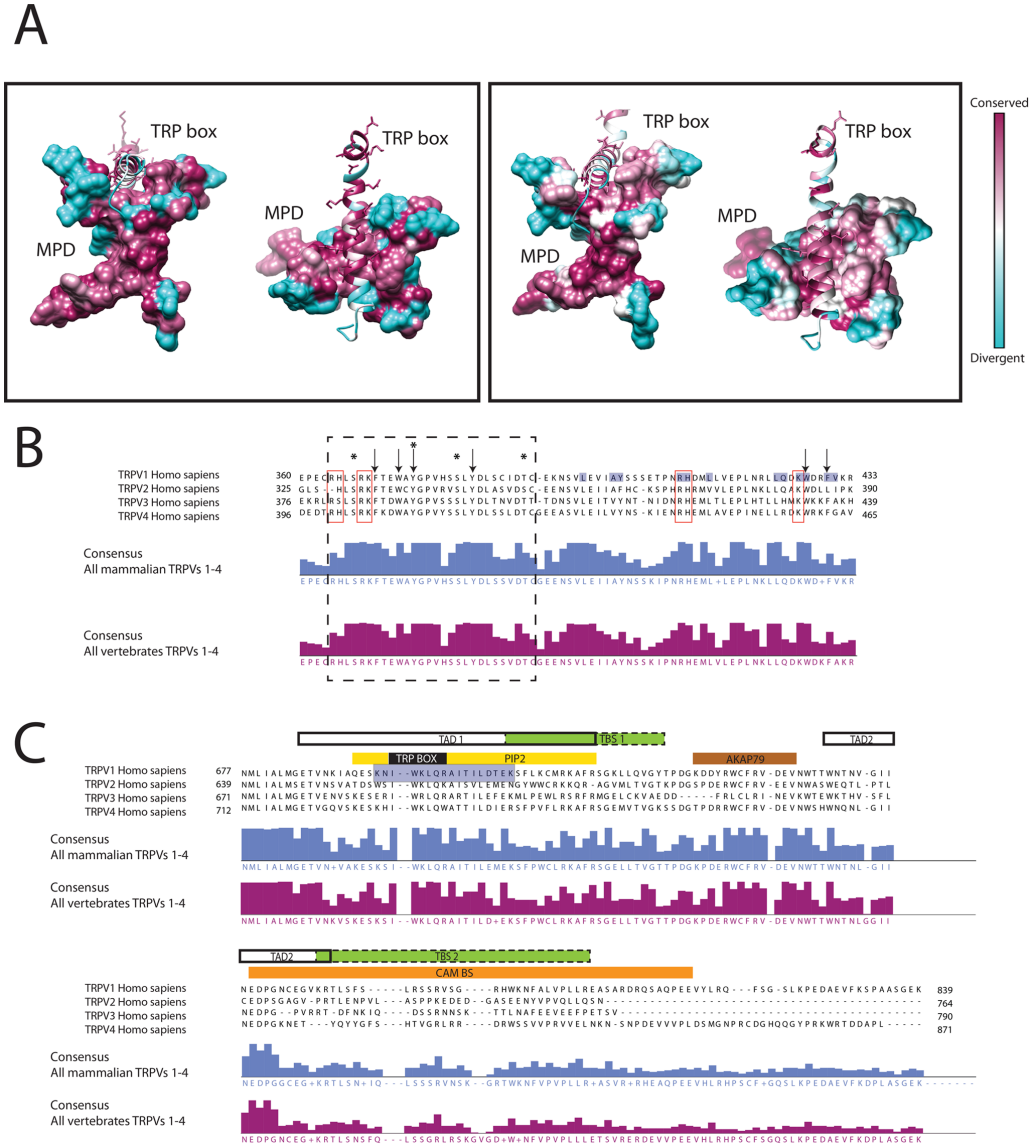
Cytosolic domains of TRPV channels. **A.** Conservation plot for the TRPV1 N-terminus (surface representation) and C-terminus (ribbon representation) interaction region. The residues involved in the MPD and TRP box interaction have been selected using a 5Å threshold. The specific residues are indicated in the alignments in Fig. 5B and 5C. The left plot represents the conservation scores obtained by ConSurf [46] for all TRPV1 sequences in this study. The plot in the right represents the conservation scores for all TRPV sequences used in this study. **B.** MSA for the membrane proximal domain of human TRPVs. The consensus sequence and the confidence score for the whole set of mammalian and vertebrate sequences are indicated. The black dashed line box delimits a conserved domain with predicted phosphorylation sites (asterisks, see text for details). Conserved residues are highlighted, positively charged residues are framed in red, and black arrows indicate aromatic residues. Shaded residues in the TRPV1 sequence represent the contacts between the MPD and the TRP box, represented in Fig. 5A. **C.** MSA of the C-terminal domain of human TRPV1-4 channels. Fragment showing the alignment of the C-terminal region from the human TRPV1-4 channels. The consensus sequence and the confidence score for the whole set of mammalian and vertebrate sequences are indicated. Specific structural/regulatory domains are indicated; TAD, tetramerization domain, commonly known as TRP domain; TBS, tubulin-binding sequence; PIP2, Phosphatidylinositol 4,5-bisphosphate binding sequence; AKAP79; CAM BS, calmodulin binding sequence. The alignments were plotted using JalView 2.8 [14]. Shaded residues in the TRPV1 sequence represent the contacts between the MPD and the TRP box, represented in Fig. 5A.

Recently the high-resolution structure for rat TRPV1 has been solved providing relevant tridimensional information [17]. The structure provides an exceptional illustration of intra-domain interaction, showing the interaction between the MPD and the TRP box depicted in Fig. 5A. Significantly, these two regions are highly conserved among all TRPVs, and not only within a TRPV isoform (Fig. 2 and 3), indicating a common molecular mechanism (Fig. 5A). From the tridimensional perspective, the membrane proximal region of the MPD domain acts as a fork where the TRP box slides during the gating mechanism. Considering the residue conservation (Fig. 5B and C) this seems to be a highly conserved mechanism among vertebrate TRPV1-4 channels. The MPD domain has been studied for TRPV1 and TRPV2, as a potential thermosensing module [19]. From the conservation perspective, biophysical features arise (Fig. 5B) such as the high consensus conservation of positive residues (R/K/H) close to conserved aromatic residues prone to partitioning at the water-membrane interface and promote protein-membrane interactions, thus acting as a lipid-binding domain. In this domain there are at least four highly conserved S/T/Y residues potentially phosphorylated (score over 0.5 by NetPhos algorithm [20]) indicating regulatory sites.

The C-terminus domain, comprising residues after TM6 until the end of the sequence, do not show the same level of conservation among channels as the N-terminus. Nevertheless, the C-terminal region of TRPV1 is one of the most characterized and some information can be cross-related among the different TRPV channels (Fig. 5C). TRPV1 contains two tubulin-binding sequences (TBS1 and TBS2). TBS1 falls within a TRPV1-4 rich positive-residue conserved region, whereas TBS2 is within a very low conservation region (TBS in Fig. 5C) [6], [21]. TRPV4 has been shown to bind microtubule-associated protein 7 in the last ∼60 C-terminal residues [22]. The tetramerization domains (TAD) are present in all TRPVs, overlapping with the highly conserved TRP box within the TRP domain [23], [24]. Derived from the recent structural information, the TRP domain has a tight relationship with the MPD. Concerning the PIP2 binding domain although initially described for TRPV1 [25], it was later described for TRPV2 [26] within the C-terminus. Due to the level of conservation of this region (which also includes the TRP box) and focusing on the conserved aromatic and positively-charged residues, the PIP2 domain may be easily defined for TRPV3 and TRPV4 as well [27].

Another protein-protein interaction region defined in TRPV1 is the one for the binding of AKAP79 protein [28], which can be easily translated into TRPV2, TRPV3 and TRPV4 because of the consensus sequence conservation (Fig. 5C). Finally, in Fig. 5C a gap appears in the middle of the highly conserved TRP box hallmark (IWKLQR consensus), indicating that one should be cautious about the poor quality of some non-reviewed sequences (outliers in Fig. 1C and D), which may introduce artifacts in the MSA.

## Discussion

Experimental structural data is essential for the understanding of membrane proteins’ molecular mechanisms. Bioinformatics provides tools to depict some functional/structural details using evolutionary information in the absence of structural data. Considering TRP channels as the subject for a bioinformatics approach represents a major challenge because the large number of protein members and the diverse functions. Here we have restricted our analysis to the TRPV1-4 evolutionary subset of TRP channels to gain structural insight into the multidomain organization and conservation of these channels. In addition, we have taken advantage on the recently solved structure for TRPV1 to validate specific evolutionary traits [17].

Considering the evolution time and sequence divergence parameters (Fig. 1), we can estimate the rate of evolutionary pressure. The purifying evolutionary pressure on TRPV1, TRPV3, and TRPV4 sequences is higher than on TRPV2 sequences, which are more divergent, either comparing mammals or vertebrates. Considering pressure across taxonomy, the highest purifying pressure has been exerted over TRPV4, although at very similar levels to TRPV1 and TRPV3. TRPV2 selective pressure indicates the possibility that TRPV2 channels are under positive selection, compared to TRPV1, TRPV3 and TRPV4. Another interesting hypothesis is that TRPV2 appeared as gene duplication from TRPV1 and positive selection on TRPV2 acts towards defining new physiological roles; thus, both channels (TRPV1 and TRPV2) may still have redundant roles on specific tissues/organisms. This hypothesis fits with the chromosome location of TRPV1, TRPV2 and TRPV3 in human (Chr17) and mouse (Chr11) for example, that indicates that a TRPV gene duplicated first originating TRPV2, and a more recent gene duplication generated the ancestor of TRPV1 and TRPV3 genes [29]. TRPV4 is located in chromosomes 12 and 5 in human and mouse, respectively, indicating an earlier/distinct genetic variation events.

TRPV1-4 subset is represented in the tree of life starting from teleosts [5] (Fig. 1), although representative TRPV ancestors are found in *Caenorhabditis elegans* and in *Drosophila melanogaster* [5], [30]–[32]. The late onset of these channels on the evolutionary tree of life argues for a very specific function requirement. Our analysis indicates that regions such as the distal N-terminus, some extracellular loops (such as the loop 5), and the distal C-terminus of TRPV1-4 channels are highly divergent, probably under positive selection, and very specific for each one of the channels. The putifying evolutionary pressure trend on specific domains, such as ARD, MPD, TMD and TRP domain (TRP box linker and TRP box) indicates that TRPV1-4 channels share a common minimal functional scaffold unit, comprising the ARD to the TRP domain, which corresponds to solved the high resolution tridimensional structure [17].

We have taken special consideration into the TMD of these channels, mainly because the channel gating that allows the cation flux through the pore, but also because the conservation profile of this domain will provide hints about homo/heteromer contacts, transmembrane topology and ligand binding. To get structural insight into the TMD we have analyzed the TMD residual coevolution pattern. Taken altogether, the evolutionary pressure on TM4–TM6 region is the highest, while the ECs in the TM1–TM3 region are not so represented (Fig. 4B). The evolution profile correlates with the inter-chain structure contacts: most of the inter-chain contacts in the TMD are located in the TM4–TM6 region, with the exception of the TM1–TM5 contacts (Fig. 4D). Analyzing the residues that may have co-evolved provides and interesting approach to understand the residues that may be in close vicinity. In the case of transmembrane proteins, the residual coevolution information can be cross-related to predict contacts between transmembrane segments involved in the folding/oligomerization of TPRV channels.

Using the primary sequence information we have delimited some conserved sequence determinants in the TRPV MPD domain that may be protein-protein and/or protein-lipid interaction modules (Fig. 5A and B). Although the MPD has been postulated to be the thermal sensing domain of the TRPV thermosensors [19], other roles for the MPD become evident, such as a lipid binding domain or a vesicle trafficking domain. Interestingly, the LKRSF consensus in the MPD sequence of TRPVs, with a highly likely phosphorylation serine site (Fig. 5) is also present near the coil domain of mammalian syntaxins 1,2, and 3, which also interact with the C2A domain of synaptotagmins [33]. The conserved LKRSF in TRPVs sequence is a predicted motif for PKC phosphorylation and it could be also involved in the regulation of syntaxin-binding proteins involved in vesicle-mediated transport. Vesicle trafficking may lead to translocation of TRPVs from internal membrane pools towards the plasma membrane, essential for TRPVs activity [34]–[36].

Similarly, the conservation of some functional features located in the C-terminus segment, allows the inference of domains such as the tetramerization, PIP2 binding and AKAP79 binding domains for all TRPVs 1–4 (Fig. 5C). It is noteworthy the importance of these domains for the correct assembly of the tetramer in the membrane, the gating and the regulation of the channel, respectively.

## Conclusions

Concerning TRPV1-4, we find that TRPV2 is under positive selection. The evolutionary pressure on specific domains is positive on the N-terminal and most extracellular loops, but negative in the ARD, TMD and the TRP domain. Taking advantage of the recently solved structure for TRPV1, we have been able to map specific evolutionary traits in the TMD that are relevant for the structure of the channel. We have also used the conservation profiles in the cytosolic domains to extrapolate functions of one channel to the rest, such as the AKAP interaction of TRPV1, the PIP2 binding of TRPV1 and TRPV2, etc.

From this study we find that evolutionary pressure is exerted differentially on different TRPVs but similarly on specific TRPV domains, arguing for a strong physiology/tissue-dependence/environment adaptability of these channels. From a methodological perspective, we provide a workflow for dissecting complex multidomain membrane proteins through their primary sequences, integrating and adapting state-of-the-art algorithms specifically for membrane proteins. In summary, our study highlights the relevance of evolutionary primary sequence analysis of membrane proteins towards predicting potential functional and structural sequence hallmarks, which may obviously require experimental validation.

## Materials and Methods

### Sequence retrieval and revision of draft sequences

We retrieved all the available sequences for TRPV1, TRPV2, TRPV3 and TRPV4 channels available from UNIPROT [13], [37]. The complete sequences from UNIPROT were used without further modifications. The fragment sequences were used as primers in genome databases NCBI [38] and UCSC [39] by BLAST and BLAT algorithms, respectively. Sequences identified in genomes were completed and used for further analysis. The fragment sequences that could not be completed, either because the genomic region was not covered or because they were not found, were not modified and the original UNIPROT retrieved fragments were used. The total set of sequences consisted of: TRPV1 (35 sequences), TRPV2 (35 sequences), TRPV3 (27 sequences), and TRPV4 (28 sequences). To avoid biased information, we used only the full-length sequences for the divergence analysis and conservation plots (Table 1 and Supplementary Information). Preliminary sequence analysis was carried out using the computational phylogenetics HyPhy software package [15]. To detect directional selection we performed a two-step process: first, we run the “Model Selection Tool” and the best model for our data was Jones-Taylor-Thornton (JTT). Second we performed a FADE analysis for each TRPV channel MSA (rooting the alignment in the *Homo sapiens* sequence) to detect sites under evolutionary directional positive selection (output in Table 1). As a measure of positive selection we chose the residues with a posterior confidence interval >0.95.

### Multiple sequence alignment

All the sequences were aligned using ClustalW algorithm with a gap opening penalty of 10 and a gap extension penalty of 0.1 [40]. For validation of the alignments, we have also used MAFFT alignments [41]; there were no major differences between the generated MSAs. We defined two subsets of MSAs, to discriminate between mammalian and vertebrate sequences for TRPV1, TRPV2, TRPV3 and TRPV4.

### Phylogenetic tree generation

Trees were generated by maximum likelihood algorithm (Nearest-Neighbour-interchange heuristic method). A JTT model with uniform rates was used to calculate amino acid substitutions. Bootstrapping method with 250 iterations was used to improve the phylogenetic tree confidence value.

### Box plot generation

Using the full protein or the specific segment MSAs we build a pairwise sequence divergence matrix in MEGA5.0. Considering one specific TRPV channel, all species sequences within the specific member were compared in a pairwise fashion obtaining a divergence matrix. Specifically, we applied a p-distance model, where the pairwise divergence among all the sequences in one TRPV member subset is calculated considering the number of substitutions per total number of residues considered (full length protein or specific segment). Any amino acid substitution is scored with equal distance (uniform rate), and alignment gaps are considered partial deletions sites and are removed from any calculations, i.e. sites present in all sequences are considered.

Medians of divergence in mammal and vertebrate TRPVs were compared with Kruskal-Wallis test. Divergence of each TRPV was compared with divergence of the other TRPVs using Wilcoxon rank sum test. p-values were adjusted for multiple comparisons using the Bonferroni correction and considered significant (*) when p-value<0.05. Statistical analysis was performed with R Statistical Package (Version 2.15.1) [42].

The sequences of human TRPV1, TRPV2, TRPV3 and TRPV4 were used as references for domain definition. MSAs were truncated into different segments attending to UNIPROT topology definition for the different transmembrane segments and functional domains. The ankyrin repeats were defined attending to the crystal structures already available [43]–[45]. The divergence matrices for specific segments were generated as previously indicated for the full-length sequences. The distances (divergence) within the box represent the first three quartiles and the line corresponds to the median (second quartile). The whiskers indicate the values within 1.5 times the interquartile range from the lowest and highest quartile, respectively. Diamonds represent outliers. For statistical analysis of the divergence of specific segments, we used a Mann-Whitney comparison of two medians (segment *versus* full-length).

Boxplots were generated both for full sequences and for specific domains. We have used a normalized ratio of medians (Fig. 2B) to provide a qualitative comparison for the analysis of divergence between full-length sequences and specific domains, among the different channels:

(Segment median – Full length median)/(Full length median).

### Evolutionary constraints and conservation plots

To map residue and domain conservation on the specific domains, we used the ConSurf server [46] using the 3J5P TRPV1 structure as template. Evolutionary constraints of the TRPVs TMD domain were predicted using the EVcouplings server [18] and the inter-chain crystal contacts for rat TRPV1 structure, PDB code 3J5P [17] were analyzed using the contact map analysis tool from the SPACE suite [47].

### Tridimensional representations and modeling

All structural representations have been performed using UCSF Chimera [48]. The modeling of TRPV2, TRPV3, and TRPV4 in Figure 3 has been carried out using the MODELLER suite [49] included in UCSF Chimera.

## Supporting Information

Table S1 **Output file from the EVcouplings algorithm indicating the evolutionary constraints for rat TRPV1 transmembrane domain segment.**
(CSV)

Table S2 **Inter-chain structure contacts for chain A obtained for the rat TRPV1 transmembrane domain segment high-resolution structure (PDB code 3J5P).**
(CSV)

Dataset S1 **Full list of sequences used in this analysis.**
(PDF)
