# Supplementary material for: Dissecting Domain-Specific Evolutionary Pressure Profiles of Transient Receptor Potential Vanilloid Subfamily Members 1 to 4
Source: PLoS One. 2014 Oct 21;9(10):e110715. doi: 10.1371/journal.pone.0110715 (PMC4204936; doi:10.1371/journal.pone.0110715)
Supplement: Dataset S1 — Full list of sequences used in this analysis. (PDF) [file pone.0110715.s003.pdf]

>TRPV1_Rattus_norvegicus

MEQRASLDSEESESPPQENSCLDPPDRDPNCKPPPVKPHIFTTRSRTRLFGKGDSEEAS
PLDCPYEEGGLASCPIITVSSVLTIQRPGDGPASVRPSSQDSVSAGEKPPRLYDRRSIFDA
VAQSNCQELESLLPFLQRSKKRLTDSEFKDPETGKTCLLKAMLNLHNGQNDTIALLLD
VARKTDSLKQFVNASYTDSYYKGQTALHIAIERRNMTLVTLLVENGADVQAAANGDF
FKKTKGRPGFYFGELPLSLAACTNQLAIVKFLLQNSWQPADISARDSVGNTVLHALVE
VADNTVDNTKFVTSMYNEILILGAKLHPTLKLEEITNRKGLTPLALAASSGKIGVLAYIL
QREIHEPECRHLSRKFTEWAYGPVHSSLYDLSCIDTCEKNSVLEVIAYSSSETPNRHDM
LLVEPLNRLLQDKWDRFVKRIFYFNFFVYCLYMIIFTAAAYYRPVEGLPPYKLKNTVG
DYFRVTGEILSVSGGVYFFFRGIQYFLQRRPSLKSLFVDSYSEILFFVQSLFMLVSVVLYF
SQRKEYVASMVFSLAMGWTNMLYYTRGFQQMGIYAVMIEKMILRDLCRFMFVYLVFL
FGFSTAVVTLIEDGKNNSLPMESTPHKCRGSACKPGNSYNSLYSTCLELFKFTIGMGDL
EFTENYDFKAVFIILLLAYVILTYILLLNMLIALMGETVNKIAQESKNIWKLQRAITILDT
EKSFLKCMRKAFRSGKLLQVGFTPDGKDDYRWCFRVDEVNWTTWNTNVGIINEDPG
NCEGVKRTLSFSLRSGRVSGRNWKNFALVPLLRDASTRDRHATQQEEVQLKHYTGSL
KPEDAEVFKDSMVPGEK---

>TRPV1_Sus_scrofa

MKKWESLDAGESQQDSSPHPQDGDPTLRPAPAKPHAFPAVESHSQRFGKGDSEDASL
VGCSYEEGQLASCPAITVSPVVIIQKPGDGPTCARQPSQDSVDSENFKLYDRKKIFEAVA
QNNCEALESLLLFLQKSKKHLVDSEFKDPETGKTCLLKAMLNLHNGQNETIPLLLEIA
RQTDSLKELVNASYTDSYYKGQTALHIAIERRNMALVTLLVENGADVQAAANGDFFK
KTKGRPGFYFGELPLSLAACTNQLGIVKFLLQNSWHPADISARDSVGNTVLHALVEVA
DNTADNTKFVTSMYNEILILGARLHPTLKLEELTNKKGLTPLALAAKSGKIGVLAYILQ
REIQEPECRHLSRKFTEWAYGPVHSSLYDLSCIDTCEKNSVLEVIAYSSSETPNRHDML
LVEPLNRLLQDKWDRFVKRIFYFNFLVYCLYMIIFTTAAYYRPTEGLPPFKLNHTVGD
YFRVIGEILSVVGGIYLFFRGIQYFLQRRPSLKTLFVDSYSEMLFFVQSLFMLGTVVLYFC
HRKEYVASMVFSLAMGWANMLYYTRGFQQMGIYAVMIEKMILRDLCRFMFVYLVFL
FGFSTAVVTLIEDGKNDSVSTEVFHKCRGLVCRSPDSSYNSLYSTCLELFKFTIGMGDLE
FTENYDFKAVFIILLLAYVILTYILLLNMLIALMGETVNKISQESKNIWKLQRAITILDTE
KSFLKCMRKVFRSGKLLQVGYTPDGKDDYRWCFRVDEVNWTTWNTNVGIINEDPGN
CEGIKRTLSFSLRSSRVAGRNWKNFALVPLLRDASTRERHLAQPEEVHLKHIAKSLKP
EDAEIVKDP---

>TRPV1_Bos_taurus

MKKWGSSESRESQDLPQEDSCPDPLDGDPNYRPAPTKPHSFPTAKSRSRLFGKGDSE
DTSLMDCSYEEGQLASCPAITISPVVIIQRSGDGPTCVRQLSQDSAATENLKLYDRRKIF
EAVAQNNCEELESLLLFLQKSKKHLMDSEFKDPETGKTCLLKAMLNLHNGQNDTIPL
LLEIARQTDSLKELVNASYTDSYYKGQTALHIAIERRNMALVTLLVENGANVQAAANG
DFFKKTKGRPGFYFGELPLSLAACTNQLGIVKFLLQNSWQPADISARDSVGNTVLHAL
VEVADNTADNTKFVTSMYNEILILGAKIHPTLKLEELTNKKGLTPLALAARSGKIGVLA
YILQREIQEPECRHLSRKFTEWAYGPVHSSLYDLSCIDTCEKNSVLEVIAYSSSETPNRH
DMLLVEPLNRLLQDKWDRFVKRIFYFNFFVYCLYMIIFTTVAYYRPAGGRPPFKPKHT
VGDYFRITGEIISVAGGIYFFSRGIQYFLQRRPSLKTLFVDSYSEMLFFMQSLFMLATVV

LYFCHRKEYVASMVFSLAMGWTNMLYYTRGFQQMGIYAVMIEKMILRDLCRFMFVYL
VFLFGFSTAVVTLIEDEKNDSVSVELSQHRWRGHGCRSADSYNSLYSTCLELFKFTIGM
GDLEFTENYDFKAVFVILLLAYVILTYILLLNMLIALMGETVNKIAQESKNIWKLQRAIT
ILDTEKSFLKCMRKAFRSGKLLQVGYTPDGKDDYRWCFRVDEVNWTTWNTNVGIIN
EDPGNCEGIKRTLSFSLRSSRVAGRNWKNFALVPLLRDASTRERHPAQPEEVHLRHFT
GSLKPEDAEIINDSVALGEK---

>TRPV1_Canis_familiaris

MKNWGSSDSGGSEDPPQEDSCLDPLDGDPNSRPVPAKPHIFPTAKSRSRLFGKCDSEE
ASMDCSYEEGQLASCPAITVSPVVMIPKHEDGPTCARQPSQDSVTAGSEKSLKLYDRR
KIFEAVAQNNCEELQSLLLFLQKSKKHLMDSEFKDPETGKTCLLKAMLNLHDGQNDTI
PLLLEIARQTDSLKELVNASYTDSYYKGQTALHIAIERRNMALVTLLVENGADVQAAA
NGDFFKKTKGRPGFYFGELPLSLAACTNQLGIVKFLLQNSWQPADISARDSVGNTVLH
ALVEVADNTADNTKFVTSMYNEILILGAKLHPTLKLEGLTNKKGLTPLALAARSGKIG
VLAYILQREIQEPECRHLSRKFTEWAYGPVHSSLYDLSCIDTCEKNSVLEVIAYSSSETP
NRHDMLLVEPLNRLLQDKWDRFVKRIFYFNFFIYCLYMIIFTTAAYYRPVDGLPPYKL
KHTVGDYFRVTGEILSVLGGVYFFFRGIQYFLQRRPSLKTLFVDSYSEMLFFVQSLFML
GTVVLYFCHHKEYVASMVFSLAMGWTNMLYYTRGFQQMGIYAVMIEKMILRDLCRF
MFVYLVFLFGFSTAVVTLIEDGKNNSVPTESTLHRWRGPGCRPPDSSYNSLYSTCLELF
KFTIGMGDLEFTENYDFKAVFIILLLAYVILTYILLLNMLIALMGETVNKIAQESKNIWK
LQRAITILDTEKSFLKCMRKAFRSGKLLQVGYTPDGKDDYRWCFRVDEVNWTTWNT
NVGIINEDPGNCEGIKRTLSFSLRSGRVSGRNWKNFSLVPLLRDASTRERHPAQPEEV
HLRHFAGSLKPEDAEIFKDPVGLGEK---

>TRPV1_Cavia_porcellus

MKKRASVDSKESEDPPQEDYSLDPLDVDANSKTPPAKPHTFSVSKSRNRLFGKSDLEE
SSPIDCSFREGEAASCPTITVSSVVTSPRPADGPTSTRQLTQDSIPTSAEKPLKLYDRRSI
FDAVAQNNCQDLDSLLPFLQKSKKRLTDTEFKDPETGKTCLLKAMLNLHNGQNDTIS
LLLDIARQTNSLKEFVNASYTDSYYRGQTALHIAIERRNMVLVTLLVENGADVQAAAN
GDFFKKTKGRPGFYFGELPLSLAACTNQLAIVKFLLQNSWQPADISARDSVGNTVLHA
LVEVADNTADNTKFVTSMYNEILILGAKLYPTLKLEELTNKKGFTPLALAASSGKIGVL
AYILQREIPEPECRHLSRKFTEWAYGPVHSSLYDLSCIDTCEKNSVLEVIAYSSSETPNR
HDMLLVEPLNRLLQDKWDRFVKRIFYFNFFIYCLYMIIFTMAAYYRPVDGLPPYKMKN
TVGDYFRVTGEILSVIGGFHFFFRGIQYFLQRRPSVKTLFVDSYSEILFFVQSLFLLASVV
LYFSHRKEYVACMVFSLALGWTNMLYYTRGFQQMGIYAVMIEKMILRDLCRFMFVYL
VFLFGFSTAVVTLIEDGKNESLSAEPHRWRGPGCRSAKNSYNSLYSTCLELFKFTIGMG
DLEFTENYDFKAVFIILLLAYVILTYILLLNMLIALMGETVNKIAQESKNIWKLQRAITIL
DTEKSFLKCMRKAFRSGKLLQVGYTPDGKDDYRWCFRVDEVNWTTWNTNVGIINED
PGNCEGVKRTLSFSLRSGRVSGRNWKNFALVPLLRDASTRDRHSAQPEEVHLKHFSGS
LKPEDAEVFKDSAVPGEK---

>TRPV1_Desmondus_rotundus

MKKRGSSDSGESEDLPQEDSLPDPLDGNPHSRPAPAKSNILMAKRRRRLFGKGDSEEA
SPQDCSYKEGHPASCPAITVSPVLTVQKTEDGPTCTWQLCQDSVPAGTKKCLKLYDRR
KIFEAVAQNNCEELESLLLFLQKSKKHLMDREFKDPETGKTCLLKAMLNLHNGQNNT
IPLLLEIARQTDSLKELVNASYTDSYYKGQTALHIAIERRNMALVTLLVENGADVQAAA
NGDFFKKTKGRPGFYFGELPLSLAACTNQLGIVKFLLQNSWQPADISARDSVGNTVLH
ALVEVADNTADNTKFVTSMYNEILILGAKLHPTLKLEELINKKGLTPLALAASSGKIGV
LAYILQREIHEPECRYLSRKFTEWAYGPVHSSLYDLSCIDTCEKNSVLEVIAYSSNETPN
RHDMLLVEPLNRLLQDKWDRFVKRIFYFNFFVYCLYMIIFTTVAYYRPVEGLPPYKVK
HTVGDYFRVIGEILSVLGGVYFFFRGIQYFLQRRPSLKSLFVDSYSEILFFVQSLFMLGTV
VLYFCQCKEYVASMVFSLAMGWTNMLYYTRGFQQMGIYAVMIEKMILRDLCRFMFVY
LVFLFGFSTAVVTLIEDGKNDSVSTETTPHRWRGPCYRSPDSSYNSLYSTCLELFKFTIG
MGDLEFTENYDFKAVFIILLLAYVILTYILLLNMLIALMGETVNKIAQESKNIWKLQRAI
TILDTEKSFLKCMRKAFRSGKLLQVGYTPDGKDDYRWCFRVDEVNWTTWNTNVGII
NEDPGNCEGIKRTLSFSLRSSRVAGRNWKNFALVPLLRDASTRERQPAQPEEVHLKHF
SGSLKPEDAXVFKDSAALGESEKGESTV--

>TRPV1_Equus_caballus

MKKWGSSDSGDSEDLPQEDSCPDPQDGDPDFRPPPAKPPIFPVTKSHSQLFGKCGLVE
ASSTDCSYEEGHPASCPAITVSPVVIIQRPGDGPICTRQPSQDSVTSGAQKDLYDRRKIF
EAVAQNNCEELENLLFFLQKSKKHLMDSEFKDPETGKTCLLKAMLNLHDGNDTIPLL
LEIARQTDSLKELVNASYTDSYYKGQTALHIAIERRNMTLVTLLVENGADVQAAANGD
FFKKTKGRPGFYFGELPLSLAACTNQLGIVKFLLQNSWQPADISARDSVGNSVLHALVE
VADNTADNTKFVTSMYNEILILGAKLHPTLKLEELTNKKGLTPLALAAGSGKIGVLAYI
LQREIRDPECRHLSRKFTEWAYGPVHSSLYDLSCIDTWEKNSVLEVIAYSSSETPNRQD
MLLVEPLNRLLQDKWDRFVKRIFYFNFFVYCLYMIIFTTVAYYRPVDGLPPFKLNTVG
DYFRVTGEILSVSGGVYFFFRGIQYFLQRRPSLKTLFVDSYSEMLFFVQSLFMLGTVVLY
FCHCKEYVASMVFSLAMGWTNMLYYTRGFQQMGIYAVMIEKMILRDLCRFMFVYLVF
LFGFSTAVVTLIEDGKNDSVLAESTSHRWRGHGCRSPDSSYNSLYSTCLELFKFTIGMG
DLEFTENYDFKAVFIILLLAYVILTYILLLNMLIALMGETVNKISQESKNIWKLQRAITIL
DTEKSFLKCIRKAFRSGKLLQVGYTPDGKDDYRWCFRVDEVNWTTWNTNVGIINEDP
GNCEDIKRTLSFSLRSGRVSGRNWKNFSLAPLLREASVRERHPAPPEEVNLRHFAGSL
KPEDAEICKDPAASGEK---

>TRPV1_Heterocephalus_glaber

MKKWASIDSRESEHPPQEEDSSLDPPDTDANSKTPPAKPHIFSVSKSRTRLFGKGDSE
ELSPMDCSYEEGEPVSCPTITVSSVVISPRPGDGPTCARQLSQDSIPASAEKPLKLYDRR
SIFDAVAQNNCQELDSLLPFLKKSKKRLTDTEFKDPETGKTCLLKAMLNLHNGQNDTI
SLLLDIAQETDSLREFVNASYTDSYYKGQTALHIAIERRNMALVTLLVENGADVQAAA
NGDFFKKTKGRPGFYFGELPLSLAACTNQLAIVKFLLQNSWQPADISARDSVGNTVLH
ALVEVADNTADNTKFVTSMYNDILILGAKLYPTLKLEEITNKKGFTPLALAASSGKIGV
LAYILQREIPEPECRHLSRKFTEWAYGPVHSSLYDLSCIDTCEKNSVLEVIAYSSSETPN
RHDMLLVEPLNRLLQDKWDRFVKRIFYFNFFIYCLYMIIFTTAAYYRPVDGLPPYKLK
NTVEDYFRVTGEILSVTGGVYFFFRGIQYFLQRRPTMKTLFVDSYSEILFFVQSLFMLVS
VVLYFSHRKEYVASMVFSLALGWTNMLYYTRGFQQMGIYAVMIEKMILRDLRRFMFV
YLVFLFGFSTAVVTLIEDGKNDSLPIESHRWRGPSCRTSSNSYNSLYSTCLELFKFTIGM

GDLEFTENYDFKAVFIILLLAYVILTYILLLNMLIALMGETVNKIAQESKNIWKLQRAITI
LDTEKSFLKCMRKAFRSGKLLQVGYTPDGKDDYRWCFRVDEVNWTTWNTNVGIINE
DPGNCEGVKRTLSFSLRSGRVSGRNWKNFALVPLLRDASTRDRHSSHPDEVHLKHFS
GSLKPEDAEVFKDSVAPGEK---

>TRPV1_Scapanus_oratius

MKKWGNSDAGESEDPPQEDSCPEPLDEDPNFRPALVKSHFFPTAKSRSRLFGKCDSE
EASPMDCSYEEGELSSCPAITVSPAVVTQRPGDGPACARQLFQNCVPVGAKKNLKLYD
RRKIFEAVAQNNCEELESLLLFLQKSKKHLMDSEFKDPETGKTCLLKAMLNLHDGQN
DTIPLLLEIARQTDSLREFVNASYTDSYYKGQTALHIAIERRNMPLVTLLVENGADVQA
AANGDFFKKTKGRPGFYFGELPLSLAACTNQLGIVKFLLQNSWQPADISARDSVGNTV
LHALVEVADNTAENTKFVTSMYSEILILGARLHPTLKLEELTNKKGLTPLALAAKSGKI
GVLAYILQREIQEPECRHLSRKFTEWAYGPVHSSLYDLSCIDTCEKNSVLEVIAYSSSET
PNRHDMLLVEPLNRLLQDKWDRFVKRIFYFNFFVYCLYMIIFTTAAYYRPVEGLPPYK
MNHTVADYFRVTGEILSVSGGVYFFFRGIQYFLQRRPSLKTLFVDSYSEMLFFVQSLFM
LGTVALYFSHRKEYVASMVFSLAMGWTNMLYYTRGFQQMGIYAVMIEKMILRDLCRF
MFVYLVFLFGFSTAVVTLIEDGKNNSVQAELISHRWRGPNCRQPDSSYNSLYSTCLELF
KFTIGMGDLEFTENYDFKAVFIILLLAYVILTYILLLNMLIALMGETVNKIAQESKNIWK
LQRAITILDTEKSFLKCMRKAFRSGKLLQVGYTPDGKDDYRWCFRVDEVNWTTWNT
NVGIINEDPGNCEGIKRTLSFSLRSGRVSGRNWKNFTLVPLLRDASTRERHPAQPEEV
HLRHFAGSLKPEDAEVFKDPAAFGEK---

>TRPV1_Homo_sapiens

MKKWSSTDLGAAADPLQKDTCPDPLDGDPNSRPPPAKPQLSTAKSRTRLFGKGDSEE
AFPVDCPHEEGELDSCPTITVSPVITIQRPGDGPTGARLLSQDSVAASTEKTLRLYDRRS
IFEAVAQNNCQDLESLLLFLQKSKKHLTDNEFKDPETGKTCLLKAMLNLHDGQNTTIP
LLLEIARQTDSLKELVNASYTDSYYKGQTALHIAIERRNMALVTLLVENGADVQAAAH
GDFFKKTKGRPGFYFGELPLSLAACTNQLGIVKFLLQNSWQTADISARDSVGNTVLHA
LVEVADNTADNTKFVTSMYNEILMLGAKLHPTLKLEELTNKKGMTPLALAAGTGKIG
VLAYILQREIQEPECRHLSRKFTEWAYGPVHSSLYDLSCIDTCEKNSVLEVIAYSSSETP
NRHDMLLVEPLNRLLQDKWDRFVKRIFYFNFLVYCLYMIIFTMAAYYRPVDGLPPFK
MEKTGDYFRVTGEILSVLGGVYFFFRGIQYFLQRRPSMKTLFVDSYSEMLFFLQSLFML
ATVVLYFSHLKEYVASMVFSLALGWTNMLYYTRGFQQMGIYAVMIEKMILRDLCRFM
FVYIVFLFGFSTAVVTLIEDGKNDSLPSESTSHRWRGPACRPPDSSYNSLYSTCLELFKF
TIGMGDLEFTENYDFKAVFIILLLAYVILTYILLLNMLIALMGETVNKIAQESKNIWKLQ
RAITILDTEKSFLKCMRKAFRSGKLLQVGYTPDGKDDYRWCFRVDEVNWTTWNTNV
GIINEDPGNCEGVKRTLSFSLRSSRVSGRHWKNFALVPLLREASARDRQSAQPEEVYLR
QFSGSLKPEDAEVFKSPAASGEK---

>TRPV1_Putorius_furo

MKKWGSSDSGKAEDPSQEDSCPDPLDGDPNSRSAPAKPHFFPTAKSRSRLFGKCDSE
EASPMDCSYEEGQLASCPAITVNPVVIIQRHEDGPTCARQLSQDSVTAGSEKSLKLYDR
RKIFEAVAQNNCEELQSLLLFLQRSKKHLMDSEFKDPETGKTCLLKAMLNLHEGQND

TIPLLLEIARQTDSLKELVNASYTDSYYKGQTALHIAIERRNMALVTLLVENGADVQAA
ANGDFFKKTKGRPGFYFGELPLSLAACTNQLGIVKFLLQNSWQPADISAKDSVGNTVL
HALVEVADNTADNTKFVTSMYNEILILGAKLHPTLKLEELTNKKGLTPLALAARTGKI
GVLAYILQREIQEPECRHLSRKFTEWAYGPVHSSLYDLSCIDTCEKNSVLEVIAYSSSET
PNRHDMLLVEPLNRLLQDKWDRFVKRIFYFNFFVYCLYMIIFTTAAYYRPVDGLPPYK
LKHTIGDYFRVTGEILSVSGGVYFFFRGIQYFLQRRPSLKTLFVDSYSEMLFFVQSLFML
GTVVLYFCHHKEYVASMVFSLAMGWTNMLYYTRGFQQMGIYAVMIEKMILRDLCRF
MFVYLVFLFGFSTATTTILKSTSGKSLGLDTTSLMGGPAPTGPGRQQPSLGRSGLEGLS
LECAPAKLQKRHERSCSGALGLGLERLILEAPLTARGSPGISPLPRAITILDTEKSFLKC
MRKAFRSGKLLQVGYTPDGKDDYRWCFRVDEVNWTTWNTNVGIINEDPGNCEGIKR
TLSFSLWSGRGEA--

>TRPV1_Loxodonta_africana

MKKWNSSDLGKSEDPPQEATCPHSPDDDPDSKPPPTEPQIFMAKSRPRLFGKGDSEE
ASQVSHVHCSYEEGELAYCPAITVSPAVIIKRPGDGPACARQLSQDSVTAGTTKKTLYD
RRRIFDAVAQNDCEELASLLLFLQKSKKHLMDCEFKDPETGKTCLLKAMLNLHDGQN
DTIPLLLEIARQTDSLKELVNASYTDSYYKGQTALHIAIERRNMALVTLLVENGADVQA
AANGDFFKKIKGRPGFYFGELPLSLAACTNQLGIVKFLLQNSWQPADISARDSMGNTV
LHALVEVADNTTDNTKFVTSMYNDILILGAKLHPKLKLEELTNKKGLTPLALAASSGKI
GVLAYILQREIQEPECRHLSRKFTEWAYGPVHSSLYDLSCIDTCEKNSVLEVIAYSSSET
PNRHDMLLVEPLNRLLQDKWDRFVKRIFYFNFFIYCLYMIIFTMAAYNRPGDGLPPFK
VNNTEDYFRVTGETLSVSGGVYFFFRGIQYFLQRRPSIKSLFVDSYSEILFFVQSIFMLGT
VVLYFSHRKEYVASMVFSLAMGWTNMLYYTRGFQQMGIYAVMIEKMILRDLCRFMF
VYVVFLFGFSTAVVTLIEEGNKKPAAAEPTSHRWRGPCCRPPDNYNSLYSTCLELFKFT
IGMGDLEFTENYEFKAVFIILLLAYVILTYILLLNMLIALMGETVNKIAQESKNIWKLQR
AITILDTEKSFLKCMRKAFRSGKLLQVGYTPDGKDDYRWCFRVDEVNWTTWNTNVGI
INEDPGYCKGVKRTLSFSLRPNKVSGRNWKNFGLVPLLRDASTRERPAPQPEDVHLK
HFAGSLKPEDAEVFRDSAAFREK---

>TRPV1_Macaca_mulatta

MKKWSSTDSGAAADPLQQDACPDPPDGDPNSRPPPAKPQLSTAKSRTRLFGKGDSEE
AFLVDCPHEEGELASCPTITVSPVITIQRPGDGPTGARLPSQDSVATSTERTLRLYDRRS
IFEAVAQNNCQDLESLLLFLQKSEKHLTDNEFKDPETGKTCLLKAMLNLHDGQNDTIP
LLLEIARQTGSLKEFVNASYTDSYYKGQTALHIAIERRNMGLVTLLVENGADVQAAAN
GDFFKKTKGRPGFYFGELPLSLAACTNQLGIVKFLLQNSWQPADISARDSVGNTVLHA
LVEVADNTADNTKFVTSMYNEILILGAKLHPTLKLEELTNKKGMTPLALAAGTGKIGV
LAYILQREIQEPECRHLSRKFTEWAYGPVHSSLYDLSCIDTCEKNSVLEVIAYSSSETPN
RHDMLLVEPLNRLLQDKWDRFVKRTFYFNFLVYCLYMIIFTMAAYYRPVDGLPPFKM
EKTEDYFRVTGEILSVLGGVYFFFRGIQYFLQRRPSMKTLFVDSYSEMLFFLQSLFMLA
TVVLYFSHLKEYVASMVFSLALGWTNMLYYTRGFQQMGIYAVMIEKMILRDLCRFMF
VYVVFLFGFSTVMSLVRDGEGQGCSMLMVAKWTSEGCRPTGCEGQSRYGTRADMLFK
FTIGMGDLEFTENYDFKAVFIILLLAYVILTYILLLNMLIALMGETVNKIAQESKNIWKL
QRAITILDTEKSFLKCMRKAFRSGKLLQVGYTPDGKDDYRWCFRVDEVNWTTWNTN
VGIINEDPGNCEGVKRTLSFSLRSSRVSARHWKNFALVPLLREASARDRPSAQPEEVH

LRQFSGSLRPEDAEVFKSPPASGEK---

>TRPV1_Ornithorhynchus_anatinus

MKKLGSSDLAELEVTKEPLDDNPGSKPPPPKHHIFSTARSRHRLFTKGDSEESAPMDS
SYEEMEPASCPIIKFSPMVDRRRFMPGRPSPTLIFLRQLSVDSISIGSEKAFKLYDRRRIF
DAVAQGNCRELEDLLVYLLKSTKQLTDVEFKDPETGKTCLLKAMLNLQSGKNETIPLL
LEIAGQTDNLKEFVNAGYTDSYYRGQTALHIAIERRNMYLVTLLVQNGADVHAAAHG
DFFKKTKGRPGFYFGELPLSLAACTNQLGIVKFLLQNPYHSADIDARDSIGNTVLHALV
EVADNTPENTKFVTNMYNEILILGAKIHPTLNLEELTNKKGLTPLALAAKSGKIGYLSS
RGNVLSYQVLAYILRREIQEPECRHLSRKFTEWAYGPVHSSLYDLSCIDTCEKNSVLEVI
AYSSETPNRHDMLLVEPLNRLLQDKWDRFVKRIFYFNFFIYILYIITFTLVACYRPVDL
NQKPPYALGNTALQYLQVFGEFLTLLGGIYFFIRGIQYFLQRQPSLKTLFVDSYSEVLFFI
QSLLLLISVGLYFSGQKMYVAFMVLSLAMGWTNMLYYTRGFQQMGIYAVMIEKMILR
DLCRFMFVYSVFLLGFSTAVVSLIEDGKNSTECTCLWQGKRGNTCKPSSPSYNSLYSTC
LELFKFTIGMGDLEFTDNYDFKFVFIILLLSYVILTYILLLNMLIALMGETVNKVSQESK
NIWKLQRAITILDIEKSFLNCMRKAFRSGKLVQVGYTPDGKDDYRWCFRVDEVNWTH
WNTNLGIINEDPGNSEGIKRTLSFSLRSGRVSGKNWKTFPLVPLLRDGSTRERPTPRP
EEVRLRPFSGSLKPEDAEVQKESLILEE--

>TRPV1_Oryctolagus_cuniculus

MKRWVSLDSGESEDPLPEDTCPDLLDGDSNAKPPPAKPHIFSTAKSRSRLFGKGDSEE
TSPMDCSYEEGELAPCPAITVSSVIIVQRSGDGPTCARQLSQDSVAAAGAEKPLKLYDR
RRIFEAVAQNNCQELESLLCFLQRSKKRLTDSEFKDPETGKTCLLKAMLNLHSGQNDT
IPLLLEIARQTDSLKEFVNASYTDSYYKGQTALHIAIERRNMALVTLLVENGADVQAAA
NGDFFKKTKGRPGFYFGELPLSLAACTNQLAIVKFLLQNSWQPADISARDSVGNTVLH
ALVEVADNTPDNTKFVTSMYNEILILGAKLHPTLKLEELINKKGLTPLALAAGSGKIGV
LAYILQREILEPECRHLSRKFTEWAYGPVHSSLYDLSCIDTCERNSVLEVIAYSSSETPN
RHDMLLVEPLNRLLQDKWDRVVKRIFYFNFFVYCLYMIIFTTAAYYRPVDGLPPYKLR
NLPGDYFRVTGEILSVAGGVYFFFRGIQYFLQRRPSMKALFVDSYSEMLFFVQALFMLA
TVVLYFSHCKEYVATMVFSLALGWINMLYYTRGFQQMGIYAVMIEKMILRDLCRFMF
VYLVFLFGFSTAVVTLIEDGKNSSTSAESTSHRWRGFGCRSSDSSYNSLYSTCLELFKFT
IGMGDLEFTENYDFKAVFIILLLAYVILTYILLLNMLIALMGETVNKIAQESKSIWKLQR
AITILDTEKGFLKCMRKAFRSGKLLQVGYTPDGKDDCRWCFRVDEVNWTTWNTNVG
IINEDPGNCEGVKRTLSFSLRSGRVSGRNWKNFALVPLLRDASTRDRHPXPPEDVHLR
PFVGSLKPGDAELFKDSVAAAEK---

>TRPV1_Otolemur_garnettii

MKKQRSSDLGEPEHLPQQDSCPEPPDGDPDSKPPPAKSYFSTAKSRARLFGKGDSEEV
SSMDGSYEEGEMASCPTITVSPIVIVQRPGDGPTCARQLSQDSVTTGTEKTLKLYDRRR
IFEAVAQNNCQELESLLLFLRKSKKHLTDCEFKDPETGKTCLLKAMLNLHDGQNDTIP
LLLEIARQTDSLKEFVNASYTDSYYKGQTALHIAIERRNMALVTLLVENGADVQAAAN
GDFFKKTKERPGFYFGELPLSLAACTNQLAIVKFLLQNSWQPADISARDSVGNTVLHA
LVEVADNTADNTKFVTTMYNEILILGAKLHPTLKLEELTNKKGMTPLALAAGTGKIGV

LAYILQREIQEPECRHLSRKFTEWAYGPVHSSLYDLSCIDTCEKNSVLEVIAYSSSETPN
RHDMLLVEPLNRLLQDKWDRFVKRIFYFNFFAYCLYMVIFTTAAYYRPVDGLPPFKM
KNTIGDYFRVTGEILSVAGGVYFFFRGIQYFLQRRPSMKALFVDSYSEMLFFIQSLFMLA
TVVLYFSHRKEYVASMVFSLAMGWTNMLYYTRGFQQMGIYAVMIEKMILRDLCRFM
FVYIVFLFGFSTAVVTLIEEGKNNSTSTNSTTHKWRGPGCRQVDSSYNSLYSTCLELFK
FTIGMGDLEFTENYDFKAVFIILLLAYVILTYILLLNMLIALMGETVNKIAQESKNIWKL
QRAITILDTEKSFLKCMRKAFRSGKLLQVGYTPDGKDDYRWCFRVDEVNWTTWNTN
VGIINEDPGNCEGVKRTLSFSLRPGRASGRNWKNFALVPLLRDASTRDRNPAHPEEVH
LRQFSGSLKPEDAEVFKNPTASGEK---

>TRPV1_Myotis_lucifugus

MKKWGSSDSGEPEDLPQEDSHPKPLEGDPNSRPAPAKPSIFPTAKSRSRLFGKCDSEE
ASPMDCSYEEGQPASCPAITVSPVVVVQKTGDGPTCARQSSQDSVPAGTERKLKLYDR
RKIFEAVAQNNCEELESLLLFLQKSKKHLMDSEFKDPETGKTCLLKAMLNLHDGHNS
TIPLLLEIARQTDSLTELVNASYTDSYYKGQTALHIAIERRNMALVTLLVENGADVQAA
ANGDFFKKTKGRPGFYFGELPLSLAACTNQLGIVKFLLQNSWQPADISARDSVGNTVL
HALVEVADNTADNTKFVTSMYNEILILGAKLHPTLKLEELTNKKGLTPLALAASSGKIG
VLAYILQREIQEPECRHLSRKFTEWAYGPVHSSLYDLSCIDTCEKNSVLEVIAYSSSETP
NRHDMLLVEPLNRLLQDKWDRFVKRIFYFNFFVYCLYMIIFTTAAYYRPVDGLPPFKL
RYTVGDYFRVTGEILSVSGGVYFFFRGIQYFLQRRPSLKSLFVDSYSEILFFVQSLFMLGT
VVLYFCRRKEYVASMVFSLAMGWTNMLYYTRGFQQMGIYAVMIEKQVLLACRIATFW
LSFFFFFFKAVVTLIEDGKNDSVAETTSHRWRGPGCRPPDSSYNSLYSTCLELFKFTIG
MGDLEFTENYDFKAVFIILLLAYVILTYILLLNMLIALMGETVNKIAQESKNIWKLQRAI
TILDTEKSFLKCMRKAFRSGKLLQVGYTPDGKDDYRWCFRVDEVNWTTWNTNVGII
NERPNCEASKRTLSFSPALGQKIVSGRHWKNFALVPLLRDASTRERQPAQHEEVHLR
HFAGSLKPEDAEVFKDPAVFGEK---

>TRPV1_Normascus_leucogenys

MKKWSSTDSGAAADPLQQDTCPDPLDGDPNSRPPPAKPQLSMAKSRTRLFGKGDSEE
AFPVDCPHEEGELDSCPTITVSPVIIIQRPGDGPTGARLLSQDSVAASTEKTLRLYDRRSI
FEAVAQNNCQDLESLLLFLQKSKKHLTDNEFKDPETGKTCLLKAMLNLHDGQNDTIP
LLLDIARQTDSLKEFVNASYTDSYYKGQTALHIAIERRNMALVTLLVENGADVQAAAH
GDFFKKTKGRPGFYFGELPLSLAACTNQLGIVKFLLQNSWQPADISARDSVGNTVLHA
LVEVADNTADNTKFVTSMYNEILILGAKLHPTLKLEELTNKKGMTPLALAAGTGKIGV
LAYILQREIQEPECRHLSRKFTEWAYGPVHSSLYDLSCIDTCEKNSVLEVIAYSSSDTPN
RHDMLLVEPLNRLLQDKWDRFVKRIFYFNFLIYCLYMIIFTMAAYYRPVDGLPPFKME
KTEDYFRVTGEILSVLGGVYFFFRGIQYFLQRRPSMKTLFVDSYSEMLFFLQSLFMLAT
VVLYFSHLKQYVASMVFSLALGWTNMLYYTRGFQQMGIYAVMIEKMILRDLCRFMFV
YVVFLFGFSTAVVTLIEDGKNDSLPSESTSHRWRGPGCRPPDSSYNSLYSTCLELFKFTI
GMGDLEFTENYDFKAVFIILLLAYVILTYILLLNMLIALMGETVNKIAQESKNIWKLQR
AITIVPRKKSFVKCMRKAFRSGKLLQVGYTPDGKDDYRWCFRVDEVNWTTWNTNVG
IINEDPGNCEGVKRTLSFSLRSSRVSGRHWKNFALVPLLREASARDRHSAQPEEVHLR
QFSGSLKPEDAEVFKSPAASGEE---

>TRPV1_Mus_musculus

MEKWASLDSDESEPPAQENSCPDPPDRDPNSKPPPAKPHIFATRSRTRLFGKGDSEEA
SPMDCPYEEGGLASCPIITVSSVVTLQRSVDGPTCLRQTSQDSVSTGVETPPRLYDRRSI
FDAVAQSNCQELESLLSFLQKSKKRLTDSEFKDPETGKTCLLKAMLNLHNGQNDTIAL
LLDIARKTDSLKQFVNASYTDSYYKGQTALHIAIERRNMALVTLLVENGADVQAAANG
DFFKKTKGRPGFYFGELPLSLAACTNQLAIVKFLLQNSWQPADISARDSVGNTVLHAL
VEVADNTADNTKFVTNMYNEILILGAKLHPTLKLEELTNKKGLTPLALAASSGKIGVL
AYILQREIHEPECRHLSRKFTEWAYGPVHSSLYDLSCIDTCEKNSVLEVIAYSSSETPNR
HDMLLVEPLNRLLQDKWDRFVKRIFYFNFFVYCLYMIIFTTAAYYRPVEGLPPYKLNN
TVGDYFRVTGEILSVSGGVYFFFRGIQYFLQRRPSLKSLFVDSYSEILFFVQSLFMLVSVV
LYFSHRKEYVASMVFSLAMGWTNMLYYTRGFQQMGIYAVMIEKMILRDLCRFMFVYL
VFLFGFSTAVVTLIEDGKNNSLPVESPPHKCRGSACRPGNSYNSLYSTCLELFKFTIGMG
DLEFTENYDFKAVFIILLLAYVILTYILLLNMLIALMGETVNKIAQESKNIWKLQRAITIL
DTEKSFLKCMRKAFRSGKLLQVGFTPDGKDDFRWCFRVDEVNWTTWNTNVGIINED
PGNCEGVKRTLSFSLRSGRVSGRNWKNFALVPLLRDASTRDRHSTQPEEVQLKHYTG
SLKPEDAEVFKDSMAPGEK--

>TRPV1_Oligodon_lacroixi_fragment

SFYHMEQLDTNPIIKFSFRLERGRCGNHCPKLLSTASIGVCAGKAFKIYDRRSLFDAVA
QGDPSQLDDLLIYLLESLKNLTDAEFKEPDTGKTCLLKAMLNLHNGRNDTIPLLLEIAE
KTENLEEFVNAGYTDGYYKGQTALHIAIERRNMYLVNLLVKNGADVHARAHGEFFQK
IKGKPGFYFGELPLSLAACTNRLNIVKFLLENPYQPANIAAQDSMGSTVLHALVEIADD
TDDNTKFVTKMYNDVLILGASINPTLRLEELTNRRGLTPLTLAAKTGKIQVFAYILRRE
MKEPECRHLSRKFTEWAYGPVHSSLYDLSSIDTCEKNSVLEIIAYSSETPNRHEMLLVE
PLNQLLQDKWDRFVKHLFYFNFFIYTAHILILTVAAYYRPTKKEGMPPFTFHHSSGEY
FRVTGEILRVLGGAYFFFRGIKYFQQRRPSLKAIFTECYSELLFFAHSVLLLGSAVLYFSK
QEVYVVLMVFALALGWSNLIYYTRGFQQMGIYSVMLEKMMLRDLGRFIMVYMLFHLG
FSTAVVTLIEEDNEGRPEIPPQQDSCPCLVRARPSYNNLYTTCLELFKFTIGMGDLEFT
ENYRFKSVFIILLLVYVVLTYILLLNTLIALMGETVNKIAQESKSIWKLQRTITILNIEKSY
WNCIINSFRSGKRVLVGTTPDGKEDYRWCFRVDEVNWSTWNTNLS------------------
----

>TRPV1_Ovophis_monticola_fragment

SFYHMEQLASNPIIKFSLSLERGRCGDHCPKLLSTASIGVCAGKAFKIYDRRCLFDAVAQ
GDTSQLDDLLIFLLESLKILTDAEFKEPDTGKTCLLKAMLNLHNGRNDTIPLLLEIAEK
TENLKEFVNTGYTDSYYRGQTALHIAIERRNMYLVNLLVKNGADVHARAHGEFFQKI
KGKPGFYFGELPLSLAACTNQLNIVKYLLENPYQQANIAEQDSMGNTVLHALVEIADN
TADNTKFVTRMYNDMLILGARINPTLRLEEVANRRGLTPLTLAAKMGKIQVFAYILRR
EMKEPECRHLSRKFTEWAYGPVHSSLYDLSSIDTCEKNSVLEIIAYSSETPNRHEMLLV
EPLNQLLQDKWDRFVKHLFYFNFFTYTVHILILTVAAYYRPTKKEGTPPFTFRHTRGE
YFRVTGEILSVLGGAYFFFRGIKYFQQRRPPLKAIFTECYSELLFFVHSLLLLGSAVLYFS
RQEVYVVLMVFALALGWSNLLYYTRGFQQMGIYSVMLEKMMLRDLGRFIMVYLLFHL
GFSTAVVTLIEEDNEGRPETAQPLDVCPCPVRARPSYNNLYTTCLELFKFTIGMGDLEF

TENYHFKSIFIILLLIYVVLTYILLLNMLIALMGETVNKIAQESKSIWKLQRTITILNIENS
YWNCIINSFRSGKRVLVGTTPDGKDDYRWCFRVDEVNWSTWNTNLS-----------------
----

>TRPV1_Plagiopholis_blakewayi_fragment

SFYHMEQLDSNPIIKFSFRLERGRCGNHCPKLLSTASIGVCAGKAFKIYDRRSLFDAVAQ
GDPSQLDDLLIYLLESLKTLTDAEFKEPDTGKTCLLKAMLNLHNGRNDTIPLLLEIAEK
TESLQEFVNAGYTDGYYKGQTALHIAIEGRNMYLVNLLVKNGADVHARAHGEFFQKIE
GKPGFYFGELPLSLAACTNQLNIVKFLLENPYQPANIAEQDSMGNTILHALVEIADDTD
DNTKFVTKMYNDVLILGASTNPTLRLEELTNRRGLTPLTLAAKTGKIQVFAYILRREM
KEPECRHLSRKFTEWAYGPVHSSLYDLSSIDTCEKNSVLEIIAYSSETPNRHEMLLVEP
LNQLLQDKWDRFVKHLFYFNFFIYTVHILILTVAAYYRPTKKEGTPPFTFHHRRDEYF
RVTGEILSVSGGAYFFFRGIKYFQQRRPSLKAIFTECYSELLFFVHSVLLLGSAVLYFSKQ
EVYVVLMVFALALGWSNLIYYTRGFQQMGIYSVMLEKMMLRDLGRFIMVYLLFHLGF
STAVVTLIEEDNEGRPEIPQPQACPCPVKARPSYNNLYTTCLELFKFTIGMGDLEFTEN
YRFKSVFIILLLIYVVLTYILLLNMLIALMGETVNKIAQESKSIWKLQRTITILNIENSYW
NCIINSFRSGKRVLVGTTPDGKEDYRWCFRVDEVNWSTWNTNLS---------------------
----

>TRPV1_Protobothrops_jerdonii_fragment

SFYHMEQLASNPIIKFSLSLERGRCGDHCPKLLSTASIGVCAGKAFKLYDRRSLFDAVA
QGDPSQLDDLLIFLLESLKILTDAEFKEPDTGKTCLLKAMLNLHNGRNDTIPVLLEIAE
KTENLKEFVNTGYTDSYYRGQTALHIAIERRNMYLVNLLVKNGADVHARAHGEFFQK
IKGKPGFYFGELPLSLAACTNQLNIVKYLLENPYQPANIAEQDSMGNTVLHALVEIADN
TADNTKFVTRMYNDMLILGARINPTLRLEEVANRRGLTPLTLAAKMGKIQVFAYILRR
EMKESECRHLSRKFTEWAYGPVHSSLYDLSSIDTCEKNSVLEIIAYSSETPDRHEMLLV
EPLNQLLQDKWDRFVKHLFYFNFFTYTVHILILTVAAYYRPTKKEGTPPFTFRHTRGE
YFRVTGEILSVLGGAYFFFRGIKYFQQRRPSLKAIFTECYSELLFFVHSLLLLGSAVLYFS
RQEVYVVLMVFALALGWSNLLYYTRGFQQMGIYSVMLEKMMLRDLGRFIMVYLLFHL
GFSTAVVTLIEEDNEGRPETAQPAGVCPCPVRARPSYNNLYTTCLELFKFTIGMGDLEF
TENYHFKSIFIILLLIYVVLTYILLPNMLIALMGETVNKIAQESKSIWKLQRTITILNIENS
YWNCIVNSFRSGKRVLVGTTPDGKDDYRWCFRVDEVNWSTWNTNLS-----------------
----

>TRPV1_Pseudoxenodon_macrops_fragment

SFYHMEQLDSNPIIKFSFRLERGRCGNHCPKLLSTASIGVCAGKAFKIYDRRSLFDAVAQ
GDPSQLDDLLIYLLESLKTLTDAEFKEPDTGKTCLLKAMLNLHNGRNDTIPLLLEIAEK
TESLQEFVNAGYTDGYYKGQTALHIAIERRNMYLVNLLVKNGADVHARAHGEFFQKIE
GKPGFYFGELPLSLAACTNQLNIVKFLLENPYQPANIAEQDSMGNTILHALVEIADDTD
DNTKFVTKMYNDVLILGASINPTLRLEELTNRRGLTPLTLAAKTGKIQVFAYILRREMK

EPECRHLSRKFTEWAYGPVHSSLYDLSSIDTCEKNSVLEIIAYSSETPNRHEMLLVEPL
NQLLQDKWDRFVKHLFYFNFFIYTVHTLILTVAAYYRPTKKEGTPPFTFHHRRGEYFR
VTGEILSVLGGAYFFFRGIKYFQQRRPSLKAIFTECYSELLFFVHSVLLLGSAVLYFSKQE
VYVVLMVFALALGWSNLIYYTRGFQQMGIYSVMLEKMMLRDLGRFIMVYLLFHLGFS
TAVVTLIEEDNEGRPETPPPQVCPCPVKARPSYNNLYTTCLELFKFTIGMGDLEFTENY
RFKSVFIILLLIYVVLTYILLLNMLIALMGETVNKIAQESKSIWKLQRTITILNIENSYWN
CIINSFRSGKRVLVGTTPDGKEDYRWCFRVDEVNWSTWNTNLS-----------------------
----

>TRPV1_Xenopus_laevis

MKKMGSSTDIDETEETCASIETDESHSDDTNRSAQENRKKLKFCQAKYSIFSSPKPKGR
RFGKTETDRDMAPMDSVYQIESKVISPAIKFHRNLERGKLCNQLVRQSTSLESTSSCKD
RTFKLYDQRRIFDAAAYGDCEELDDLLVYLLRTHKRLTNEEFKEKETGKTCLLKAMLN
LDKGMNHTILLFLEIAEKTDNLKEFINSAYRDNYYRGQTALHIAIERRNMDLVQLLLQ
HGADVHARADGEFFRKAKGKAGFYFGELPLSLAACTNQTAIVRYLLQNQHSPANIAAR
DSFGNTVLHALVDIADNTQENTAFVTKMYNEILVLGAQIKPSLKIEEIANKKGLTPLSL
AAKTGKIGVFAYILRREIKNLECRHLSRKFTEWAYGPVHSSLYDLSGVDTYEKNSVLEII
AYSSETPNRHDMLLVEPLNKLLQDKWDHFVKRIFYFNFFAYIIYVIIFTIAAYYRPVDGS
PPFPVQYGSYLRTSGELITVIGGIYFFFRAIQYFTQRRPSLKALLADSYCEFLFFSQSVFLL
LSTVLYFCGRNEYVAFLVICLAMSWANVLYYTRGFQLMGIYSVMIEKLILSDMVRFLFV
YLLFLFGFAAALVTLIEDGEGRTDVNNTCGRRCCKPEPASYNNLYYTCQELFKFAIGMG
DLEFTDNYKYKPVFIFLLITYVILTYILLLNMLIALMGETVSKVAQESKSIWKLQRAITIL
DIEKSFLNSFRDTFRSGKSVLVGITPDGKEDYRWCFRVDEVNWNKWNSNLGIIKEDPG
NCHGFKSTLSASFRPRGRRWRSLVPHIKEINLGNENETVPEEVPLQIQPALSVQTVKEE
DQEVTSKAE--

>TRPV1_Xenopus_tropicalis

MKKMGSSTDIDETEETCASVETDESHSDDTNRSTQENRKKLKFCQAKYSIFSSPKPKG
RRFGKAETDRNIAPMDSVYQIESKVMSPTIKFQRNLEKGKLCNQLVRQSSSLESTTSCK
DRTFKLYDQRRIFDAAAYGDCDELDDLLVYLLRTHKKLTNEEFKEKETGKTCLLKAM
LNLENGMNSTIPLFLEIAEKTDNIKEFINSAYRDNYYRGQTALHIAIERRNMDLVELLL
QHGADVHARADGEFFRKAKGKAGFYFGELPLSLAACTNQTAMVQYLLQNQYSPANM
AAKDTFGNTVLHALVDIADNTQENTTFVTKMYNEILVLGAQIRPSLKLEEIMNKKGLT
PLSLAAKTGKIGVFAYILRREIKNFECRHLSRKFTEWAYGPVHSSLYDLSGVDTYEKNS
VLEIIAYSSETPNRHEMLLVEPLNKLLQDKWDRFVKRIFYFNFLAYITYVIIFTVAAYYR
PVDGSPPFPVQPNSYLRTCGELITVIGGIYFFFRGIQYFTQRRPSLKALIADSYYEFLFFA
QSIFLLLSTMLYFCGRNEYVAFLVICLAMSWANVLYYTRGFQLMGIYSVMIEKLILSDM
VRFMFVYLLFLFGFAAALVTLIEDGEGRTDLNSTCGRRCCKPEPASYNNLYYTCQELFK
FAIGMGDLEFTDNYKYKPVFIFLLITYVILTYILLLNMLIALMGETVSKVAQESKSIWKL
QRAITILDIEKSFLNSFRDTFRSGKSVLVGFTPDGKEDYRWCFRVDEVNWNKWNSNL
GIIKEDPGNCHGLKSTLSASFRPRGKRWKSLVPHVKETNVKIDNETVPEEIPLQQKPTL
ADQTVPEEDQEVTSKAE---

>TRPV1_Zebra_finch

RLLSTDSITGCSEKAFRFYDRRRIFDAVAQGNTKDLSDLLLYLNRTFKHLTDEEFKFTE
PETGKTCLLKAMLNLHDGKNDTIPLLLDIARKTGTLKEFVNAEYTDNYYKGQTALHIA
IERRNMYLVKLLVQNGADVHARACGEFFRKIKGKSGFYFGELPLSLAACTNQLCIVKFL
LENPYQAANITAEDSMGNMVLHTLVEIADNTKDNTKFVTKMYNNILILGAKINPILKL
EELTNKKGLTPLTLAAKTGKIGIFAYILRREIKDPECRHLSRKFTEWAYGPVHSSLYDLS
CIDTCEKNSVLEILAYSSETPNRHEMLLVEPLNRLLQDKWDRFVKHLFYFNFFVYTMH
ITILTAAAYYRPVQKNEKPPFTFGYSPGEYFRVAGEILSVLGGLYFFFRGVRYVHYSCQH
WGIFRVTGEILSVLGGLYFFFRGIQYFVQRRPSLRTLIVDGYSEVLFFVHSLLLLSSVVLY
FCGQELYVASMVFSLALGWTNMLYYTRGFQQMGIYSVMIAKMILRDLCRFMFVYLVF
LLGFSTAVVTLIEDDNEGQDTNISDYARCCQVKRGRTSYNSLYYTCLELFKFTIGMGDL
EFTENYRFKSVFVILLVLYVILTYILLLNMLIALMGETVNKIAQESKSIWKLQRAITILDI
ENSYLNCLRHSFRSGKQVLVGITPDGQDDYRWCFRVDEVNWSTWNTNLGIINEDPGY
SGDLRRNLSFSIKPGRVSGKNWKTLVPLLKDGEKRREEPHKPPEEVKLKP--------------
----

>TRPV1_Bungarus_multicinctus_fragment

SFYHIEQLDSNPIIKFSFRLEKGHCGNHCPKLLSTASIGVCAGKAFKIYDRRSLFDAVAQ
GDPSQLDDLLIYLLESLKNLTDAEFKEPDTGKTCLLKAMLNLHNGRNDTIPFLLEIAAK
TENLQEFVNAGYTDSYYKGQTALHIAIERRNMYLVNLLVKNGADVHARAHGEFFQKI
KGKPGFYFGELPLSLAACTNQLNIVKFLLENPYQPANIAAQDSMGNTVLHALVGIADD
TDDNTKFVTKMYNDVLILGARINPTLRLEEIANRRGLTPLTLAAKTGKIQVFAYILRRE
MKEPECRHLSRKFTEWAYGPVHSSLYDLSSIDTCEKNSVLEIIAYSSETPNRHEMLLVE
PLNQLLQDKWDRFVKHLFYFNFFIYTAHILILTVAAYYRPTKREGTPPFIFHHTRGEYF
RVTGEILSVLGGAYFFFRGIKYFQQRRPSLKATFTECYSELLFFFHSVLLLGAAVLYVSR
QEVYYVVLMVFALALGWSNLIYYTRGFQQMGIYSVMLEKMMLRDLGRFIMVYLLFHLG
FSTAVVTLIEEDNEGRAETTQQQLEACPCPVKPRPSYNNLYTTCLELFKFTIGMGDLEF
TENYRFKSVFIILLLVYVVLTYILLLNMLIALMGETVNKIAQESKSIWKLQRTITILNIEN
SYWNCIVNSFRSGRRVLVGITPDGKEDYRWCFRVDEVNWSTWNTNLS-----------------
----

>TRPV1_Amphiesma_sp_fragment

SFYHMEQLDSNPIIKFSFRLERGRCGNHCPKLLSTASNGVCAAKAFKIYDRRSLFDAVA
QGDPSQLDDLLIYLLESLKNLTDAEFKEPDTGKTCLLKAMLNLHNGRNDTIPLLLEIAE
KTENLKEFVNAGYTDGYYKGQTALHIAIERRNMYLVNLLVKNGADVHARAHGEFFQK
IKGKPGFYFGELPLSLAACTNQLNIVKFLLENPYQPANIAEQDSIGNTVLHALVEIADDT
DENTKFVTKMYNDVLILGASINPTLRLEELANRRGLTPLTLAAKTGKIQVFAYILRREM
KEPECRHLSRKFTEWAYGPVHSSLYDLSSIDTCEKNSVLEIIAYSSETPNRHEMLLVEP
LNQLLQDKWDRFVKHLFYFNFFIYTAHILILTVAAYYRPTKKEGTPPFAFHHTSSEYFR
VTGEILSVLGGAYFFFRGIKYFQQRHPSLKAIFTECYSELLFFVHSVLLLGSAVLYFSKQE
VYYVVLMVFALALGWSNLIYYTRGFQQMGIYSVMLEKMMLRDLGRFIMVYLLFHLGFS
TAVVTLIEEEDGRPETPPPPEGCPCPVKARPSYNNLYTTCLELFKFTIGMGDLEFTENY
RFKSVFIILLLFYVVLTYILLLNMLIALMGETVNKIAQESKSIWKLQRTITILNIENSYWN

CIINSFRSGKCVLVGTTPDGKEDYRWCFRVDEVNWSTWNTNLS-------------------------
----

>TRPV1_Carollia_brevicauda

MKKRGSSDSGESEDLPQEDPLPDPLDGDPHSRPAPAKPNIPTAKSRSRLFGKGDSEEA
SSLDCSYKEGQPASCPAITVSPVLMVQKTEDGPTCTWQLCQDSVPAGTKKSLKLYDRR
KIFEAVAQNNCEELESLLLFLQRSKKHLMDSEFKDPETGKTCLLKAMLNLHNGQNNTI
PLLLEIARQTDSLKELVNASYIDSYYKGQTALHIAIERRNMALVTLLVENGADVQAAAN
GDFFKKTKGRPGFYFGELPLSLAACTNQLGIVKFLLQNSWQPADISARDSVGNTVLHA
LVEVADNTADNTKFVTSMYNEILILGAKLHPTLKLEELINKKGLTPLALAASSGKIGVL
AYILQREIHEPECRHLSRKFTEWAYGPVHSSLYDLSCIDTCEKNSVLEVIAYSSNETPNR
HDMLLVEPLNRLLQDKWDRFVKRIFYFNFFVYCLYMIIFTTVAYYRPVEGLPPYKVKH
TVGDYFRVTGEILSVLGGVYFFFRGIQYFLQRQPSLKSLFVDSYSETLFFVQSLFMLGTV
VLYFCQCKEYVASMVFSLAMGWTNMLYYTRGFQQMGIYAVMIEKMILRDLCRFMFVY
LIFLFGFSTAVVTLIEDGKNDSVSTETTPHRWRGPCYRSPDSSYNSLYSTCLELFKFTIG
MGDLEFTENYDFKAVFIILLLAYVILTYILLLNMLIALMGETVNKIAQESKNIWKLQRAI
TILDTEKSFLKCMRKAFRSGKLLQVGYTPDGKDDYRWCFRVDEVNWTTWNTNVGII
NEDPGNCEGIKRTLSFSLRSSRVAGRNWKNFALVPLLRDASTRERQPAQPEEVHLKHF
SGSLKPEDAEVFKDPAALG---

>TRPV1_Crotalus_atrox

MRKLGSSDLEESESTDDHTDGEDAAAGTPKGTKHGIFARRARFGIGDGDKDMAPMDS
FYHMEQLASNPIIKFSLSLERGRCGNHCPKLLSTASIGVCAGKAFKIYDRRTLFDAVAQ
GDTSQLDDLLIFLLESVKNLTDAEFKEPDTGKTCLLKAMLNLHNGRNDTIPLLLEIAEK
TENLKEFVNTGYTDSYYRGQTALHIAIERRNMYLVNLLVKNGADVHARAHGEFFQKI
KGKPGFYFGELPLSLAACTNQLNIVKYLLENPYQPANIAEQDSMGNTVLHALVEIADN
TVDNTKFVTRMYNDMLILGARINPALRLEEVTNRRGLTPLTLAAKMGKIQVFAYILRR
EMKEPECRHLSRKFTEWAYGPVHSSLYDLSSIDTCEKNSVLEIIAYSSETPNRHEMLLV
EPLNQLLQDKWDRFVKHLFYFNFFTYTVHILILTVAAYYRPTKKEGTPPFTFRHSRGE
YCRVTGEILTVLGGAYFFFRGIKYFQQRRPSLKAIFTECYSELLFFVHSLLLLGSAVLYFS
GQEVYVVLMVFALALGWSNLLYYTRGFQQMGIYSVMLEKMMLRDLGRFIMVYLLFHL
GFSTAVVTLIEEDNEGRPETAQPLDVCPCPVRARPSYNNLYTTCLELFKFTIGMGDLEF
TENYHFKSIFIILLLIYVVLTYILLLNMLIALMGETVNKIAQESKSIWKLQRTITILNIENS
YWNCIINSFRSGKRVLVGTTPDGKDDYRWCFRVDEVNWSTWNTNLSIINEDPGGHTE
ELKRNLSFSFKSGRVSGKNWKTLVPILRDGKREGSLKPISEDYADSDEQDVRKKSLPN
PVL---

>TRPV1_Daboia_siamensis_fragment

SFYHMEQLASNPIIKFSLSLERGRCGNHCPKLLSTASIGVCAGKAFKIYDRRSLFDAVAQ
GDPSQLDDLLIFLLESLKNLTDAEFKEPDTGKTCLLKAMLNLHNGRNDTIPLLLEIAEK
TENLTEFVNAGYTDSYYRGQTALHIAIERRNMYLVNLLVKNGADVHARAHGEFFQKI
KGKPGFYFGELPLSLAACTNQLNIVKYLLENPYQAANIAEQDSMGNTVLHALVEIADN

TADNTKFVTRMYNDMLILGARINPTLRLEEVANRRGLTPLTLAAKMGKIQVFAYILRR
EMKEPECRHLSRKFTEWAYGPVHSSLYDLSSIDTCEKNSVLEIIAYSSETPNRHEMLLV
EPLNQLLQDKWDRFVKHLFYFNFFTYAVHILILTVAAYYRPTKKEGTPPFTFRHTRGE
YFRVTGEILSVLGGAYFFFRGIKYFQQRRPSLKAIFTECYSELLFFVHSVLLLGSAVLYFS
RQEVYVVLMVFALALGWSNLLYYTRGFQQMGIYSVMLEKMMLRDLGRFIMVYLLFHL
GFSTAVVTLIEEDNEGRPEMAQPLDVCPCPVKARPSYNNLYTTCLELFKFTIGMGDLE
FTENYHFKSIFIILLLIYVVLTYILLLNMLIALMGETVNKIAQESKSIWRLQRTITILNIEN
SYWNCIINSFRSGKRVLVGTTPDGKDDYRWCFRVDEVNWSTWNTNLS----------------
----

>TRPV1_Danio_rerio

MDSSVSSFSLETDDQTEEERTKAKQMKKVSKDKRPMDSNYVDDVVEPSSTIKFNLHF
DRGIRNLKEEPAQQDNDRFTIKRLFEAVSSGDVSKMQGLHEYLHKNMKRLTDSQYKS
NGKTALLKALLNLRQGENDTIEQLLDIAEKMGDLKNFINAAYTDSYYKGQTALHVAIE
RRSMKFVQMLVKKGADVHAKACGKFFQPNQKMCFYFGELPLSLAACTNQQDIVDFL
MENPHQAVDVRERDCHGNTVLHALVSVADNSPENTEFVIAMYDHILIKADQLHPKTK
LEEIENNEGLTPITLAAKKGKLGLFKHIVQRELMGCRHLSRKITEWAYGPVCSSLYDLS
SLDTYEKNSALEIVVYGSEIPNRLEMLQIEPLNRLIEEKWDQFAHRMFLFNFIVYVIYLF
IFTASAFYHEEGKDYANQPPYLYAKSREGYLLLTGHIISITGAFYFFIRGLIDMVRKRPRF
QSLIIDGYTDQLFFVQGLLFLASVVLYCYGQYEYLAFLVLCLALSWINLLYFSRGSKNLGI
YNVMIQKMVLGEIRRFLVVYMVFLIGFSAALVTLLDQESIDSGSTRDFRLSEDIPSLNPT
PDSSNPQSRMTHHQPTTARDGRGRFGLTTDNQYEVCKKPSYKNIYFTTLELFKFTIGM
GDLEFTDHYKYKEVFYVLLIVYIVMTYILMLNMLIALMNQSVEMMSVESTSIWKLQRA
ITTLDMEWILPKCLQGKLRSGEEKDLGGGQEPDRRWCFSVEEVNWTQWNRNMGIIIN
EDPGKCTQDPSPANVQREPSRGVLQTFSRRRRTQRAQTREGHELSPLAEASSSV-------
----

>TRPV1_Eryx_tataricus_fragment

SFYHMEHLGSSPIIKFSLSLERGRCGNHCPNLLSTASIGVRAGKAFKIYDRRSLFDAVAQ
GDPSMLDDLLVYLLESLKNLTDEEFKEPDTGKTCLLKAMLNLHHGRNDTIPLLLEIAE
KTQNLKEFVNAGYTDSYYKGQTALHIAIERRNLYLVNLLVKNGADVHARAHGEFFQKI
KGKPGFYFGELPLSLAACTNQLNIVKYLLENPYQPAHIAEQDSMGNTILHALVEIADDT
EDNTKFVTKMYNDVLILGARINPTLRLEEIANRRGLTPLTLAAKTGKIQVFAYILRREM
KEPECRHLSRKFTEWAYGPVHSSLYDLSSIDTCEKNSVLEIIAYSSETPNRHEMLLVEP
LNQLLQDKWDRFVKHLFYLNFFIYTAHILILTVAAYYRPTKKQGTPPFTFHHTSSEYLR
STGELLSVTGGAYFFFRGIKYFQQRRPSLKAIFTESYSELLFFVHSVLLLGSVVLYFSRQE
TYVVLMVFALALGWANLLYYTRGFQQMGIYSVMLEKMMLRDLCRFIMVYLLFHLGFS
TAVVTLIEEDNEGRPEISQTPNVCPCPVKPRPSYNNLYTTCLELFKFTIGMGDLEFTEN
YHFKSVFIILLLIYVVLTYILLLNMLIALMGETVNKIAQESKSIWKLQRAITILNIENSYW
NCIVNSFRSGKRVLVGTTLDGKEDYRWCFRVDEVNWSTWNTNLS---------------------
----

>TRPV1_Meleagris_gallopavo

MSSILEKMKKFGSSDIEESEVTDEHTDGEDSALEAPDNLQGTFSSKVQPSRSNIFARRG
RFVMGDSDKDMAPMDSFYQMDHLMAPSVIKFHANMERGKLHKLLSTDSITGCSEKA
FKFYDRRRIFDAVAQGNTKDLDDLLLYLNRTLKHLTDDEFKEPETGKTCLLKAMLNL
HDGKNDTIPLLLDIAKKTGTLKEFVNAEYTDNYYKGQTALHIAIERRNMYLVKLLVQN
GADVHARACGEFFRKIKGKPGFYFGELPLSLAACTNQLCIVKFLLENPYQAADIAAEDS
MGNMVLHTLVEIADNTKDNTKFVTKMYNNILILGAKINPILKLEERTNKKGLTPLTLA
AKTGKIGIFAYILRREIKDPECRHLSRKFTEWAYGPVHSSLYDLSCIDTCEKNSVLEIIAY
SSETPNRHEMLLVEPLNRLLQDKWDRFVKHLFYFNFFVYAIHISILTTAAYYRPVQKG
ENPPFTFGHSTGEYFRVTGEILSVLGGLYFFFRGIQYFVQRRPSLKTLIVDSYSEVLFFVH
SLLLLSSVVLYFCGQELYVASMVFSLALGWANMLYYTRGFQQMGIYSVMIAKMILRDL
CRFMFVYLVFLLGFSTAVVTLIEDDNEGQDTNSSECARCSHTKRGRTSYNSLYYTCLEL
FKFTIGMGDLEFTENYRFKSVFVILLVLYVILTYILLLNMLIALMGETVSKIAQESKSIW
KLQRAITILDIENSYLNCLRRSFRSGKQVLVGITPDGQDDYRWCFRVDEVNWSTWNT
NLGIINEDPGCSGDLKRNPSYSIKPGRVSGKNWKTLVPLLRDGSRREETQKLPEEIKLK
PILEPYYEPEDCETLKESLPKSV--

>TRPV2_Rattus_norvegicus

MTSASSPPAFRLETSDGDEEGNAEVNKGKQEPPPMESPFQREDRNSSPQIKVNLNFIK
RPPKNTSAPSQQEPDRFDRDRLFSVVSRGVPEELTGLLEYLRWNSKYLTDSAYTEGST
GKTCLMKAVLNLQDGVNACIMPLLQIDKDSGNPKLLVNAQCTDEFYQGHSALHIAIEK
RSLQCVKLLVENGADVHLRACGRFFQKHQGTCFYFGELPLSLAACTKQWDVVTYLLE
NPHQPASLEATDSLGNTVLHALVMIADNSPENSALVIHMYDGLLQMGARLCPTVQLE
EISNHQGLTPLKLAAKEGKIEIFRHILQREFSGPYQPLSRKFTEWCYGPVRVSLYDLSSV
DSWEKNSVLEIIAFHCKSPNRHRMVVLEPLNKLLQEKWDRLVSRFFFNFACYLVYMFI
FTVVAYHQPSLDQPAIPSSKATFGESMLLLGHILILLGGIYLLLGQLWYFWRRRLFIWIS
FMDSYFEILFLLQALLTVLSQVLRFMETEWYLPLLVLSLVLGWLNLLYYTRGFQHTGIY
SVMIQKVILRDLLRFLLVYLVFLFGFAVALVSLSREARSPKAPEDNNSTVTEQPTVGQE
EEPAPYRSILDASLELFKFTIGMGELAFQEQLRFRGVVLLLLLAYVLLTYVLLLNMLIAL
MSETVNHVADNSWSIWKLQKAISVLEMENGYWWCRRKKHREGRLLKVGTRGDGTP
DERWCFRVEEVNWAAWEKTLPTLSEDPSGPGITGNKKNPTSKPGKNSASEEDHLPLQ
VLQSP---

>TRPV2_Ailuropoda_melanoleuca

MASPSSPLAFRLQISDGGQEDGGEVDKGGRGDGPPPMESPFQGEDRNSSPQIKVNLNY
RKRAGVSQPDPNRFDRDRLFSVVARGVPEDLAGLPEYLSRTSKYLTDSEYTEGSTGKT
CLMKAVLNLRDGANACILPLLQIDRDSGNPQPLVNAQCTDEYYRGHSALHIAIEKRSLQ
CVKLLVENGANVHARACGHFFQKKSQGTCFYFGELPLSLAACTKQWDVVTYLLENPH
QPASLQAADSLGNTVLHALVMIADNSAENSALVIRMYDGLLRTGARLCPTVQLEDIPN
LQGLTPLKLAAKEGKIEIFRHILQREFSGPCQSLSRKFTEWCYGPVRVSLYDLASVDSW
EENSVLEIIAFHCRSPHRHRMVVLEPLNKLLQAKWDLLIPKFFFNFLCYLTYMLLFTAV
AYHQPALEKPFFPPKVTAGDSMLLLGHILILLGGVYLLVGQLWYFWRRRLFIWISFVDS
YFELLFLAQALLMVLSQALCFLAVEWYLPLLVSSLVLGWLNLLYYTRGLQHTGIYSVMI

QKVILRDLLRFLLVYLVFLFGFAVALVSLSREARAPGVPTGPNVTEAVQPGAGQTDEEG
SAPPYGSILDASLELFKFTIGMGELAFQDQLRFRGVVLLLLLAYVLLTYILLLNMLIALM
SETVNSVATDSWSIWKLQKAISVLEMENGYWWCRRKKQPAGVKLTIGTRPDGSPDER
WCFRVEEVNWAAWEQTLPTVCEEPSGPAVPGTLKNPAPASQLEEDTASEEDQLPLQL
LKSQ---

>TRPV2_Bos_taurus

MTTPSSSPAFRLETSDGGDEDGAQGDTGNGGRSSEPPPMESRFQSEDRNSSPQIRVNL
NFRRAAGASQPDPNRFGRDRLFSAVARGAPEDLAGLPEYLRRTSKYLTDSEYREGSTG
KTCLMKALLNLRDGANACIEPLLQIDRDSGNPHPLVNAQCMDEYYRGHSALHIAIEKR
SLVCVKLLVENGADVHARACGQFFQKRREETCFYFGELPLSLAACTQQWDVVTYLLK
NTHQPASLQATDSLGNTVLHALVMIADNSPENSMLVTHMYDRLLQVGGLQLEDMTN
LQGLTPLKLAAKEGKIEIFRHILQRELPEPYQSLSRKFTEWSYGPVRVSLYDLASVDSW
EENSVLEIIAFHSRSPLRHRMVVLEPLNKLLKAKSNLLIPRFLFNFLCYLTYMLIFTAVT
YYQPALEKDFLPLEVTAGNTMLLLGHVLLLLGGVNLLMGQLWYFWRRRLFIWISFMD
SYFEILFLVQALLTVLSQVLRFLAVKGYLPLLVCSLVLGWLNLLYYMRGFQYTGIYSVMI
QKVILRDLLRFLGVYLVFLFGFAVALVSLSREPQDTGAPAGSNTTEVAGKEDKEAPYRG
ILDTSLELFKFTIGMGELAFQDQLHFRGVVLLLLLAYVLLTYVLLLNMLIALMSETVNS
VATDSWSIWKLQKAISVLEMEKGYWWCRRKQQRAGVKLTVGSRPDGSPDERWCFR
VEEVNWAAWEQTLPTVFEEPSGRGGPGAITSPALASQSSQDSAVEEDHVPLQPLESH-

>TRPV2_Callithrix_jaccus

MTSPSSPPAFRLETLDRDQEDGSETDKEKLEFGRGPPPMESQFQSEDRNVSPQIKVKL
NYRKGTGGSQQDENRFDRDWLFNAVSRGVPEDLAGLPEYLRKTSKYLTDSDYTEGST
GKTCLMKAVLNLKDGVNACILPLLQIDQHSGNPQPLVSAQCKDDYYRGHSALHIAIEK
RSLQCVKLLVENGADVHARACGRFFQKGQGTCFYFGELPLSLAACTKQWDVVSYLLE
NPHQPASLQATDSQGNTVLHALVMISDNSAENITLVTSMYDGLLQAGARLCPTVQLE
DIPNLQGLTPLKLAAKEGKIRIFSHILQREFSGPSQRLSRKFTEWCYGPVRVSLYDLASV
DSWEENSVLEIIAFHCKSPHRHRMVVLEPLNKLLQEKWDLLIPRFFLNFLCNLIYMLIF
TTVAYHQPALKKQADSHLKAEVGNSMLLAGHIIILLGGLYILVGQLWYFWRRHLFVWI
WFIDSYFEILFLLQALLTVLSQVLCFLAIEWYLPLLVSALVLGWLNLLYYTRGFQHTGIY
SVMIQKVILRDLLRFLLVYLVFLFGFAVALVSLSQEAWRPEAPTGSNATESLQPVEGQK
DKENTSPYGNILEASLELFKFTIGMGELAFEEQLHFRGMVLLLLLAYVLLTYILLLNMLI
ALMNETVNSVATDSWSIWKLQKAISVLEMENGNWWYRKKQRRAGVMLTVGTKADD
SPDERWCFRVEEVNWASWEQTLPTLCEDPSGADVPRTLKNPVLASPPKDEDGAAEE
DHVSLQLLQSS--

>TRPV2_Canis_familiaris

MASPSSSPTFRLETSDGGQEDGSEVDKRKAGPGAGPPPMESPFQGEDRKCSPQIKVNL
NFRKGAGVSQPDPNRFDRDRLFSVVARGVPEDLAGLPEYLSRTSKYLTDSEYTEGSTG
KTCLMKAVLNLRDGANACILPLLQIDRNSGNPQPLVNAQCTDEYYRGHSALHIAIEKR
SLQCVKLLVENGANVHAQACGQFFQKKSQGTCFYFGELPLSLAACTKQWDVVTYLLE
NPHQPASLQAADSLGNTALHALVMIADNSAENSALVIRMYDALLRAGARLCPKVQLE
DIPNLQGLTPLKLAAKEGKIEIFRHILQREFSGPCQSLSRKFTEWCYGPVRVSLYDLASV
DSWEENSVLEIIAFHCRSPHRHRMVVLEPLNKLLQAKWDLLIPRFFFNFLCYLTYMFI
FTAVAYHQPALGKAFLPPTVTTGDSMLLLGHILILLGGVYLLVGQLWYFWRRRLFIWI
SFVDSYFELLFLVQALLTVLSQGLCFLAVEWYLPLLVSSLVLGWLNLLYYTRGLQHTGI
YSVMIQKVILRDLLRFLLVYLVFLFGFAVALVSLSREARDSGVPTGPNVTEAAQSGAGQ
GDEEGSSSPYGGILDASLELFKFTIGMGELAFQDQLRFRGVVLLLLLAYVLLTYILLLNM
LIALMSETVNSVASDSWSIWKLQKAISVLEMENGYWWCRRKKQRAGVKLTVGTRPD
GSSDERWCFRVEEVNWAAWEQTLPTVCEEPSGPAVPGSIRNPVLASQPEEDTASEED
QLPLQLLKSH--

>TRPV2_Cricetulus_griseus

MTSPSSPPAFRLETSDGDQEDSAEVDKGRNEPPPMESPFQGEDRNFSPQIKVNLNYRK
GRGASQQDPNRFDRDRLFSVVSRGVPEELAGLREYLCRTSKYLTDSAYTEGSTGKTCL
MKAVLNLQDGVNACILPLLQIDKDSGNPQPLVNAQCTDEFYRGHSALHIAIEKRSLQC
VKLLVENGANVHIRACGRFFQKHPGTCFYFGELPLSLAACTKQWDVVTYLLENPHQP
ASLEATDSLGNTVLHALVMIADNSPENSALVIHMYDGILQVGARLCPTVQLEDICNNQ
GLTPLKLAAKEGKIEIFRHILQREFSGLYQPLSRKFTEWCYGPVRVSLYDLSSVDNWEM
NSVLEIIAFHCRSPHRHRMVVLEPLNKLLQEKWQRLIPRFFFNFACYLAYMLIFTIVAY
HQPSLEKPAVPSSKATFGESMLLLGHILILLGGIYLLLGQLWYFWRRRLFIWISFMDSY
FEILFLVQALLTVLSQVLRFMETEWYLPLLVSSLVLGWLNLLYYTRGFQHTGIYSVMIQ
KVILRDLLRFLLVYLVFLFGFAVALVSLSREAQSPKAPADNNTTVTEQPMLGQEEEAA
PYGGILDASLELFKFTIGMGELAFQEQLRFRGVVLLLLLAYVLLTYVLLLNMLIALMSET
VNSVATDSWSIWKLQKAISVLEMENGYWWCRRRKHRSGRLLKVGTRWDGVPDERW
CFRVEEVNWAAWEKTLPTLSEDPSGEGILGYDKNPASKSGKNSVSEEDHLPLQVLQSQ

>TRPV2_Dipodomys_ordii

MTSPSSPPAFRLETSDGDQEDGTEAVKGKPGPPPMESPFQGEDRNFSPQIKVNLNYRK
GLKARQQDPNRFDRDRLFSIVSRGASEDLAGLLEYLRRTSKYLTDSEYTEGSTGKTCL
MKAMLNLQGVNACILPLLQIDRDSGNPQPLVNAQCTDEYYRGHSALHIAIEKRSLQCV
KLLVENGANVHARACGHFFQKHQGTCFYFGELPLSLAACTKQWDVVTYLLENPYQPA
SLQAADSLGNTALHALVMIADNSPENSALVIRMYDGLLQLGARLCPTVQLEDIHNLQG
LTPLKLAAKEGKIEIFRHILQREFSGLCQPLSRKFTEWCYGPVRVSLYDLASVDSWEEN
SVLEIIAFHCRSPHRHRMVVLEPLNKLLQAKWDLLIPRFFLNFLCYLVYMLIFTIVAYH
QPALDKQALSPMKATAGNSMLLFGHVLILLGGIYLLLGQTWYFWRRRLFIWISFMDSY
FEILFLVQALLTVLSQVLCFVNNEWYLPLLVSSLVLGWLNLLYYTRGFQHTGIYSVMIQ
KVILRDLLRFLLVYLVFLFGFAVALVSLSREAQITGVPTGDNATEAAQLSAGQEKEETP
YRGILDASLELFKFTIGMGELAFQEQLRFRGVVLLLLLAYVLLTYVLLLNMLIALMSETV

NSVATDSWSIWKLQKAISVLEMENGYWWCRRKKQRAGVMLTVGTRPDGSPDQRWC
FRVEEVNWASWEQTLATVCEEPSGAGISGSSKNPISSSQPVEDQDRDSEKDHLPLQVL
QSH--

>TRPV2_Tursiops_truncatus

MSSPSSPPAFRLETSDGGQEDGAQVDKGKLGSGAGPPPMESPFQGEDRNFSPQIKVNL
SFRKGAGPQPARPNRFDRDRLFSVVARGVPEDLVGLPEYLRRTSKYLTDSEYTEGSTG
KTCLMKAVLNLRDGTNACIEPLLQIDRDLGNPRPLVNAQCTDEYYRGHSALHIAIEKR
SLPCVKLLVENGADVHARACGQFFQKKSQETCFYFGEMPLSLAACTKQWDVVTYLLE
NPHQPASLQAADSLGNTALHALVMIADDSAENTEMVTRMYDGLLQAGARLCPTVRL
EDIPNLQGLTPLKLAAKEGKIEGRARSGLRTLSALPWCLHVYLLNAPLALAFPSQHRH
RMVVLEPLNKLLQVKWNLLIPRFLFNFLCYLTYMSIFTAVTYHQPALDKARQGGAHP
GEASWKQSWYFWRRRLFIWISFMDSYFEILLVQALLTVLSQVLCFLAIEWYLPLLVCSL
ALGWLNLLYYTRGLQHTGIYSVMIQKVILRDLLRFLLVYLVFLFGFAVALVSLSQEARS
PKAPQAQLRFRGVALLLLLTHVLLTYVLLLNMLVALMSETVSNVASDSWSIWKLQKAI
SVLEMENGYWWCRRKKQRAGVRLTVGTRPDGLPDERWCFRVEEEVNWAAWEQTL
PTVREEPSGPGGFTGALKNPALASQSSQDSAVEEDHVPLQLLESH------------------------
----

>TRPV2_Spermophilus_tridecemlineatus

MTSPSSPPAFRLETSDQNQEDGAEVDRGKHGPPPMESPFQGEDRNFSPQIKVNLNYR
KGLGPSQPDPNRFDRDRLFSVVTRGAPEDLAGLQEYLNRTSKYLTDSEYTEGSTGKTC
LMKAVLNLQDGVNACILPLLQIDRDSGNPQPLVNAQCTDEYYRGHSALHIAIEKRSLQ
CVKLLVENGADVHARACGHFFQKRQGTCFYFGELPLSLAACTKQWDVVTYLLENPHQ
RASLQAADSLGNTALHALVMIADNSKENSALVIHMYDDLLQAGARLCPTVQLEDICNL
EGLTPLKLAAKEGKIEIFRHILQREFSGLCQPLSRKFTEWCYGPVRVSLYDLASVDSWE
ENSVLEIIAFHCKSPHRHRMVVLEPLNKLLQAKWDMLIPRFFFNFLSYLIYMLVFTAV
AYHQPALEKQAFLPLKATGGNSMLLLGHILILLGGIYLLLGQLWYFWRRRLFIWISFVD
SYFEILFLLQALLTVLSQVLCFMAIEWYLPLLVSSLVLGWLNLLYYTRGFQHTGIYSVMI
QKVILRDLLRFLLVYLVFLFGFAVALVSLSREARDSGTPAGNNATEVAQPGTEQENEA
TPYRGILDASLELFKFTIGMGELAFQEQLRFRGVVLLLLLAYVLLTYVLLLNMLIALMSE
TVNNVATDSWSIWKLQKAICVLEMENGYWWCRRKKQRAGVLLKVGTRPDGTPDER
WCFRVEEVNWASWEQTLSTVREEPSEGNAPGSMKNPILASQPGEDHASKEDHLPLQ
VLQSH---

>TRPV2_Equus_caballus

MTSPPSPLDFRLETSDEVQEEDAKVDKGKLGHGDGPPPMESPFQGEDRNFSPQIKVN
LKYRKGAGASQPDPNRFDRDRLFSVVSRGVPEDLAGLPEYLRRTSKYLTDSEYTEGST
GKTCLMKAVLNLRDGANACILPLLQIDKDSGNPRPLVNAQCSDEYYRGHSALHIAIEK
RSLQCVKLLVENGADVHARACGHFFQKKSQGTCFYFGELPLSLAACTKQWDVVTYLL

ENPHQPANLQAADSLGNTALHALVMIADNSEENSALVIWMYDGLLRAGARLCPAVQL
EDIPNLQGLTPLKLAAKEGKIEIFRHILQREFSGPCQPLSRKFTEWCYGPVRVSLYDLAS
VDSWEENSVLEIIAFHCRSPHRHRMVVLEPLNKLQAKWDLLIPRFFFNFVCYLIYMFIF
TAVAYHQPALEKQAFSPMKVTAGNSMLLLGHILILLGGVYLLVGQLWYFWRRRLFIWI
SFMDSYFEILLVQALLTVLSQVLCFLAVEWYLPLLVSSLVLGWLNLLYYTRGLQHTGIY
SVMIQKVILRDLLRFLLVYLVFLFGFAVALVSLSREAWVPQVPTGPNVTEAVQSGAGQ
HDEEGSTAPYRGILDASLELFKFTIGMGELAFQDQLRFRGVVLLLLLAYVLLTYILLLN
MLIALMSETVNSVATDSWSIWKLQKAISVLEMEKGYWWCRRKKQRAGVLLTVGTRP
DGSPDERWCFRVEEVNWAAWEQTLPTVCEEPSGSSGAGTMKNPALTSQPGEESASE
GDQLPLQLLESH--

>TRPV2_Homo_sapiens

MTSPSSSPVFRLETLDGGQEDGSEADRGKLDFGSGLPPMESQFQGEDRKFAPQIRVNL
NYRKGTGASQPDPNRFDRDRLFNAVSRGVPEDLAGLPEYLSKTSKYLTDSEYTEGSTG
KTCLMKAVLNLKDGVNACILPLLQIDRDSGNPQPLVNAQCTDDYYRGHSALHIAIEKR
SLQCVKLLVENGANVHARACGRFFQKGQGTCFYFGELPLSLAACTKQWDVVSYLLEN
PHQPASLQATDSQGNTVLHALVMISDNSAENIALVTSMYDGLLQAGARLCPTVQLEDI
RNLQDLTPLKLAAKEGKIEIFRHILQREFSGLSHLSRKFTEWCYGPVRVSLYDLASVDS
CEENSVLEIIAFHCKSPHRHRMVVLEPLNKLLQAKWDLLIPKFFLNFLCNLIYMFIFTA
VAYHQPTLKKQAAPHLKAEVGNSMLLTGHILILLGGIYLLVGQLWYFWRRHVFIWISFI
DSYFEILFLFQALLTVVSQVLCFLAIEWYLPLLVSALVLGWLNLLYYTRGFQHTGIYSV
MIQKVILRDLLRFLLIYLVFLFGFAVALVSLSQEAWRPEAPTGPNATESVQPMEGQEDE
GNGAQYRGILEASLELFKFTIGMGELAFQEQLHFRGMVLLLLLAYVLLTYILLLNMLIAL
MSETVNSVATDSWSIWKLQKAISVLEMENGYWWCRKKQRAGVMLTVGTKPDGSPD
ERWCFRVEEVNWASWEQTLPTLCEDPSGAGVPRTLENPVLASPPKEDEDGASEENYV
PVQLLQSN---

>TRPV2_Ictidomys_tridecemlineatus

MTSPSSPPAFRLETSDQNQEDGAEVDRGKHGPPPMESPFQGEDRNFSPQIKVNLNYR
KGLGPSQPDPNRFDRDRLFSVVTRGAPEDLAGLQEYLNRTSKYLTDSEYTEGSTGKTC
LMKAVLNLQDGVNACILPLLQIDRDSGNPQPLVNAQCTDEYYRGHSALHIAIEKRSLQ
CVKLLVENGADVHARACGHFFQKRQGTCFYFGELPLSLAACTKQWDVVTYLLENPHQ
RASLQAADSLGNTALHALVMIADNSKENSALVIHMYDDLLQAGARLCPTVQLEDICNL
EGLTPLKLAAKEGKIEIFRHILQREFSGLCQPLSRKFTEWCYGPVRVSLYDLASVDSWE
ENSVLEIIAFHCKSPHRHRMVVLEPLNKLLQAKWDMLIPRFFFNFLSYLIYMLVFTAV
AYHQPALEKQAFLPLKATGGNSMLLLGHILILLGGIYLLLGQLWYFWRRRLFIWISFVD
SYFEILFLLQALLTVLSQVLCFMAIEWYLPLLVSSLVLGWLNLLYYTRGFQHTGIYSVMI
QKVILRDLLRFLLVYLVFLFGFAVALVSLSREARDSGTPAGNNATEVAQPGTEQENEA
TPYRGILDASLELFKFTIGMGELAFQEQLRFRGVVLLLLLAYVLLTYVLLLNMLIALMSE
TVNNVATDSWSIWKLQKAICVLEMENGYWWCRRKKQRAGVLLKVGTRPDGTPDER
WCFRVEEVNWASWEQTLSTVREEPSEGNAPGSMKNPILASQPGEDHASKEDHLPLQ
VLQSH---

>TRPV2_Echinops_telfairi_fragment

MTAPARGPAFRLETSDGGQEDGTEGDKGNLGHGDGPPPMESPFQGEDRNFSPQIKVN
LNYRKGQSASKPDPERFDRDRLFSVVSRGVPEGLAGLAEYLRKTSKYLTDSEYTEGSTG
KTCLMKALLNLQDGTNACILPLLRIDKDSGNPWALVNAQCTDDYYRGHSALHIAIEKR
SLPCVKLLVENGADVHARACGHFFQKKSQGACFYFGELPLSLAACIKQWDVVTYLLEN
PHQPASLQATDSLGNTVLHALVMIADNSAENSALVIRMYDALLRTSTRLCPTVRLEDI
PNRQGLTPLSWSAKEGKIELNKLLQPKWELLTPKFYINLLSYLVYMIIFTAVAYHQPAL
EKVRPGWHPLKPTAGNSMLLLGHILILLGGLYLLFGQLWYFWRRRFIWISFMDSYFEV
LLLLQALLTVLSQVLCLLAIEWYLPLLVSSLVLGWLNLLYYTRGFQHTGIYSIMIQKKAI
SVLEMENGYWWYRQKKQRAGVMLKVGTQLDGSPDERWCFWVEEVNWDSWEKTLP
TVCEEPAGAQVLGTTKNPTRSSQHEEDHASEEDHLPLQQLQ----------------------------
----

>TRPV2_Loxodonta_africana

MTSPSSPPVFRLETSDGGQEDGTEGDRRNLGHGDGPPPMESAFQGEDRNFAPQIKVN
LIYRKGSGACQPDRDRFDRDRLFNVVSRGAMEELAGLPEYLSWTSKYLTDSEYTEGST
GKTCLMKAMLNLQEGTNACILPLLQIDRDSGNPRPLVNAQCTDEYYRGHSALHIAIEK
RSLPCVKLLVENGADVHARACGHFFQKQSQGACFYFGELPLSLAACTKQWDVVTYLL
ENPHQPASLQAADSLGNTVLHALVMIADNSAENSALVIRMYDALLRAGARLCPSVNLE
DIPNLQGLTPLKLAAKEGKIEIFRHILQREFEGQCQPLSRKFTEWSYGPVRVSLYDLASV
DSWEENSVLEIIAFHCRSPHRHRMVVLEPLNKLLQRKWELLAPKFYFNFLCYLVYMII
FTTVAYHQPALEKQAFPPLKATAGNSVLLLGHVLILLGGLYLLLGQLWYFWRRRLFIW
LSFMDSYFEILYLFQALLTVLSQVLCLLAIEWYLPLLVSSLVLGWLNLLYYTRGFQHTG
MYSVMIQKVILRDLLRFLLVYFVFLFGFAVALVSLSRETQGPKAHTGLNATEAAHDGA
APYGGILDASLELFKFTIGMGELAFQEQLRFRGVVLLLLLAYVLLTYILLLNMLIALMSE
TVNSVATDSWSIWKLQKAISVLEMEGGYWWCRPKKQRVGVMLTVGTRPDGSPDER
WCFRVEEVNWATWEQTLPTVLEEPAGAPVPGTTKTPTLASQHEEGRASEEDHLLLQ
PLGPH---

>TRPV2_Sus_scrofa

MASPSSPPAFRLETSDRSREDGAERDRGQPGGGARPPPMESAFQPEDRMFPPQITVDL
HYRKRAGASQPDPNRFDRDRLFRVVARGVPEDLAGLPEYLRRTSKYLTDTEYREGST
GKTCLMKAVLNLRDGANACIQPLLQIDRDSGNPQPLVNAQCTDAYYRGHSALHIAIEK
RSLPCVKLLVENGADVHARACGQFFQKKSQDPCFYFGELPLSLAVCTKQWDVVTYLLE
NPHQSASPQAADSLGNTALHALVAIADNSVKNTELVTLMYDKLLQAVARLYPTVRLE
DIPNLQGLTPLQLAAKEGKIEIFRHILQREFPGPCQSLSRKFTEWSYGPVRVSLYDLASV
DSWEENSVLEIIAFHCRSSDRHRMVVLEPLNKLLQEKWNLLTPRFLINFLCYLIYVSVL
TATYHQPALEKCLSKQDLLTLGNSTLLLGHILILLTGAYLLMGQLWYFWRRRLFIWTS

FVDSYFEILFLVQALLAVLSQVLCFLAVKWYLPLLVSSLVLGWLNLLYYTRGLQHTGIY
SVMIQKVILRDLLRFLLVYLVFLFGFAVALVSLSREAQALGAPLGSNATEAAGEGDKEG
SEALYKGILDASLELFKFTIGMGELAFQEQLRFRGVVLVLLLAYVLLTYVLLLNMLIAL
MSETVNSVAHDSWSIWKLQKAICVLEMEKGYWWCRRKKQRAGVKLTVGTRPDGSP
DERWCFRVEEVNWAAWEQTLPTVCEEPSGPPGSSGDTNEPLASQPSQDSVTEEDNV
PLQPLESR--

>TRPV2_Monodelphis_domestica

MPEPARVPAFRLETTDSEEGGSTAAAASATGERGSRDQLPPMESAFQKENKDFAPQIK
VNLNYRKGVSPSSPTDPNRFDRDLFNAVLGGAPDSLEGLLEYLMLTSKYLTDSEFTEA
STGKTCLMKAVLNLKDGMNGCILPLLQIDHATRNPQPLINAVCIGEYYKGHSALHIAIE
KRSLQCVKVLVENGADLHAKASGSFFGKENRGVCFYFGELPLSLAACTRQWDIVSYLL
ENPDPDRRACIEAVDTWGNTVLHALVMIADDSVENSAQVNSMYDYILQIGARLWPSL
KLENLTNLQGLTPLKLAAKEGKIQVFKHILQREISGEYRHLSRKFTEWTYGPVQVSLYD
LSSVDSCEDNSVLEIIAFGCKAPNRHKMVTLEPLNKLLQEKWESLAARFRFNLISYFLY
MLIFTAVTYHQPVLGKNFIPLKATVGNSMLLMGHIFILFGGVYLFFGQLQYFWRRRLFI
WTSFIDSYFEILILIQALFSVLAQVLCFLELSWYLPFLVFSLVLGWINVLYYTRGFQQTGI
YSVMIQKVILRDLLRFLLVYFVFLFGFAIALVSLTREGPLPAVFNQTAAAAVAEEEEAK
VPYSGFVEASLELFKFTIGMGELEFHKELQFKGFVMFLLLAYVLLTYILLLNMLIALMSE
TVNSVATDSWSIWKLQRAISVLEMERGYWWCKRKKERSGILLTVGTTLDQKPDERW
CFRVEEVNWATWEKELQTLKEEPEGTSEPGKGPTGESAPRPTTSLE--------------------
----

>TRPV2_Normascus_leucogenys

MTSPSSSSVFRLETLDGGQEDGSEVDSRKPDFGSGLPPMESLFQGEDRKFAPQIKVNL
NYQKGTGVSQPDPNRFDRDRLFNAVSRGVPEDLAGLPEYLSKTSKYLTDSEYTEGSTA
KTCLMKAVLNLKDGVNACILPLLQIDRDSGNPQPLVNAQCTDDYYRGHSALHIAIEKR
SLQCVKLLVENGANVHARACGRFFQKGQGTCFYFGELPLSLAACTKQWDVVSYLLEN
PHQPASLQATDSQGNTVLHALVMISDNSAENVALVTSMYDGLLQAGARLCPTVQLEDI
RNLQDLTPLKLAAKEGKIEIFRHILQREFSGLSHLSRKFTEWCYGPVRVSLYDLASVDS
CEENSVLEIIAFHCKSPHRHRMVVLEPLNKLLQAKWDLLIPRFFLNFLCNLIYMFIFTA
VAYNQPTPKKQAAPHLKAEVGNSMLLAGHIFILLGGIYLLMSQLWYFWRRHLFIWISF
IDSYFEILFLFQALLTVVSQVLFFLAIEWYLPLLVSALVLGWLNLLYYTRGFQHTGIYSV
MIQKVILRDLLRFLLIYLVFLFGFAVALVSLSQEAWRPEAPTGPNATELVQPAEGQEDE
GNGVQYRGILEACLELFKFTIGMGELAFQEQLHFRGMVLLLLLAYVLLTYILLLNMLIA
LMSETVNSVATDSWSIWKLQKAISVLEMENGYWWCRKKQRAGVMLTVGTKPDGSP
DERWCFRVEEVNWASWEQTLPTLCEDPSGAVVPRTLENPVLASPPKEDEDGASEEN
DVPSKLLQSN--

>TRPV2_Ornithorhynchus_anatinus

MPEPARPTVFMLETTDSSPEERANASARPEGKGRKNELPPMESAFQKENSDFTPSIRV
NVNYHPSSPPPGGLPILNQQDPNRFDRDRLFGAVARGDPEELEGLKEYLQRTGKFLTN
SEYKVGSEGRAWNMNMVSSLVEGVFLMPTEYVLEESHVTVIIITLGSASPFVSLGHSAL
HIAIEKRSLTWVKVLVENGANVHAKADGQFFQKSKGICFYFGELPLSLAACTKQWDVV
EFLLENPHQPARLEEGDSQGNTVLHALVMIADNSKENTSLVSAMYDDILRAGARICPS
MKLEDIPNNQGLTPLKLAAKEGKIEIFKHILQREMSGEYQHLSRRFTEWSYGPVQAAL
YDLSSVDSSEDNSVLDIIVFGCRAPNRHKMVVLEPLNKLLQEKWESLAIRFYFNFACYV
IYMMIFTAVAYHQPVLGKTFLPLKATAGNSMLLMGHILILFGGFYLFFGQLRYFWKRH
RFLWITFVDSYFEILILVQSLTTVVAQVLCFMELEWYLPVLVFSLVVGWVNTLYYTRGF
EPTGIYSVMIQKVILRDLLRFLLIYFVFLFGFAVALVSLTREALPLPPLNTSLTHPEVGGG
NDDDGKTSYSSMFAASLELFKFTIGMGELEFQEHLRFKGFVMFLLLVYVLLTYILLLNM
LIALMSETVNSVSTDSKSIWKLQRAIAVLEMEMGYWWCRRKKKRSGTFLTVGVTPDQ
KTDERWCFRVEEVNWAMWERELQALKEDPGDCRDLVKNPTGKAWKRLLSTQRPE
DSTPEEDQPLRP--

>TRPV2_Oryctolagus_cuniculus

MTSPSSPPAFRLETSDGGQDGAEVDKAQLGYGAGPPPMESRFQDEDRNFPPQIKVNL
NYRKGAGASQPDLNRFDRDRLFNVVARGNPEDLAGLLEYLRRTSKYLTDSEYTEGSTG
KTCLMKAVLNLQDGVNACIQPLLEIDRDSGNPQPLVNAQCTDEYYRGHSALHIAIEKR
SLQCVKLLVENGANVHAKACGHFFQKNQDTCFYFGELPLSLAACTKQWDVVNYLLEN
PHQPASLQAQDSLGNTVLHALVMIADDSAENSALVVRMYDGLLQAGARLCPNVQLEG
IPNLEGLTPLKLAAKEGKIEIFKHILQREFSAPCQSLSRKFTEWCYGPVRVSLYDLASVD
SWEENSVLEIIAFHSRSPHRHRMVVLEPLNKLLQAKWDRLIPRFCFNFLCYLVYMLIFT
AVAYHQPALEKQGFPPLKATAGNSMLLLGHILILLGGVYLLLGQLWYFWRRRLFIWIS
FMDSYFEILFLLQALLTVLSQVLCFLAIEWYLPLLVSSLVLGWLNLLYYTRGFQHTGIYS
VMIQKVILRDLLRFLLVYLVFLFGFAVALVSLSREAQNSRTPAGPNATEVGQPGAGQED
EAPPYRSILDASLELFKFTIGMGELAFQEQLRFRGVVLLLLLAYVLLTYVLLLNMLIALM
SETVNSVATDSWSIWKLQKAISVLEMENGYWWCRRKKQRAGVMLTVGTRPDGSPDE
RWCFRVGEMNWATWEQTLPRTLCEEPSGAAAPGVMKNPTPASQRGEDSASEEDHL
PLQLLQSR---

>TRPV2_Sarcophilus_harrisii

MPEPAQGPAFRLETTEGDQGGGSAAAAAGERGSRDQLPPMESEFQKESTDFSPQIKV
NLNYRKGMGPSPTDPNRFDRDRLFNAVVGGNPESLDGLLGYLLLTSKYLTDSEFTDAS
TGKTCLMKAVLNLREGMNACILPLLQIDLATRNPQPLINAVCTGDYYKGHSALHIAIEK
RSLECVKVLVENGADLHARARGSFFGKERQGICFYFGEWTGLSLDTTGQSWGWMVPY
FLNKGNPSNGHPKEAIDVLCFSVNFIYELFQSTLFPRESLVIKKREKSVQLLFQPCDWR
PLRSMAVVPWRVTDPHSWPEVFKHILQREISGEYRHLSRKFTEWTYGPVQVSLYDLS
SVDSCEDNSVLEIIAFGCKAPNRHKMVTLEPLNKLLQEKWESLVTRFRFNLISYFLYMV
IFTIVTYHQPVLGKNFIPMKATVGNSMLLMGHIFILFGGVYLFFGQLQYFWRRRLFIWT
SFIDSYFEILILSQALTSVVAQVLCFLELSVYLPVLVFSLVLGWINLLYYARGFQQTGIYSV
MIQKVILRDLLRFLLVYFVFLFGFAIALVSLSREAPLPTFSNQSLAGADKPPYGGLVEAS
LELFKFTIGMGELEFHKELQFEGFIMFLLLAYVLLTYILLLNMLIALMSETVNSVATDS

WSIWKLQRAISVLEMERGYWWCKRKKERSGILLTVGTTPDKKPDERWCFRVEEVNW
ATWEKELQTLKEEPESTLEMGKTLPVLLRDGKGIRAQEQHPPPHLKTLLTH-----------
----

>TRPV2_Pan_troglodites

MTSPSSSPVFRLETLDGGQEDGSEADRGKLDFGSGLPPMESQFQGEDRKFAPQIRVNL
NYRKGTGASQPDPNRFDRDRLFNAVSRGVPEDLAGLPEYLSKTSKYLTDSEYTEGSTG
KTCLMKAVLNLKDGVNACILPLLQIDRDSGNPQPLVNAQCTDDYYRGHSALHIAIEKR
SLQCVKLLVENGANVHARACGRFFQKGQGTCFYFGELPLSLAACTKQWDVVSYLLEN
PHQPASLQATDSQGNTVLHALVMISDNSAENIALVTSMYDGLLQAGARLCPTVQLEDI
CNLQDLTPLKLAAKEGKIEIFRHILQREFSGLSHLSRKFTEWCYGPVRVSLYDLASVDS
CEENSVLEIIAFHCKSPHRHRMVVLEPLNKLLQAKWDLLIPKFFLNFLCNLIYMFIFTA
VAYHQPTLKKQAAPHLKAEVGNSMLLTGHILILLGGIYLLVGQLWYFWRRHVFIWISFI
DSYFEILFLFQALLTVVSQVLCFLAIEWYLPLLVSALVLGWLNLLYYTRGFQHTGIYSV
MIQKVILRDLLRFLLIYLVFLFGFAVALVSLSQEAWRPEAPTGPNATESVQPLEGQEDE
GNGAQYRGILDASLELSKITIGRGELDFQEQLHFRGMVLLLLLAYVLLTYILLLNMLIAL
MSETVNSVATDSWSIWKLQKAISVLEMENGYWWCRKKQRAGVMLTVGTKPDGSPD
ERWCFRVEEVNWASWEQTLPTLCEDPSGAGVPRTLENPVLASPPKEDEDGASEENYV
PVQLLQSS--

>TRPV2_Pongo_abelii

MTSPSSSPVFRLETLDGGQEDGSEADRGKLDFGSGLPPMESQFQGEDRKFAPQITVNL
NYRKGAGASQPDPNRFDRDRLFNAVSRGVPEDLAGLPEYLSKTSKYLTDSEYTEGSTG
KTCLMKAVLNLKDGVNACILPLLQIDRDSGNPQPLVNAQCTDDYYRGHSALHIAIEKR
NLQCVKLLVENGANVHARACGRFFQKGQGTCFYFGELPLSLAACTKQWDVVSYLLEN
PHQPASLQATDSQGNTVLHALVMISDNSAENIALVTSMYDGLLQAGARLCPTVQLEDI
RNLQDLTPLKLAAKEGKIEIFRHILQREFSGLSHLSRKFTEWCYGPVRVSLYDLASVDS
CEENSVLEIIAFHCKSPHRHRMVILEPLNKLLQAKWDLLIPKFFLNFLCNLIYMFIFTAV
AYHQPTLKKQAAPHLKAEVGNSMLLTGHILILLGGIYLLVGQLWYFWRRHVFIWISFI
DSYFEILFLFQALLTVVSQVLCFLAIEWYLPLLVSALVLGWLNLLYYTRGFQHTGIYSV
MIQKVILRDLLRFLLIYLVFLFGFAVALVSLSQEAWRPEAPTGPNATESVQPVEGQEDE
GNGAQYRGILEASLELFKFTIGMGELAFQEQLHFRGMVLLLLLAYVLLTYILLLNMLIAL
MSETVNSVATDSWSIWKLQKAISVLEMENGYWWCRKKQRAGVMLTVGTKPDGSPD
ERWCFRVEEVNWASWEQTLPTLCEDPSGAGVPRTLENPVRASPPKEDEDGASEENY
VPLQLLQSN--

>TRPV2_Otolemur_garnettii

MTSPSSPPVFRLETSDGDREDGAEVDKGKPGFGSGPPPMESPFQGEDRNFPHQIKVNL
NYRKKAGASQPDPNRFDRDRLFSVVSRGVAEDLAGLREYLRRTSKYLTDSEYTEGSTG
KTCLMKAVLNLQDGVNACILPLLQIDRDSGNPRPLVNAQCTDEYYQGHSALHIAIEKR
SLQCVKLLVENGADVHARACGQFFQKGQGTCFYFGELPLSLAACTKQWDVVSYLLDN

EHQRASLQATDSLGNTVLHALVMIADNSKENSDLVTHMYDKLLKAAADTDHTVQLE
DIRNLQGLTPLKLAAKEGKIEIFRHILQREFPGPYQALSRKFTEWCYGPVRVSLYDLAS
VDSWEENSVLEIIAFHCRSPHRHRMVVLEPLNKLLQEKWDLLIPRFFFNFLCYLTYML
VFTAVAYHQPALNKALLPLKATAGNSMLLLGHILILLGGICLLLGQLWYFWRRRLFIWI
SFMDSYFEILFLVQALLTVLSQVLCFLAISWYLPLLVSSLVLGWLNLLYYTRGFQHTGM
YSVMIQKVILRDLLRFLLVYLVFLFGFAVALVSLSREALDPRPTVGPRPTVGPNTTEAA
GQPGAGQEDEEGVEVPYRGILDASLELFKFTIGMGELAFQEQLRFRGVVLLLLLAYVLL
TYVLLLNMLIALMSETVNRVTNDSWSIWKLQKAISVLEMENGYWWCRRRKQRAGVR
LTVGTRPDGSPDERWCFRVEEVNWAAWEQTLRTVCEEPSGTGAPGTKKDLTLASRP
QEAGALEEDHLPLQLLQSH--

>TRPV2_Mus_musculus

MTSASNPPAFRLETSDGDEEGSAEVNKGKNEPPPMESPFQGEDRNFSPQIKVNLNYR
KGLGPSQQDPNRFDRDRLFSVVSRGVPEELTGLLEYLRRTSKYLTDSAYTEGSTGKTCL
MKAVLNLQDGVNACILPLLQIDRDSGNPQPLVNAQCTDEFYRGHSALHIAIEKRSLWC
VKLLVENGANVHIRACGRFFQKHQGTCFYFGELPLSLAACTKQWDVVTYLLENPHQP
ASLEATDSLGNTVLHALVMIADNSPENSALVIHMYDSLLQMGARLCPTVQLEDICNHQ
GLTPLKLAAKEGKIEIFRHILQREFSGLYQPLSRKFTEWCYGPVRVSLYDLSSVDSWEK
NSVLEIIAFHCKSPHRHRMVVLEPLNKLLQEKWDRLIPRFFFNFACYLVYMIIFTIVAY
HQPSLEQPAIPSSKATFGDSMLLLGHILILLGGIYLLLGQLWYFWRRRLFIWISFMDSYF
EILFLVQALLTVLSQVLRFVETEWYLPLLVSSLVLGWLNLLYYTRGFQHTGIYSVMIQK
VILRDLLRFLLVYLVFLFGFAVALVSLSREARSPKAPEDSNTTVTEKPTLGQEEEPVPYG
GILDASLELFKFTIGMGELAFQEQLRFRGVVLLLLLAYVLLTYVLLLNMLIALMSETVNS
VATDSWSIWKLQKAISVLEMENGYWWCRRKRHRAGRLLKVGTKGDGIPDERWCFRV
EEVNWAAWEKTLPTLSEDPSGAGITGYKKNPTSKPGKNSASEEDHLPLQVLQSH------

>TRPV2_Oryzias_latipes_fragment

GQETAEPPMDTDYHEEKEKPAPQLRFNLGFDKLARGKEQNKRDTRFTRDLLFEAAAS
GDVQKLEGLEDYLRLNMKNLSDSLCKYQSYGKTPLIKALMHLKDGKNKTVEIFINTAK
NIGDLEKFVNAAHTSNYYKGQTALHVAIERRSLPYVQLLVNSHADVHAKVSGKFFQPH
DGPCFYFGELPLSLAACTNQPEMVDYLLKNEIQRADPEQRDSHGNTVLHALVAVADN
SKENTEFINSMYDRILKITAKLHPKKKLEDIKNNKGLSPLLMAAKTGKIGVFSHILKRE
FHESDIKHLSRKFTEWVYGPVHCSLYDLASVDSCENNSLLEILIYGSDIPNRHEMLQTE
PLSQMLQAKWKKFAGGMFLINFLVYSLYLTIFTFVAHYIRGTAEYREFPFPLKSYDDYL
FATGLLLTLLANLFLFIAGITDMWRKRPNMMTVLIDGYYEILFCVQGLFYLCFAVLYVA
GLKEYVCFLVLCLALSWVNVLYFSRGYQHMGIYSVMIQKIIVCDILRFLFVYVVFLFGFS
AAVVTLLIVPPEKPRNATKGRSFFTTQAPGEDCFIPTYKNFSFTVLELFKFTIGMGDME
FSQAFQYTEIFYFLLIGYIILTYILLLNMLIALMNRTVENITKESTCIWKLQRAVTILDME
KRLPYCLRKRLRCGVEKKLCTALGNDQRWCFRVEEVNWNKWNTDIGKIDEDP--------

>TRPV2_Salmo_salar

MSKSKGPEYPSFSLETDDRTDDERAQSRQVKKPDRLVSALGLGSSGGPKTPMDSDYQ
DELEEAAPKIRFNLNFDKEVRCLEENKEDRVSKRFDIKRLFEAVSTGDVMKLEGLHQY
LHQSMKKLSNTEYQSYGKNVLLKALLNLRKGRNNTIEYLLDISEKMGDIKEFVNAAYT
DSYYKGQTALHIAIERRSIYFVELLIKKGANVHAKACGKFFQAHDGPSFYFGELPLSLAA
CTNQPEVVDFLLENDYQRVDVRESDSLGNMVLHALVVLADNTPENTDFITSMYDHIL
TTTARLHPEWRLEDIENNQGLTTIKLAAKTGKIGLFKHMMHREFQERETRHLSRKFT
EWVYGPVHSSLYDLASLDSYEKNSVMEIIVYSSDIPNRHEMLQIEPLNRLLEEKWDKF
AARMFFLNFLVYLVYLSVFTAVAYNRKKGTPPFTLEHTRQEYLRLAGQLFITVGACYFF
IRGILDLKRKRPSLDTLLIDGYSEILFFLQAIFFLASLVLYCCGREEYLGFLVLCLALSWV
NLLYFSRGYRHMGIYSVMIQKMILSDILRFLFVYVTFLFGFSAAVVTLLMEPELPASNT
AQPINSTDGKGRTLFLPTEDSCIKPTFRNISHTIMELFKFTIGMGDLEFTEGYQYKEVFY
MLLISYIVLTYILLLNMLIALMSRTVEKMSLESTSIWKLQRAITILDLERSLPRCLRRRLR
SGVDKDLGTRAGEKDRRWCFRVEEVNWNKWNTNLGIINEDPGSGDTARLSPSHSSR
TLGKERSWRGFLGNVSRRQHTQPQHQIQVESTEMSSLSPLSHV-------------------------
----

>TRPV2_Gasterosteus_aculeatus

MEKSEIGFSLEADDRTEEERARQKASKKGGVASALGLGKEAPKVPMDTDYQEEMEKP
KPQIRFNLNFDKGIRGEQRNKKDSSTFTIDRLFEAAASGDVGKLENLHQYLRQNTKKL
SDSLYRSYGKTALMKALLHLRNGKNETVELLIDISEKMGDIKEFVNAAYTNNYYKGQT
ALHVAIERRNISYVKLLVSKGADVHAKAWGLFFQPHDGPNFYFGELPLSLAACTNQPA
VVDFLMENEYQRADAKMTDSHGNTVLHALVTVAASSKDQDKDNTEFITKMYDRILK
TSAKLHPNLKLEGIENNKGLTPLKMAAKTGKIGLFTHILKREFHESHTKHLSRKFTEW
VYGPVQSSLYDLASVDSYEDCSVVEILVYGSDIPNRHAMLQMEPLSQLLEEKWNKFAG
RMFFLNFLVYLVYLSIFTLVAYNKKVGQLPYPIEHTTKGYLYVSGQVVTAVANCYFFFI
GILDMKRKRPKLQTLLIDGYYEFLFFVQGVLFLITAVLYLCGKKQYLSFLVLCLALSWV
NMLYFSRGDRHMGIYSIMIQKMILSDILRFLLVYIVFLFGFSAAVVTLLIEPEVKNSTLSQ
PSKGRIFGPLDPGEECVKPSFTSISYTTLQLFKFTIGMGDMEFTQEHENTEVFYLLLIGY
IILTYILLLNMLIALMNRTVEKTTEESTSIWKLQRAITILEMEKRLSCCLRRRFRCGVEK
NLRTALGDDRRRCFRVEEVNWNKWNINLGKINEDP--------------------------------------
----

>TRPV2_Xenopus_tropicalis

MSGTDSDGPPNGTLNFEFHDKDSQAAFDKADKNSDGKKEKKPPMNSAIQKESDNYSP
LIGINVNFLPKVTGHGKQPVLDPSQVYDRNRIFTAVTNGDTQDLAGLQNYLDKTMKR
LTNTEFKDPDTGKTCLLKAMWNLKDGMNDTIPILLEAAEKGGYLKELVDAAYTNDYY
RGQTALHIAIEKRNMHLVQLLIKSGADLRAMAEGDFFQKKKKGISFYFGELPLSLAACT
NQLNIVNYLLDKKADLAARDSHGNTVLHALVFVSDNTEENTEIVTKMYDEILKKSVKT
DPTLKIEEITNWEGLTPLKLAAKTGKIELFKHILRREVTEPEYKHLSRKFTEWTYGPV
HTSLYDLSSVDTHEADSVLETIVFKSNASNRHNMVVLEPINTLLEEKWENFAGKIFYIK
FLLYILYMIIFTVTAYHRPLQGQPPFPLEGTAKGNLRVIGEIIMMFGAIYILICQIIYFWKR
RPSLQILMMDGYFEILFFLQALILLCSGATYLAGSEVYVALMVFSLVIGWVDMLYYTRG
FQQTGIYSVMIQKTILRDMTLNGEAPQEAANGTEQSESGSSYGGLYITSLELFKFTIGMG

DLEFNENLKFKHFFMFNMLIALMWKLQRAATILDIERFIPRFIRKKLKIGQWLTVGKS
PDGNPDKRWCFRVEEMVWGSWEKDLANINEEPGDTEKGKLSSSKKTPGKGNLEHKY
FLTIMFFLNSY--

>TRPV2_Danio_rerio

MSKSMDSSVSSFSLETDDQTEEERTKAKQMKKVSKDKRPMDSNYVDDVVEPSSTIKF
NLHFDRGIRNLKEEPAQQDNDRFTIKRLFEAVSSGDVSKMQGLHEYLHKNMKRLTDS
QYKSNGKTALLKALLNLRQGENDTIEQLLDIAEKMGDLKNFINAAYTDSYYKGQTALH
VAIERRSMKFVQMLVKKGADVHAKACGKFFQPNQKMCFYFGELPLSLAACTNQQDIV
DFLMENPHQAVDVRERDCHGNTVLHALVSVADNSPENTEFVIAMYDHILIKADQLHP
KTKLEEIENNEGLTPITLAAKKGKLGLFKHIVQRELMGCRHLSRKITEWAYGPVCSSLY
DLSSLDTYEKNSALEIVVYGSEIPNRLEMLQIEPLNRLIEEKWDQFAHRMFLFNFIVYVI
YLFIFTASAFYHEEGKDYANQTKPPYLYAKSREGYLLLTGHIISITGAFYFFIRGLIDMVR
KRPRFQSLIIDGYTDQLFFVQGLLFLASVVLYCYGQYEYLAFLVLCLALSWINLLYFSRG
SKNLGIYNVMIQKMVLGEIRRFLVVYMVFLIGFSAALVTLLDQESIDSGSTRDFRLSEDI
PSLNPTPDSSNPQSRMTHHQPTTARDGRGRFGLTTDNQYEVCKKPSYKNIYFTTLELF
KFTIGMGDLEFTDHYKYKEVFYVLLIVYIVMTYILMLNMLIALMNQSVEMMSVESTSI
WKLQRAITTLDMEWILPKCLQGKLRSGEEKDLGGGQEPDRRWCFSVEEVNWTQWN
RNMGIIINEDPGKCTQDPSPANVQREPSRGVLQTFSRRRRTQRAQTREGHELSPLAEA
SSSV---

>TRPV2_Gallus_gallus

MDNFTNDQSGPCQRNRLVGLLETDDSIQEDKPTSSTKEERSGQYGKKGEPRPLESPYQ
KESSDYSPKVMINLNYRPGFVSNQKDPNRFDRDRLFSAVSRGSPEALDGLLEYLRRTS
KFLTNSEYTDAKTGKTCLMKALLNLKNGRNDTIPVLLEIDQKTQNPRPLVNVACSDCY
YRGQTALHIAIEKRSLDLVKVLVEHGADVHARAHGEFFRKKKEGVCFYFGELPLSLAA
CTNQFEVVEYLLNNPHQKARLQEQDTQGNTVLHALVMIADDTEENTKFVSTVYYVEIL
KAGVKTDPTVKLEEIVNYDGLNPLQLAAKTGKVEIFRHIIQREIKDPVYRHLSRKFTEW
TYGPIHVSLYDLSSLDSFEENSVLEILAYSSDTPNRYKMVVLEPLNKLLQQKWETFASK
RFYFSFISYLSFMIIFTAIAYYQPLRVKPSFPVEFTAGGFLWVSGLIIILLGGVYLIFAQSLY
LRRRRQSLKTMCSDSCVEILIFIQAFSLLLSAVLYGASSENYVAVMVFSLLLGWVNMLY
YTRGFQRIGIYSVMIQKTILRDLLRFLLVYIIFLFGFAAALVTLMGDAPSLSQNKSVAQM
ENAGSHAMYGGLLSVCLELFKITIGMGDLDFHEHARFRYFVMLLLLLFVILTYILLLNM
LIALMSETVTDISGYSKSVWKLQRAIAILEIEKAWLWRQGGKRRSGCLMSVGLNKKDE
RWCFRVEEIKWTSWAKEVGVLKEEPGNTNDLEINPEETRSRKQVPQKQLRSAVSEEQ
SLLQPELSATEMMPLKGQTRNL--

>TRPV2_Meleagris_gallopavo

MDNFTNDQSGPCQRNRLVGLLETDDSIQEDKPASSTKEERSGQYGKKGEPRPLESPYQ
KESSDYSPKVMINLNYRPGLVSNQKDPNRFDRDRLFSAVSRGSPEALDGLLEYLRRTS
KFLTNSEYTDAKTGKTCLMKALLNLKNGRNDTIPVLLEIDQKTQNPRPLVNVACSDCY

YRGQTALHIAIEKRSLDIVKVLVENGADVHARAHGEFFRKKNEGVCFYFGELPLSLAAC
TNQFEVVEYLLNNPHQKARLQEQDTQGNTVLHALVMIADDTEENTKFVSTVYVEILK
AGVKTDPTVKLEEIVNYDGLNPLQLAAKTGKVEIFKHIIQREIKDPVYRHLSRKFTEWT
YGPIHVSLYDLSSLDSFEENSVLEILAYSSDTPNRYKMVVLEPLNKLLQQKWETFASKR
FYFSFISYLSFMIIFTAIAYYQPLRVKPSFPVEFTAGGFLWVSGLIIILLGGVYLIFAQSLYL
RRRRQSLKTMCSDSCVEILIFIQAFSLLLSAVLYGASSENYVAVMVFSLLLGWVNMLYY
TRGFQRIGIYSVMIQKTILRDLLRFLLVYIIFLFGFAAALVTLMGDAPSLSQNKSVAQME
SAGSHAMYGGLLSVSLELFKITIGMGDLDFHEHARFRYFVMLLLLLFVILTYILLLNMLI
ALMSETVTDISGYSRSVWKLQRAIAILEIEKAWLWRQGGKRRSGCLMSVGLNKKDER
WCFRVEEIKWTSWAKEVGVLKEEPGNTSDLEINPEASC---------------------------------
----

>TRPV2_Taeniopygia_guttata_fragment

LETDDSVQEDKPAPSKKEERSGHGRKPLETPYQKESSDFAPKIKVNLNYRPGSQKDPN
RFDRDRLFSAVVRGSPEALDGLLEYLRRNSKFLTNSEYTGKTCLMKALLNLKNGKND
TIPVLLEIDQKTQNPRPLVNVSCSDCFYRGEEGLIFRHIIQREIKDYRHLSRKFTEWTYG
PIHVSLYDLSSLDSFEENSVLEILAYSSDTPRYKMVVLEPLNKLLQQKWETFASKFYFSF
VSYLSFMIIFTAIAYYQPQAFSLLLSAVLYGASSENYVAVMVFSLLLGWVNTLYYTRGFQ
RTGIYSVMIQKIMRDLLRFLLVYMIFLFGFAESSGSHAMYGGLLSVSLELFKITIGMGDL
DFQEHTRFRYFVTLLLLLFVILTYILLLNMLIALMSETVTDISGYSKSVWKLQRAITILDI
ENSYLNCRHSFRSGKQVLVGITPDGQDDYRWCFRVDEVNWSTWNTNLGIINEDPGYS
G--

>TRPV2_Melopsittacus_undulatus_fragment

MPDPSSAVSYFPYFNGDYLAVAASLNGGNVLATFVDMVAQWTEELGLQIQESAIYTKII
EAALAQTNSKLSIHPTIFGERHVPEQLASVSNIAVSDLSLGHVTRALCRGIVENLCSMLP
VQCLMETGVRRILGSGSALARNEVLRQEVERVFPFPVVYGKDVDAAMGAAMFISLNK
VPAEMEYGCLLIMSSILGKMKKFGSSDMEESEATDEHTDGEDSTLETSDSLQSTLNTK
VHPPKSNIFARRGRFVMGDSDKDMAPMDSLYQMDHLLAPSVIKFHANLERGKFHKRI
FDAVAQGNTRDLDDLLLYLNRTLKHLTDDEFKETGKTCLLKAMLNLHDGKNDTIPLL
LDIAKKTGTLKEFVNAEYTDNYYKGQTALHIAIERRNMYLVKLLVQNGADVHARACG
EFFRKIKGKPGFYFGRGELPLSLAACTNQLCIVKFLLENPYQAANIAAEDSMGNMVLH
TLMYNNILILGAKINPILKLEELTNKKGLTPLTLAAKTGKIGVAVVTLIEDDNEGQDTNI
SEYSRCCHVKRGRTSYNSLYYTCLELFKFTIGMGDLEFTENYRFKSVFVILLVLYVILTYI
LLLNMLIALMGETVSKIAQESKSIWKLQVHWFCEAVIQERGVDEVNWSTWNTNLGII
NEDPGCSGDLKRNPSYSIKPGRVHLYLIYIKYMSRDICLFLWMGNVFAHKLLCILKVLG
QTALNIAIERRQYEITQSLIEKGADVNAHAQGIFFNPKHKHEGFYFGWGSANPEVAEL
QKAANNGSEEKSPRAKVWNRNEYTPLSNMKDSRPESGIIRKFENLSNSVKTVQQQNP
PNNRILLHNSIKKEKILVSLNPDLFLTSKEVQLSTDSDGKAEQNISKLALTWELCYVPT
QGPSQGADSPLTGSTRQQDSDDGQQRAEGQQGTATCTQHGTCLKEYGGCQQAFKQH
EGTSVPVLWQPHQDLSFHGKFHETLLALVAGTYCRYRWNS

>TRPV2_Anolis_carolinensis_fragment

RLLSSASIGVRAEKAFKIYSRRRLFDAVAQGDPSDLDDLFLYLMETMKNLTDDEFKEP
DTGKTCLLKAMLNLHNGWNDTIPLLLDIAKKTDNLKEFVNAEYTDGYYKGQTALHIA
IERRNYYLVELLLKNGADVHARAHGEFFQNIKGRPGFYFGELPLSLAACTNQLNIAKYL
LENPFQPADIAAQDSMGNTVLHALVEIADNTEDNTKFVTNVYNEILVLGARINPTLKL
EEIPNRRGLTPLTLAAKAGKIQVFAYILRREMKAPECRHLSRKFTEWAYGPVHSSLYD
LSSIDTCEKNSVLEIIAYSSETPNRHEMLLVEPLNQLLQDKWDRFVKHLFYFNFFTYTA
HILILTVAAYFRPTKKDGVPPFPLRHTTSEYFRVAGEILSILGGVYFFFRGLKYFKHRRP
SLKAVFTDSYSELLLLYVHTIYYIISIAQY--

>TRPV2_Chrysemys_picta_bellii_fragment

MYDEILKAGTKINPAEKLEEIVNQAGLTPLKLAAKKGKVEIFRHILQREIKDPEYRHLS
RKFTEWTYGPVHVSLYDLSSVDSYEKNSVLEILAESSDTPTILRDLLRFLMVYVIFLFGF
AAALVTLTGDAPPASQNTSVTPEDGDKGHVVYSGLLSTSLELFKFTIGMGDLEFQEHV
YDQNVLSPCLIVYWLMGKIVLFSDFRVEEINWANWEKELGVIKEDPGNSRDLEIESLA
VTPLSSFNIDTRKKWITPPTAPLSWPKVLQHSDGRKTW---------------------------------
----

>TRPV3_Rattus_norvegicus

MNAHSKEMVPLMGKRTTAPGGNPAVLTEKRPADLTPTKKSAHFFLEIEGFEPNPTVT
KTSPPIFSKPMDSNIRQCLSGNCDDMDSPQSPQDDVTETPSNPNSPSANLAKEEQRQK
KKRLKKCIFAAVSEGCVRELRELLQDLQELCRRRRGLDASDFLMHKLTASDTGKTCLM
KALLNINPNTKEIVRILLAFAEENDILDRFINAEYTEEAYEGQTALNIAIERRQGDITAV
LIAAGADVNAHAKGVFFNPKYQHEGFYFGETPLALAACTNQPEIVQLLMENEQTDITS
QDSRGNNILHALVTVAEDFKTQNDFVKRMYDMILLRSGNWELETMRNNDGLTPLQL
AAKMGKAEILKYILGREIKEKPLRSLSRKFTDWAYGPVSSSLYDLTNVDTTTDNSVLEII
VYNTNIDNRHEMLTLEPLHTLLHMKWKKFAKYMFFLSFCFYFFYNITLTLVSYYRPRE
DEALPHPLALTHKMSWLQLLGRMFVLIWAMCISVKEGIAIFLLRPSDLQSILSDAWFH
FVFFVQAVLVILSVFLYLFAYKEYLACLVLAMALGWANMLYYTRGFQSMGMYSVMIQ
KVILHDVLKFLFVYILFLLGFGVALASLIEKCSKDKKDCSSYGSFSDAVLELFKLTIGLGD
LNIQQNSTYPILFLFLLITYVILTFVLLLNMLIALMGETVENVSKESERIWRLQRARTILE
FEKMLPEWLRSRFRMGELCKVADEDFRLCLRINEVKWTEWKTHVSFLNEDPGPIRR
TADSNKIQDSSRSNSKTTLYAFDELDEFPETSV---

>TRPV3_Homo_sapiens

MKAHPKEMVPLMGKRVAAPSGNPAILPEKRPAEITPTKKSAHFFLEIEGFEPNPTVAK
TSPPVFSKPMDSNIRQCISGNCDDMDSPQSPQDDVTETPSNPNSPSAQLAKEEQRRKK
RRLKKRIFAAVSEGCVEELVELLVELQELCRRRHDEDVPDFLMHKLTASDTGKTCLMK
ALLNINPNTKEIVRILLAFAEENDILGRFINAEYTEEAYEGQTALNIAIERRQGDIAALLI
AAGADVNAHAKGAFFNPKYQHEGFYFGETPLALAACTNQPEIVQLLMEHEQTDITSR
DSRGNNILHALVTVAEDFKTQNDFVKRMYDMILLRSGNWELETTRNNDGLTPLQLA
AKMGKAEILKYILSREIKEKRLRSLSRKFTDWAYGPVSSSLYDLTNVDTTTDNSVLEIT
VYNTNIDNRHEMLTLEPLHTLLHMKWKKFAKHMFFLSFCFYFFYNITLTLVSYYRPR
EEEAIPHPLALTHKMGWLQLLGRMFVLIWAMCISVKEGIAIFLLRPSDLQSILSDAWF
HFVFFIQAVLVILSVFLYLFAYKEYLACLVLAMALGWANMLYYTRGFQSMGMYSVMIQ
KVILHDVLKFLFVYIVFLLGFGVALASLIEKCPKDNKDCSSYGSFSDAVLELFKLTIGLGD
LNIQQNSKYPILFLFLLITYVILTFVLLLNMLIALMGETVENVSKESERIWRLQRARTILE
FEKMLPEWLRSRFRMGELCKVAEDDFRLCLRINEVKWTEWKTHVSFLNEDPGPVRR
TDFNKIQDSSRNNSKTTLNAFEEVEEFPETSV---

>TRPV3_Sarcophilus_harrisii

MSKNPKEMVPLMGKKTGTPSAAPVCPLERRPAEVTPTKKSAHFFLEIEGFEPNPAAN
KPSPPIFPKPMDSNIRQCNPGHCDDMDSPQSPQDDITETPSNPNSPCVSLAKEEQKRN
RKRLKKRLFAAAAEGSVEELSVLLTELWELARRRRTMDPMDYLMHKLTASDTGKTC
LMKALLNINPNTEEVVRTLLTFAEENGILEQFINAEYTEEAYRGQTALNIAIERRQVAI
TKTLIDNGADVNAHAKGLFFNPKHKHEGFYFGETPLALAACTNQPEIVQLLMDNEKT
DIASQDSQGNNILHALVTVAEDCRTQNDFVKEMYDMILLRSEDPELETAQNNDGLTP
LQLAAKTGKSEILKYILSREIKEKPHRSLSRKFTDWAYGPVSSSLYDLTDVDTTTDNSV
LEIIVYNTNIDNRHEMLTLEPLHTLLRMKWKKFAKYMFFTSFCFYFFYNITLTLVSYYR
PREEEALPHPLALTHKMGWLQLIGRMFVLIWATCISVKEGIAIFLLRPSDLQSILSDAW
FHFAFFIQAVLVILSVFLYLFAYKAYLACLVLAMALGWANMLYYTRGFQSMGMYSVMI
QKVILHDVLKFLFVYIVFLLGFGVALASLIEKCPDSKGECSSYGSFSDAVLELFKLTIGLG
DLNIQQNSKYPILFLFLLITYVILTFVLLLNMLIALMGETVENISKESERIWRLQRARTIL
EFEKLLPEWLRCKFRMGELCKVAQEDFRLCLRINEVKWTEWKTHVSFISEDPGPGRS
LDLSKAQDSSRYNSKTTLNAFDELDDVPETSV---

>TRPV3_Tursiops_truncatus_fragment

MDAHPKEMVPLMGRIATMPSGNAAILQEKRLAEITPTKSAHFFLEIEGFEPSTTVTKT
SPLIFSKPMDSNIRQCSLPGSCDDMDSPHSPQDDVTETPFNPNSPSANPAKQEQRHKQ
KRLKKHIFAAVSEGCVQQLLELLMELQELCEQRHSLEVPDFLMRNLTALDTGKTCLM
KALLNINPSTKEIVRILLAFAEENDILDRFINAKYTEEAFEGQTALNIAIERRQGDIAAVL
IEAGADVNAHAKGVFFNPKYQHEGFYFGETPLALAACTNQPEIVELLMGNEQTDITSQ
DSRGNNILHALVTVAEDFKTQNDFVKHMYDMILLRSGNWELETMRNNNGLTPLQLA
AKMGKAEILKYILSREIKEKRLRSLSRKFTDWAYGPVSSSLYDLTNVDTMTDNSVLKII
VYNTNIDNRHEMLTLEPLHTLLHMKWKKFAKYMFFLSFCSYFFYNITLTIISYYHTRG

EEALKHPLANKMGWLQLLGRMFVLIWAMCISVKEGIAIFLLRPSDMQSILSDAWFHF
VFFVQALLVILSVLLYLFACKEYLTCLVLAMALGWANMLYYTRGFQSMGMYSVMIQK
VILHDVLKFLFVYIVFLFGFGVALASLIEKCSKDNEDCTSYGSFSDTVLELFKLTIGLGDL
KIQQNSKYPILSLILLITYVTLTFVLLLNMLIALMGETVENISKESERIWRLQRARTILEF
EKMLPEWLKSRFQMGELCKVAEQDFRLCLRINEVKWTEWKTHVSFLNEDPGPGRRS

>TRPV3_Ornithorhynchus_anatinus

MNKTQKELIPLMGKKTNPPSSASAGLLEKKPSEVTPTKKSSHFFLEIEGFEPNPAVSKP
SPPIFSKPMDSNIRQCISGNCEDMDSPQSPQDDITETPSNPNSPCVNLAREQNRNKKKL
KKCIFAAVSEGNVEELNSLLVELKELSKRRRNMDIQDYLMHKFTASDTGKTCLMKALL
NINPNTKEIVRHLLSFAEENGILERFINAEYTEEAYKGQTALNIAIERRQCEITETLIEKG
ADVNVQAKGLFFNPKYKHEGFYFGETPLALAACTNQPEIVQMLMDNNKTDIASQDSR
GNTILHALVTVAEDFKTQNDFVKQMYDRILLRSKNMALETMQNNDGLTPLQLAAKM
GKSEILKYILSREIKEKPKRSLSRKFTDWAYGPVSSSLYDLTKVDTTTDNSVLEIIVYNT
NIDNRHEMLTLEPLHTLLRMKWKKFAKYMFFMSFCLYFFYNITLTLVSYYRPREEEA
LPHPLALTHKMGWLQLLGRMFVMIWATCITVKEGIAIFLLRPSDLQSILSDAWFHFAF
FIQAVLVILSVFLYLFAYKEYLACLVLAMALGWANMLYYTRGFQSMGMYSVMIQKVIL
HDVLKFLFVYIVFLLGFGVGPALASLIEKCPQNDSECSSYGSFSDAVLELFKLTIGLGDLD
IQQNSKYPILFLFLLITYVILTFVLLLNMLIALMGETVENISKESERIWRLQRARTILEFE
KLLPEWLRSKFRLGELCKVADNDFRLCLRINEVKWTEWKTHVSFISEDPGPGRHSEG
FNKIQDVSRSNSKTTLNAFDEIEDFPETSV---

>TRPV3_Bos_taurus

MKAHPKEMVPLTGRRATIPFVNPAIMQEKRPSEITPTKKSAHFFLEIEGFEPNPTVAK
TSPPIFSKPMDSNIRQCVSGNCDDMDSPQSPQDDVTETPSNPNSPSANLAKEEQRRKK
KRLKKRIFTAVSEGCVEELLELLGELQELCKRRHSLDVPDFLMHKLTALDTGKTCLMK
ALLNINPNTKEIVRILLAFAEENDILDRFINAEYTEEAYEGQTALNIAIERRQGDITAALI
AAGADVNAHAKGVFFNPKYQHEGFYFGETPLALAACTNQPEIVQMLMENEQTDITSQ
DSRGNNILHALVTVAEDFKTQNDFVKRMYDMILLRSRTWELETTRNNDGLTPLQLAA
KMGKAEILKYILSREIKDKRLRSLSRKFTDWAYGPVSSSLYDLTNVDTTTDNSVLEIIVY
NTNIDNRHEMLTLEPLHTLLHMKWKKFAKYMFFLSFCFYFFYNITLTLVSYYRPREEE
ALPHPLALTHKMGWLQLLGRMFVLIWAMFISVKEQGIAIFLLRPSDLQSILSDAWFHF
VFFAQAVLVILSVFLYLFAYKEYLACLVLAMALGWANMLYYTRGFQSMGMYSVMIQK
VILHDVLKFLFVYIVFLLGFGVALASLIEKCPKSHENCSSYGSFSDAVLELFKLTIGLGDL
NIQQNSKYPILFLFLLITYVILTFVLLLNMLIALMGETVENVSKESERIWRLQRARTILEF
EKILPEWLRSRFRMGELCKVAEDDFRLCLRINEVKWTEWKTHVSFLNEDPGPGRRTA
DSNKIQDSSRSNSKTTLNAFEEIDEFPETSV---

>TRPV3_Canis_familiaris

MNAHPKEMVPLMGRRAAVPSGNPAILQEKRPAEITPTKKSAHFFLEIEGFDPNPTVSK
TSPPIFSKPMDSNIRQCLSGNCDDMDSPQSPQDDVTETPSNPNSPSANVAKEEQRRKK
KRLKKRIFAAVSEGCVGELLELLVELQELCKRRRGLDVADFLMHKLTASDTGKTCLMK
ALLNINTKTKEIVRILLAFAEENDILDRFVNAAYTEEAYEGQTALNIAIERRQGDLTALL
IAAGADVNAHARGVFFNPKYQHEGFYFGETPLALAACTNQPEIVQLLMENEQTDITSQ
DSRGNNILHALVTVAEDFKTQNDFVKRMYDMILLRSGTWELETMHNNDGLTPLQLA
AKMGKAEILKYILSREIKEKPLRSLSRKFTDWAYGPVSSSLYDLTNVDTTTDNSVLEIIV
YNTNIDNRHEMLTLEPLHTLLHMKWKKFAKYMFFLSFCLYFFYNITLTLVSYYRPREE
EALPHPLALTHKMGWLQLLGRMFVLIWAMCISVKEGIAIFLLRPSDLQSILSDAWFHF
VFFVQAVLVVLSVFLYLFAYKEYLACLVLAMALGWANMLYYTRGFQSMGMYSVMIQK
VILHDVLKFLFVYIVFLLGFGVALASLIEKCPSDNKDCSSYGSFSDAVLELFKLTIGLGDL
NIQQNSKYPILFLFLLITYVILTFVLLLNMLIALMGETVENVSKESERIWRLQRARTILEF
EKMLPEWLRSRFRMGELCKVAEEDFRLCLRINEVKWTEWKTHVSFLNEDPGPGRSW
TDFNKIQDSSRSNSKTTLNAFDEIDEFPETSV--

>TRPV3_Ailuropoda_melanoleuca

MNAHPKEMVPLMGRRAAVPSGNPAVLQEKRSAEVTPTKKSAHFFLEIEGFDPNPTVS
KTSPPVFSKPMDSNIRQCLPGNCDDMDSPQSPQDDVTETPSNPNSPSANLAKEEQRR
RKKRLKKRIFAAVSEGCGEELLELLLELQELCKRRRGLDVADFLMHKLTASDTGKTCL
MKALLNINPNTKEIVRVLLAFAEDNDILDRFVNAAYTEEAYEGQTALNIAIERRQGDIA
ALLIAAGADVNAHARGVFFNPKYLHEGFYFGETPLALAACTNQPEIVQLLMENEQTDI
TSQDSRGNNILHALVTVAEDFKTQNDFVKRMYDTILLRSGTWELETMQNNDGLTPLQ
LAAKMGKAEILKYILGREIKEKPLRSLSRKFTDWAYGPVSSSLYDLTNVDTTTDNSVLE
IIVYNTNIDNRHEMLTLEPLHTLLHMKWTRFAKYMFFLSFCFYFFYNITLTLVSYYRPR
EEEALPHPLALTHKMGWLQLLGRMFVLIWATCISVKEGIAIFLLRPSDLQSILSDAWF
HFVFFVQAVLVILSVFLYLFAYKEYLACLVLAMALGWANMLYYTRGFQSMGMYSVMI
QKVILHDVLKFLFVYIVFLLGFGVALASLIEKCPSDNKDCSSYGSFSDAVLELFKLTIGLG
DLNIQQNSKYPILFLFLLITYVILTFVLLLNMLIALMGETVENVSKESERIWRLQRARTI
LEFEKMLPEWLRSRFRMGELCKVAEEDFRLCLRINEVKWTEWKTHVSFLNEDPGPV
RRTADFNKIQDSSRSNSKTTLNAFDEIDEFPETSV--

>TRPV3_Loxodonta_africana

MNAHPKEMVPLMGRKAMAPSGNPAVLQEKRPAELTPTKKSAHFFLEIEGFEPNPTIS
KTSPPIFSKPMDSNIRQCISGNCEDMDSPQSPQDDVTETPSNPNSPSANLAKGEQRRK
KKRLKKRIFAAVSEGCVDELVELLVELRELCRRRSDLDVPGQLLMHKLTSDTGKTSLM
KALLNINPTKEIVRTLLFAEENDILDRFINAEYTEEAYEGQTALNIAIERRQGDIAAVLIA
AGADVNAHAKGVFFNPKYQHEGFYFGETPLALAACTNQPEIVELLMENEQTDIASQDS
RGNNILHALVTVAEDFKTQNDFVKRMYDMILLRSRNWELETTRNNEGLTPLQLAAK
MGKAEVLNYILSREIKEKPLRSLSRKFTDWAYGPVSSSLYDLTNVDTMTDNSVLEIIVY
NTNIDNRPEMLTLEPLHTLLHMKWKKFAKYMFFLSFCFYFFYNITLTLVSYYRPREEE
ALPHPLALTHKMGWLQLLGRMFVLIWAMCISVKEGIAIFFLRPSDLQSILSDAWFHFV
FFIQAVLVILSVFLYLFAYKEYLACLVLAMALGWANMLYYTRGFQSMGMYSVMIQKVI
LHDVLKFLFVYILFLLGFGVALASLIEKCSKDNSNCSSYGSFGDAVLELFKLTIGLGDLNI

QQNSKYPILFLFLLITYVILTFVLLLNMLIALMGETVENVSKESERIWRLQRARTILEFE
KMLPEWLRSRFRMGELCKVAENDFRLCLRINEVKWTEWKTHVSFLNEDPGPVKRTA
DLNKIQNLSRSNSKTTLNAFDEIDEFPETSV--

>TRPV3_Sus_scrofa

MNAHPKEMVPLVGKRAVIPSGNPAILQEKRPAEITPTKKSAHFFLEIEGFEPNPTVAKT
SPPIFSKPMDSNIRQCISGNCDDMDSPQSPQDDVTETPSNPNSPSSAHLAKEEQRRKK
KRLKKRIFAAVSEGCVEELVELLMELQELGKRRRGLDVSDFLMHKLTASDTGKTCLM
KALLNINPNTKEVVRILLAFAEENDILDRFINAEYTEEAYRGQTALNIAIERRQGDITAL
LIHAGADVNAHAKGVFFNPKYQHEGFYFGETPLALAACTNQPEIVQLLMEHEQTDITS
QDSRGNNILHALVTVAEDFKTQNDFVKRMYDMILLRSGTWGLETTRNKDGLTPLQLA
AKMGKAEILKYILSREIKDKRLRSLSRKFTDWAYGPVSSSLYDLTNVDTTTENSVLEIIV
YNTNIDNRHEMLTLEPLHTLLHMKWKKFAKYMFFLSFCFYFFYNITLTLVSYYRPREV
EALPHPLALTHKMGWLQLLGRMFVLIWAMCISVKEGIAIFLLRPSDLQSILSDAWFHF
VFFVQAVLVILSVFLYLFAYKEYLACLVLAMALGWANMLYYTRGFQSMGMYSVMIQK
VILHDVLKFLFVYIVFLLGFGVALASLIEKCSKDNKDCTSYGSFSDAVLELFKLTIGLGDL
NIQQNSKYPILFLFLLITYVILTFVLLLNMLIALMGETVEDVSKESERIWRLQRARTILEF
EKMLPEWLRSRFRMGELCKVAEEDFRLCLRINEVKWTEWKTHVSFLNEDPGPGRRT
ADFNKIQDSSRSNSKTTLNAFDEMEEFPETSV--

>TRPV3_Myotis_lucifugus

MNAHPKEMVPLMGRKATVTSGNQVVLQEKRPAEITPTKKSKYLLSKCAAEEPRQSIS
DYPSAPLLQNPRDRKVRANVSADTAPTPLMPISGLVEPSSLSANLAKEEQRRKKKRLK
KCLFAAVSEGCVEDLVQLLVELQELCKRRRNLDVPDFLMHKLTASDTGKTCLMKALL
NINPNTKEIVRILLAFANENDILDRFINAEYTEEAYEGQTALNIAIERRQRDITALLIAAG
ADVNAHAKGVFFNPKYQHEGFYFGETPLALAACTNQPEIVQLLMENQQTDITSQDSR
GNNILHALVTVAEDFKTQNDFVKRMYDMILLRSGNWELETMRNNDGLTPLQLAAKM
GKAEILKYILSREIKEKRLRSLSRKFTDWAYGPVSSSLYDLTNVDTTTDNSVLEIIVYNT
NIDNRHEMLTLEPLHTLLRIKWKKFAKYMFFLSFFFYFFYNITLTLISYYRPREEEALP
HPLALTHKMGWLQLLGRMFVLIWAMCISVKEGIAIFLLRPSDLQSILSDAWFHFVFFV
QAVLVILSVFLYLFAYKEYLACLVLAMALGWANMLYYTRGFQSMGMYSVMIQKVILH
DVLKFLFVYIVFLLGFGGALASLIEKCPKDHKDCSSYGSFSDAVLELFKLTIGLGDLNIQQ
NSKYPILFLFLLITYVILTFVLLLNMLIALMGETVENVSKESERIWRLQRARTILEFEKM
LPEWLRSRFRTGELCKVAEEDFRLCLRINEVKWTEWKTHVSFLNEDPGSVRRTADFN
KIQDSSRSNSKTTLNAFEEIDEFPETSV---

>TRPV3_Gorilla_gorilla

MKAHPKEMVPLMGKRVAAPSGNPAVLPEKRPAEITPTKKSAHFFLEIEGFEPNPTVA
KTSPPVFSKPMDSNIRQWHCDRHGRCWRQVQRPVGSSGGEKERLSLRGCFSAQLAKE
EQRRKKRRLKKRIFAAVSEGCVEELVELLVELQELCRRRHDEDVPDFLMHKLTASDTG

KTCLMKALLNISPNTKEIVRILLAFAEENDILGRFINAEYTEEAYEGQTALNIAIERRQG
DIAAVLIAAGADVNAHAKGAFFNPKYQHEGFYFGETPLALAACTNQPEIVQLLMEHEQ
TDITSQDSRGNNILHALVTVAEDFKTQNDFVKRMYDMILLRSGNWELETTRNNDGLT
PLQLAAKMGKAEILKYILSREIREKRLRSLSRKFTDWAYGPVSSSLYDLTNVDTTTDNS
VLEITVYNTNIDNRHEMLTLEPLHTLLHMKWKKFAKHMFFLSFCFYFFYNITLTLVSY
YRPREEEAVPHPLALTHKMGWLQLLGRMFVLIWAMCISVKEGIAIFLLRPSDLQSILSD
AWFHFVFFVQAVLVILSVFLYLFAYKEYLACLVLAMALGWANMLYYTRGFQSMGMYS
VMIQKVILHDVLKFLFVYIVFLLGFGVALASLIEKCPKDNKDCSSYGSFSDAVLELFKLTI
GLGDLNIQQNSKYPILFLFLLITYVILTFVLLLNMLIALMGETVENVSKESERIWRLQRA
RTILEFEKMLPEWLRSRFRMGELCKVAEDDFRLCLRINEVKWTEWKTHVSFLNEDPG
PVRRTADFNKIQDSSRNNSKTTLNAFEEIEEFPETSV-------------------------------------
----

>TRPV3_Dipodomys_ordii_fragment

MNTHPKEMVPLISRRTTAPCGNSVVLSEKRPAELTPTKKSAHFFLEIEGFEPNPTVTK
TSPPIFSKPMDSNIRQCLSGNCDDMDSPQSPQDDVTETPSNPNSPSANLAKEEQRRKK
KRLKKCIFTAVSEGCVEELVELLLELQELCKRRRGRDVPDFLMHKLTASDTGKTCLMK
ALLNINPNTKEIVRILLAFAEENDILDRFINAEYTEEAYEGQTALNIAIERRQRDIAAELI
AAGADVNAHAKGAFFNPRHQHDGFYIRETPLALAACTNQPEIVQLLMENEQTDITSQ
DSRGNNILHALVTVAEDFKTQNDFVKRMYDMILLRSGNWELETMCNHDGLTPLQLA
AKMGKAEILKYILSREIKEKPLRSLSRKFTDWAYGPVSSSLYDLTNVDTTADNSVLEIIV
YNTNIDNRHEMLTLEPLHTLLHMKWKKFAKYMFFLSFCFYFFYNITLTLVSYYRPREE
EALPHPLALTHKMGWLQLLGRMFVLIWATCISVKEGIAIFLLRPSDLQSILSDAWFHF
VFFVQAVLVILSVFLYLFAYKEYLACLVLAMALGWANMLYYTRGFQSMGMYSVMIQK
LASLIEKCSKDKTDCSSYGSFSDAVLELFKLTIGLGDLNIQQNSTYPILFLFLLITYVILTF
VLLLNMLIALMGETVENVSKESERIWRLQRARTILEFEKMLPEWLRSRFRMGELCKV
AEEDFRLCLRINEVKWTEWKTHVSFLNEDPGPIRRTVDFNKIQDSSRSNSKTTLNAFE
EIDEFPETSV--

>TRPV3_Otolemur_garnettii

MNAHPREMVPLIGRRVAAPSGNPAVLPEKRPVEVTPTKKSAHFFLEIEGFEPNPTVTK
TSPPIFSKPMDSNIRQCLSGNCDDMDSPQSPQDDMTETPSNPNSPSLSANLAKEEQRR
KKKRLKKRIFSAVSEGCVEELLELLGELQELCKRRRGLDVPDFLMHKLTASDTGKTCL
MKALLNINPSTKEIVRILLAFAEENDILDRFINAEYTEEAYEGQTALNIAIERRQGDITA
VLIAAGADVNAHAKGVFFNPKYQHEGFYFGETPLALAACTNQPEIVQLLMEYEQTNIA
SQDSRGNNILHALVTVAEDFKTQNDFVKRMYDMILLRSGNWELETVRNNDGLTPLQL
AAKMGKAEILKYILSREIKEKPLRSLSRKFTDWAYGPVSSSLYDLTNVDTTTDNSVLEII
VYNTNIDNRHEMLTLEPLHTLLHMKWKKFAKYMFFLSFCFYFFYNITLTLVSYYRPRE
EEALPHPLALTHKMGWLQLLGRMFVLIWAMCISVKEGIAIFLLRPSDLQSILSDAWFH
FVFFIQAVLVILSVFLYLFAYKEYLACLVLAMALGWANMLYYTRGFQSMGMYSVMIQK
VILHDVLKFLFVYIVFLLGFGVALASLIEKCPNDNKDCSSYGSFSEAVLELFKLTIGLGDL
NIQQNSKYPVLFLFLLITYVILTFVLLLNMLIALMGETVENVSKESERIWRLQRARTILE
FEKMLPEWLRSRFRMGELCKVAEEDFRLCLRINEVKWTEWKTHVSFLNEDPGPVKR
TADFNRIQDSSRSNSKTTLNAFDEIDEFPETSV---

>TRPV3_Oryctolagus_cuniculus

MNVLPKDMVPLMGRRATIAGGNPAVLLEKRPAEVTPTKKSAHFFLEIEGFESNPTVT
KTSPPIFSKPMDSNIRPCVSGNCDDMDSPQSPQDDVTETPSNPNSPSANLAKEEKRRR
KHRLKKRIFTAVSEGCVQELVGLLLELQELCRRHRGLDVSDFLMHKLTASDTGKTCLM
KALLNINPRTKEIVRILLTFAEENGILDRFINAEYTEEAYEGQTALNIAIERRQRDIAALL
IAAGADVNAHAKGVFFNPKYQHEGFYFGGCDGDRVSCPLALRLVPLWESKTRDAPGR
KRNGNDLWNSLGQIPSVLKGEGDGPCGLREXILLRSGSWQLETMNHDGLTPLQLAAK
MGKAEILKYILGREIKEKPLRGLSRKFTDWAYGPVSSSLYDLTNVDTTTDNSVLEIIVY
NTNIDNRHEMLTLEPLHTLLHMKWKKFAKYMFFLSFCFYFFYNITLTLVSYYRPREEE
ALPHPLALTHNMGWLQLLGRMFVLIWATCISVKEGIAIFLLRPSDLQSILSDAWFHFV
FFVQAVLVILSVFLYLFAYKEYLACLVLAMALGWANMLYYTRGFQSMGMYSVMIQKVI
LHDVLKFLFVYIVFLLGFGVALASLIEEVLRRATRSGSSYGSFGTAVLSSQAQQSGLGDL
NIQAEPPSTPSCFCSWLINYVISLRLPTSNAHRLMGENVENVSKETKRICCWKLGRART
ILEFEKMLPEWLRSRFRMGELCKVAEEDFRLCLRINEVKWTEWKTHVSFLKEDPGPI
KRTADLNKIQDSSRSNSKTTLNAFEEMEEFPETSV--

>TRPV3_Macaca_fascicularis

MNAHPKETVPLMGKRVAAPSGNPAVLPEKRAAEITPTKKSAHFFLEIEGFEPNPVAAK
TSPPVFSKPMDSNIRQCISGNCDDMDSPQSPQDDVTETPSNPNSPGAHLAKEEQRRKK
RRLKKRIFAAVSEGCVEELVELLVELQELCRRRHDEDVPDFLMHKLTASDTGKTCLMK
ALLNINPNTKEIVRVLLAFAEENDILGRFINAEYTEEAYEGQTALNIAVERRQGDIAAAL
IAAGADVNAHAKGAFFNPKYQHEGFYFGETPLALAACTNQPEIVQLLMDHEQTDITSQ
DSRGNNILHALVTVAEDFKTQNDFVKRMYDMILLRSGNWELETTRNNDGLTPLQLA
AKMGKAEILKYILSREIKEKRLRSLSRKFTDWAYGPVSSSLYDLTNVDTTTDNSVLEIT
VYNTNIDNRHEMLTLEPLHTLLRMKWKKFAKYMFFLSFCFYFFYNITLTLVSYYRPRE
EEALPHPLALTHKMGWLQLLGRMFVLIWATCISVKEGIAIFLLRPSDLQSILSDAWFHF
VFFIQAVLVILSVFLYLFAYKEYLACLVLAMALGWANMLYYTRGFQSMGMYSVMIQKV
ILHDVLKFLFVYIVFLLGFGVALASLIEKCPKDNKDCSSYGSFSDAVLELFKLTIGLGDLN
IQQNSKYPILFLFLLITYVILTFVLLLNMLIALMGETVENVSKESERIWRLQRARTILEFE
KMLPEWLRSRFRMGELCRVAEEDFRLCLRINEVKWTEWKTHVSFLNEDPGPVRRTA
DFNKIQDFSRNNSKTTLNAFEEIEDFPETSV---

>TRPV3_Cavia_porcellus

MNAHPKEMVPLMGRKANVSCGNPAVLTEKRPAEVTPTKKSAHFFLEIEGFEPNPTVT
KTSPPVFSKPMDSNIRQCLSGNCDDMDSPQSPQDDVTETPSNPNSPRHVIARRQEQES
DRESRARRIFSAVSEGLELCRRRRGLDVSDFLMHKLTASDTGKTCLMKALLNINPNTK
EIVHILLTFADENDILDQFVNAEYTEEAYEGQTALNIAIERRQRDIAAVLIAAGADVNA
HAKGVFFNPKYQHEGFYFGETPLALAACTNQPEIVQLLMENEQTDITSQDSRGNNILH
ALVTVAEDFKTQNDFVKRMYDMILLKSGNWELETMHNNDGLTPLQLAAKMGKAEIL

KYILSREIKEKPLRSLSRKFTDWAYGPVSSSLYDLTNVDTTTDNSVLEIIVYNTNIDNRH
EMLTLEPLHTLLRMKWKKFAKYMFFLSFFFYFFYNITLTLVSYYRPREKEDLPHPLAL
SHKMGWLQLLGRMFVLIWATCISVKEGIAIFLLRPSDLQSILSDAWFHFVFFIQAVLVIL
SVFLYLFAYKEYLAFLVLAMALGWANMLYYTRGFQSMGMYSVMIQKVILHDVLKFLF
VYIVFLLGFGVALASLIEKCSDGNKDCSSYGSFSDAVLELFKLTIGLGDLKIQQNSTYPIL
FLFLLLNMLIALMGETVENVSKESERIWRLQRARTILEFEKMLPEWLRSRFRMGELCK
VAEDDFRLCLRINEVKWTEWKTHVSFLNEDPGPIRRTVDFNKIQDSSRSNSKTTLNAF
EEIDEFPETSV--

>TRPV3_Spermophilus_tridecemlineatus

MHAHPKEMVPLVGRRTTGPSGNPVVLTEKRPAEVTPTKKSAHFFLEIEGFEPNPTVA
KTSPPIFSKPMDSNIRQCISGNCDDMDSPQSPQDDVTETPSNPNSPSANLAKEEQRRK
KKRLKKRIFAAVSEGCVEELVELLAELQELCKRRRGGMDVPDFLMHKLTASDTGKTCL
MKALLNINPNTKEIVRILLAFAEENDILDRFINAEYTEEAYEGQTALNIAIERRQGDITA
VLIAAGADVNAHAKGVFFNPKYQHEGFYFGETPLALAACTNQPEIVQLLMENEQTDIT
SQDSRGNNILHALVTVAEDFKTQNDFVKRMYDMILLRSGTWELETTRNNDGLTPLQL
AAKMGKAEILKYILSREIKEKPLRSLSRKFTDWAYGPVSSSLYDLTNVDTTTDNSVLEI
TVYNTNIDNRHEMLTLEPLHTLLHMKWKKFAKYMFFLSFCFYFFYNITLTLVSYYRPR
EVEALPHPLALTHKMGWLQLLGRMFVLIWATCISVKEGIAIFLLRPSDLQSILSDAWF
HFVFFIQAMLVILSVFLYLFAYKEYLACLVLAMALGWANMLYYTRGFQSMGMYSVMI
QKVILHDVLKFLFVYIVFLLGFGVALASLIEKCSEDNKDCSSYGSFSDAVLELFKLTIGLG
DLNIQQNSTYPILFLFLLITYVILTFVLLLNMLIALMGETVENVSKESERIWRLQRARTI
LEFEKMLPEWLRSRFRMGELCKVAEGDFRLCLRINEVKWTEWKTHVSFLNEDPGPIK
RTADINKMQDSSRNNSKTTLNAFDEMDEFPETSV---------------------------------------
----

>TRPV3_Equus_caballus

MNAHPKEMMPLMGRRATIPGAHPAILQEKRPAELTPTKKSAHFFLEIEGFEPNPTVT
KTSPPVFSKPMDSNIRQCVSGNCDDMDSPQSPQDDVTETPSNPNSPSANLAKEERRR
KKRRLKKRIFGAVSEGCVEELLELLAELLEICKRRRSVDVPDFLMHKLTASDTGKTCL
MKALLNINPNTKEIVRILLAFAEENDILDRFINAAYTEEAYEGQTALNIAIERRQGDITA
ALIAAGADVNAHAKGVFFNPKYQHEGFYFGETPLALAACTNQPEIVQLLMENEQTDIT
SQDSQGNNILHALVTVAEDFKTQNDFVKRMYDMILLRSGNWELETTRNKDGLTPPQL
AAKLGKAEILKYILSREIREKPLRSLSRKFTDWAYGPVSSSLYDLTNVDTTTDNSVLEII
VYNTTIDNRHEMLTLEPLHTLLHMKWKKFAKYMFFLSFCFYFFYNITLTLVSYYRPRE
EEALPHPLALTHKMGWLQLLGRMFVLIWAMCISVKEGIAIFLLRPSDLQSILSDAWFH
FVFFVQAVLVILSVFLYLFAYKEYLACLVLAMALGWANMLYYTRGFQSMGMYSVMIQ
KVILHDVLKFLFVYIVFLLGFGVALASLIEKCSKDNKNCSSYGSFSDAVLELFKLTIGLGD
LNIQQNSKYPILFLFLLITYVILTFVLLLNMLIALMGETVENISKESERIWRLQRARTILE
FEKMLPEWLRSRFQMGELCKVAEEDFRLCLRINEVKWTEWKTHVSFLNEDPGPGRR
TDFNKIQDSSRSNSKTTLNAFDEIEEFPETSV---

>TRPV3_Mus_musculus

MNAHSKEMAPLMGKRTTAPGGNPVVLTEKRPADLTPTKKSAHFFLEIEGFEPNPTVT
KTSPPIFSKPMDSNIRQCLSGNCDDMDSPQSPQDDVTETPSNPNSPSANLAKEEQRQK
KKRLKKRIFAAVSEGCVEELRELLQDLQDLCRRRRGLDVPDFLMHKLTASDTGKTCL
MKALLNINPNTKEIVRILLAFAEENDILDRFINAEYTEEAYEGQTALNIAIERRQGDITA
VLIAAGADVNAHAKGVFFNPKYQHEGFYFGETPLALAACTNQPEIVQLLMENEQTDIT
SQDSRGNNILHALVTVAEDFKTQNDFVKRMYDMILLRSGNWELETMRNNDGLTPLQ
LAAKMGKAEILKYILSREIKEKPLRSLSRKFTDWAYGPVSSSLYDLTNVDTTTDNSVLE
IIVYNTNIDNRHEMLTLEPLHTLLHTKWKKFAKYMFFLSFCFYFFYNITLTLVSYYRPR
EDEDLPHPLALTHKMSWLQLLGRMFVLIWATCISVKEGIAIFLLRPSDLQSILSDAWF
HFVFFVQAVLVILSVFLYLFAYKEYLACLVLAMALGWANMLYYTRGFQSMGMYSVMI
QKVILHDVLKFLFVYILFLLGFGVALASLIEKCSKDKKDCSSYGSFSDAVLELFKLTIGLG
DLNIQQNSTYPILFLFLLITYVILTFVLLLNMLIALMGETVENVSKESERIWRLQRARTI
LEFEKMLPEWLRSRFRMGELCKVADEDFRLCLRINEVKWTEWKTHVSFLNEDPGPIR
RTADLNKIQDSSRSNSKTTLYAFDELDEFPETSV--

>TRPV3_Gallus_gallus

MIKDNKEVVPLMGKKTNPPGAPPSNQQEKKPTESTPTKKSSHFFLEIEGFESNATPNN
TSPPVFSKPMDSNIRPCASANGEDMDSPQSLQDDVTEYSPNVDSCGANIAQGPEQTGA
RKKLKKYIFRAVSEGNVEELQGLLAELKERSNVCTNMTVPDYLMKKFTASDTGKTCL
MKALLNINNNTNQIVNMLLSFAEENGILERFINAAYTEEAYRGQTALNIAIERRQFEIT
QTLIEKGADVNAHAQGIFFNPKHKHEGFYFGETALALAACTNQPDIIELLMDNTRTNI
AAQDSRGNNILHALVTVAEDFKTQNDFVIRMYDMILLKSKDRNLEKVKNKEGLTPLQ
LAAKTGKLEVLKYILSREIRDKPNRSLSRKFTDWAYGPVQSSLYDLTELDTTADNSVLE
IIVYNTNIGNRHEMLTLEPLNSLLRMKWKRFARHMLFMSCCFYFLYNVTLTLVSYHRP
NENEAPPYPLALTRGVGWLQLSGQVMVMLGAIFLAIKESVAIFLLRPSDLQSILSDAWF
HFAFFIQAMLVIFSVFLYLSSYKEHLVCLVLAMALGWANMLYFTRGFQSMGIYSVMIQ
KVILNDVIKFLVVYIVFLLGFGVALAALIETCQNGGECLSNSSLGPVLMDLFKLTLGLGD
LEIQQNSKYPVLFLLLLITYVVLTFVLLLNMLIALMGETVEDISKESEHIWKLQRARTIL
EFEKFLPKSLRKKFQLGERCKVAENDTRVCLRINEVRWTEWKTHVSFINEDPGPTVH
FLADPSKVQDNSRTNSKNTLNTFEETDDLPETSL--

>TRPV3_Xenopus_tropicalis

MINKHPGEMSPLISRERLAPGTPPRSPRHTENGLKRNDTTDFSRSNPLWLSTQMDTN
NYSNEKKNEDTDTPQTTQTAFSPCYGWQGRKPALTRNTRKPTKKLLFKAVSEGDTE
MLTDLLSEAKSSSRVYSVTQAKEFFMHKLTSKDTGKTCLMKALLNINEKTPEVVRSLL
TFAEENDILETFINAEYTEENYKGQTALNIAIERRQVELVKYLIEKGAKIDVRAQGRFFN
PKNKYEGFYFGETPLALAACTNQPEIVQLIMDKSPTIGTIQDSLGNTVLHALVNVADNS
EAQNDFIIGMYDTILRNCKNKSLEQIPNNEGLTSMQLAAKLGKTEILHYILSREIKEKEN
MVLSRKFTDWAYGPVSSSLYDLTSIDTCWPNSVLEIVVYNTDIDNRHELLTLEPLHTL
LQMKWKKFARYMFFLSFLLSFTYNIALTLVSYYRPRGEQDVYPLNLSYENGWLQLVG
QMFIIVCATYLMVKEAVVMFLVKQSDLKSVLSDAWFHILFFIQAVLVIVSVFCYLFGVD

FYLVFLVLAMALGWMNLLYYTRGFQSLGIYSVMIQKVILNDVLKFLFVYILFLLGFGVA
LASLLENCEDGEECQSLSTAILELFELTIGLRGLEMDKDPKYPVLFLFLLITFVILTFVLL
LNMLIALMGETVEKISQESEHIWRLQRARTILEFEKSLPAWLQARFQLGESCTVSKGD
NNRICLRINEVKWTEWNNHVTCIKEEPGLTFFPDTKDTNTASEDELDSFEKFAGISISV
GDLEDQAMETTV--

>TRPV3_Anolis_carolinensis_fragment

PYPLAMTDNLGWLQLTGQVVVMLGAIFIAIKESVAIFLLRPSDLQSILSDAWFHFAFFI
QAVLVIFSVCLYLLSYKEHLVCLVLAMSSGWANMLYFTRGLQSMGIYSVMIQQVILNDV
IKFMAVYIVFLLGFGVALAALIETCPEDSKCSNYSTLGSVEVELFRLTLGLGDLDINENA
KYPILFLLLLISYVVLTFVLLLNMLIALMGETVEDISKESEHIWRLQRARTIVETEKLLP
KFLRKKFQLGEWCKVADNDTRLCLRINEVKWTEWKTHVSFINEDPGPTVKIRFLSDY
NRVQESSRSNSKTTLNAYEEVYDLPETSV---

>TRPV3_Meleagris_gallopavo_fragment

SSHFFLEIEGFEGNATPNNTSPPVFSKPMDSNIRPCASANGEDMDSPQSLQDDVTEYS
PNVDSCCANIAQGPEQTRAPKKLKKYLFRAVSEGNIEELQGLLAELKERSNVCRNTTV
PDYLMKKFTASDTGKTCLMKALLNINNNTNEIVNILLSFAEENGILERFINAAYTEEAY
KGQTALNIAIERRQFEITQTLIEKGADVNAHAQGIFFNPKYKHEGFYFGETALALAACT
NQPDIIELLMDNTRTNIASQDSRGNNILHALVTVAEDFKTQNDFVIRMYDMILLKSKD
RNLEKVKNKEGLTPLQLAAKTGKLEVLKYILSREIRDKPNRSLSRKFTDWAYGPVQSS
LYDLTELDTTADNSVLEIIVYNTNIGNRHEMLTLEPLNSLLRMKWKRFARHMLFMSC
CFYFLYNVTLTLVSYHRPNENEAPPYPLALTRGVGWLQLSGQVMVMLGAIFLAIKESV
AIFLLRPSDLQSILSDAWFHFAFFIQAMLVIFSVFLYLFSYKEHLVCLVLAMALWANML
YFTRGFQSMGIYSVMIQKVILNDVIKFLVVYIVFLLGFGVALAALIETCQNGGECHSNSS
LGPVLMDLFKLTLGLGDLEIQQNSKYPVLFLLLLITYVVLTFVLLLNMLIALMGETVEDI
SKESEHIWKLQRARTILEFEKFLPKSLRKKFQLGERCKVAENDTRVCLRINEVRWTEW
KTHVSFINEDPGPTVHFLADPSKVQDNSRTNSKNTLNTFEEMDDLPETSL-------------
----

>TRPV3_Pelodiscus_sinensis_fragment

KDKKENIPLMGKRTNTLGPPPTNNQEKKPAESTPTKKSSQFFLEIEGFESNSSLRQTSP
PVFSKPMDSNIRPCASRNGEDMDSPQSLQDDITEYATNVDSYCTHMLQGDHQNNAPK
KLKKYMFRAVSEGNTEELQHLLMEVKERSNRCRNMTVQDYLMKKLTDSDTGKTCLM
KALLNINQNTNEIVKMLLSFGEENSFLERLINAKYTEEAYKGQTALNIAIERRQYVIAH
TLIEKGADVNAHAQGVFFNPKRKHDGFYFGETPLALAACTNQPDIVQLLMDNRRTDI
TSQDSRGNNILHALVTVAEDFKTQNDFVIRMYDMILLKSKNRTLETMKNKDGLTPLQ

LAAKSGKLEILKYILSREIRDKPNRSLSRKFTDWAYGPVQSSLYDLTELDTTADNSLLEI
IVYNTNIGNRHEMLALEPLHSLLRMKWKKFARHMFFMSFCFYFIYNILLTLVSYYRPW
RGEAPPYPLALTHNLGWLQLLGQVLIIIEAIFLAIKESVAIFLLRPSDLQSILSDAWFHFA
FFIQAVLVIFSVFLYLFSYKEHLMCLVLAMALGWVNMLYFTRGFQSMGIYSVMIQKVIL
HDVLKFLVVYIVFLLGFSVALAALIEECPNDNKCHSYSSLGTVLMELFKLTIGLGDLEM
QQKSKYPVLFLLLLITYVLLAFVLLLNMLIALMGETVENISKESEHIWRLQRAKTILEFE
KILPKYLKKKFLLGEMCKVAENDTRVCLRINEVKWTEWKTHVSFINEDPGPTGYQAE
TGYNRIQDTSRSNSKNTLNTFDEMDYLIETTV--

>TRPV3_Procavia_capensis_fragment

MNAHSKEMVPLMGRKASVPSGNPAVLQEKKPAELTPTKKSAHFFLEIEGFEPNPTVP
KTSPPIFSKPMDSNIRQCTSDNCDDMDSPQSPQDDVTETPSNPNSPXXXXXXXXXXXXX
XXXXXXSRIFAAVSEGCVEELVELLRELQELCRRRRGLDMPDFLIHKLTASDTGKTCLM
KALLNINPNTKEIVRILLTFAEENNILDRFINAEYTEEAYEGQTALNIAIERRQGDVTAE
LIAAGANVNAHAKGVFFNPKYQHEGFYFGETPLALAACTNQPEIVQLLMENEQTDITS
QDSRGNNILHALVTVAEDFKTQNDFVKHMYDMILLKSRSWELETMRNNDSLTPLQL
AAKMGKAEILKYILSREIKEKPLRSLSRKFTDWAYGPVSSSLYDLTDVDTTTENSVLEII
VYNTNIDNRHEMLTLEPLHTLLHMKWKKFAKYMFFLSFCFYFFYNITLTLVSYYRPRE
EEALPHPLALTHQMGWLQLLGRMFVLIWAMCISVKE-----------------------------------
----

>TRPV3_Taeniopygia_guttata

MIKDNNEIVPLMGKKTNLPGNPPANQQEKKVTEGTPTKKSSHFFLEIDGFESNASPNN
TSPPVFSKPMDSNIRPCASANGEDMDSPQSLQDDATEYSPNVDSCGATTCQDPELRSA
RKKLKRNLFRAVSEGNAEELQRLLAELRERSGACTSLPMPDYLMKKFTASDTGKTCL
MKALLNINQNTNEIVNMLLSFAEENGILERFINAAYTEEAYKGQTALNIAIERRQYEIT
QSLIEKGADVNAHAQGIFFNPKHKHEGFYFGETALALAACTNQPDIIELLMDNARTNIS
SQDSRGNNILHALVTVAEDSKTQNDFVIRMYDMILLKSKDRTLETTKNKEGLTPLQLA
AKTGKLEILKYILSREIREKPNRSLSRKFTDWAYGPVQSSLYDLTELDTTADNSVLEIIV
YNTNIGNRHEMLTLEPLNSLLRMKWKKFARHMFFMSCCFYFLYNVTLTLVSYHRPN
TNEAPPYPLALTRGVGWLQLSGQVMVMLGAIFLAIKESVAIFLLRPSDLQSILSDAWFH
FAFFIQALLVIFSVFLYLFSYKEHLVCLVLAMALGWANMLYFTRGFQSMGIYSVMIQKA
TVILQDVIKFLVVYIVFLLGFGVALAALIETCQEGGECHSNSSLGPVLMDLFKLTLGLGD
LEIQQNSKYPVLFLLLLITFVVLTFVLLLNMLIALMGETVEDISKESEHIWKLQRARTIL
EFEKFLPKCLRKKFQLGERCKVAENDTRVCLRINEVKWTEWKTHVSFINEDPGPTER
VDPSKIQDNSRTNSKNTLNTFEETDDLPETSV--

>TRPV4_Rattus_norvegicus

MADPGDGPRAAPGDVAEPPGDESGTSGGEAFPLSSLANLFEGEEGSSSLSPVDASRPA
GPGDGRPNLRMKFQGAFRKGVPNPIDLLESTLYESSVVPGPKKAPMDSLFDYGTYRH
HPSDNKRWRRKVVEKQPQSPKAPAPQPPPILKVFNRPILFDIVSRGSTADLDGLLSYLL
THKKRLTDEEFREPSTGKTCLPKALLNLSNGRNDTIPVLLDIAERTGNMREFINSPFRD
IYYRGQTALHIAIERRCKHYVELLVAQGADVHAQARGRFFQPKDEGGYFYFGELPLSLA
ACTNQPHIVNYLTENPHKKADMRRQDSRGNTVLHALVAIADNTRENTKFVTKMYDL
LLLKCSRLFPDSNLETVLNNDGLSPLMMAAKTGKIGVFQHIIRREVTDEDTRHLSRKF
KDWAYGPVYSSLYDLSSLDTCGEEVSVLEILVYNSKIENRHEMLAVEPINELLRDKWR
KFGAVSFYINVVSYLCAMVIFTLTAYYQPLEGTPPYPYRTTVDYLRLAGEVITLLTGVLF
FFTSIKDLFMKKCPGVNSLFVDGSFQLLYFIYSVLVVVSAALYLAGIEAYLAVMVFALVL
GWMNALYFTRGLKLTGTYSIMIQKILFKDLFRFLLVYLLFMIGYASALVTLLNPCTNM
KVCNEDQSNCTVPSYPACRDSETFSAFLLDLFKLTIGMGDLEMLSSAKYPVVFILLLLVT
YIILTFVLLLNMLIALMGETVGQVSKESKHIWKLQWATTILDIERSFPVFLRKAFRSGE
MVTVGKSSDGTPDRRWCFRVDEVNWSHWNQNLGIINEDPGKSEIYQYYGFSHTMGR
LRRDRWSSVVPRVVELNKNSGTDEVVVPLDNLGNPNCDGHQQGYAPKWRAEDAPL--

>TRPV4_Homo_sapiens

MADSSEGPRAGPGEVAELPGDESGTPGGEAFPLSSLANLFEGEDGSLSPSPADASRPAG
PGDGRPNLRMKFQGAFRKGVPNPIDLLESTLYESSVVPGPKKAPMDSLFDYGTYRHHS
SDNKRWRKKIIEKQPQSPKAPAPQPPPILKVFNRPILFDIVSRGSTADLDGLLPFLLTH
KKRLTDEEFREPSTGKTCLPKALLNLSNGRNDTIPVLLDIAERTGNMREFINSPFRDIY
YRGQTALHIAIERRCKHYVELLVAQGADVHAQARGRFFQPKDEGGYFYFGELPLSLAA
CTNQPHIVNYLTENPHKKADMRRQDSRGNTVLHALVAIADNTRENTKFVTKMYDLL
LLKCARLFPDSNLEAVLNNDGLSPLMMAAKTGKIGIFQHIIRREVTDEDTRHLSRKFK
DWAYGPVYSSLYDLSSLDTCGEEASVLEILVYNSKIENRHEMLAVEPINELLRDKWRK
FGAVSFYINVVSYLCAMVIFTLTAYYQPLEGTPPYPYRTTVDYLRLAGEVITLFTGVLFF
FTNIKDLFMKKCPGVNSLFIDGSFQLLYFIYSVLVIVSAALYLAGIEAYLAVMVFALVLG
WMNALYFTRGLKLTGTYSIMIQKILFKDLFRFLLVYLLFMIGYASALVSLLNPCANMKV
CNEDQTNCTVPTYPSCRDSETFSTFLLDLFKLTIGMGDLEMLSSTKYPVVFIILLVTYIIL
TFVLLLNMLIALMGETVGQVSKESKHIWKLQWATTILDIERSFPVFLRKAFRSGEMVT
VGKSSDGTPDRRWCFRVDEVNWSHWNQNLGIINEDPGKNETYQYYGFSHTVGRLRR
DRWSSVVPRVVELNKNSNPDEVVVPLDSMGNPRCDGHQQGYPRKWRTDDAPL------

>TRPV4_Loxodonta_africana_fragment

KQPQSPKAPAPQPPPVLKVFNRPILFDIVSRGSTTDLDGLLPFLLTHKKRLTDEEFREP
STGKTCLPKALLNLSNGRNDTIPLLLDIAERTGNMREFINSPFRDVYYRGQTALHIAIE
RRCKHYVELLVAQGADVHAQARGRFFQPKDEGGYFYFGELPLSLGACTNQPHIVNYLT
EPHKKADMRRQDSRGNTVLHALVAIADNRENTKFVTKMYDLLLLKCARLFPSNLEAV
LNNDGLQPQMVAYNNNKIYVFQHIIRREVTDEDTRHLSRKFKDWAYGPVYSSLYDLSS
LDTCGEEASVLEILVYNSKIENRHEMLAVEPINELLRDKWRKFGAVSFYISVVSYLCAM
VIFTLTAYYQPLEGTPPYPYRTTVDYLRLAGEIITLFTGVLFFFTNIKDLFMKKCPGVNS
LFIDGSFQLLYFIYSVLVIVSAALYLAGIEAYLAVMVFALVLGWMNALYFTRGLKLTGTY
SIMIQKILFKDLFRFLLVYLLFMIGYASALVSLLNPCANMKVCNQDQTNCSVPTYPSCR
DSETFSTFLLDLFKLTIGMGDLEMLSSAKYPVVFIILLVTYIILTFVLLLNMLIALMGETV
GQVSKESKHIWKLQWATTILDIERSFPVFLRKAFRSGEMVTVGKSSDGTPDRRWCFR

VPSACWDAQNGSLCQSSAPRSGKMELWSLFYESFVVKRLPRARTDLSVPRVVELNKSS
NQDEVVVPLDTMGPSCDSHQQSYPPKYGGPTTPP---------------------------------------
----

>TRPV4_Bos_taurus

MADPGEGPRVGPGEMAESPGDESGPPGGEAFPLSSLANLFEGEDGSPSPSPADGGRST
GPGDGRPNLRMKFQGAFRKGVPNPIDLLESTLYESSVVPGPKKAPMDSLFDYGTYRH
HPSDNKRWRRKVIEKQPQSPKAPAPQPPPILKVFNRPILFDIVSRGSTADLDGLLPFLL
THKKRLTDEEFREPSTGKTCLPKALLNLSNGRNDTIPVLLDIAERTGNMREFINSPFRD
IYYRGQTALHIAIERRCKHYVELLVAQGADVHAQARGRFFQPKDEGGYFYFGELPLSLA
ACTNQPHIVNYLTENPHKKADMRRQDSRGNTVLHALVAIADNTRENTKFVTKMYDL
LLLKCARLFPDSNLEAVLNNDGLSPLMMAAKTGKIGIFQHIIRREVTDEDTRHLSRKFK
DWAYGPVYSSLYDLSSLDTCGEETSVLEILVYNSKIENRHEMLAVEPINELLRDKWRK
FGAVSFYINVVSYLCAMVIFTLTAYYQPLEGTPPYPYRTTMDYLRLAGEIITLFTGILFFF
TNIKDLFMKKCPGVNSLFIDGSFQLLYFIYSVLVIVSAALYLAGIEAYLAVMVFALVLGW
MNALYFTRGLKLTGTYSIMIQKILFKDLFRFLLVYLLFMIGYASALVSLLNPCANLKVCD
EDHANCTVPTYPSCRDSETFSTFLLDLFKLTIGMGDLEMLSSTKYPVVFIILLVTYIILTF
VLLLNMLIALMGETVGQVSKESKHIWKLQWATTILDIERSFPVFLRKAFRSGEMVTVG
KSSDGTPDRRWCFRVDEVNWSHWNQNLGIINEDPGKNENYQYYGFSHTVGRLRRDR
WSSVVPRVVELNKNSNPDEVVVPLDNVGNPSCDGHQQSYPPKWRTDDAPL----------

>TRPV4_Ornithorhynchus_anatinus_fragment

KETPSVKAPAPHPPPILKVFNRPILFDIVSRGSTADLDGLLSFLLTHKKRLTDEEFREPS
TGKTCLPKALLNLCNGRNDTIPLLLDIAERTGNTREFINSPFRDVYYRGQTALHIAIER
RCKHYVELLVALGADVHAQARGRFFQPKDEGGYFYFGKAGGGGARGLAGGQGRGETE
GWHGTRGDRGGXKFVTKMYDLLLVQCKKQFPDRHLETVLNNDGLSPLMMAAKTGKI
GVFQHIIRREVKDEEVRHLSRKFKDWAYGPVYSSLYDLSSLDTCGEEASVLEILVYNSKI
EVSARATRSTRRWSWPGSRGTRGASPTDGVGSKPRIASNCSFIYSVLVMVSALYLGGV
EAYEAVMVFAVLGWMNALYFTRGLKLTGTYSIMIQKILFKDLFRFLLVYLLFMIGYASA
LVSLLNPCPGREACPRGQSNCTVPTYPSCRDSETFSVFLLDLFKLTIGMGDLEMLGSTK
YPVVFVILLVTYIILTFVLLLNMLIALMGETVGQVSKQSKHIWKLQ----------------------
----

>TRPV4_Oryctolagus_cuniculus_fragment

EPSTGKTCLPKALLNLSNGRNDTIPALLDIAERTGNMREFINAPFRDIYYRGQTALHIAI
ERRCKHYVELLVAQGADVHAQARGRFFQPKDEGGYFYFGELPLSLAACTNQPHIVNYL
TENPHKKADMRRQDSRGNTVLHALVAIADNTRENTKFVTKMYDLLLLKCARLFPDS
NLEAVLNNDGLSPLMMAAKTGKIGIFQHIIRREVTDEDTRHLSRKFKDWAYGPVYSSL
YDLSSLDTCGEEASVLEILVYNSKIENRHEMLAVEPINELLWDKWRKFGAVSFYINVVS
YLCAMVIFTLTAYYQPLEGTPPYPYRTTVDFLRLAGEIITLLTGVLFFLTSIKDCHEKCP

GVNSLFVDGSFQLLYFIYSVLVIVSAALYLAGLEAYLAVMVFALVLGWMNALYFTRGLK
LTGTYSIMIQKILFKDLFRFLLVYLLFMIGYASALVSLLTPCANMKVCDEGQSNCTTPTY
PSCRDSETFSTFLLDLFKLTIGMGDLEMLGSAKYPVVFIILLVTYIILTFVLLLNMLIALM
GETVGQVSKESKHIWKLQWATTILDIERSFPVFVRKAFRSGEMVTGKSSDGSPDRR
WCFRVDEVNWSHWNQNLGIINEDPGKNETYQYYGFSHTVGRLRRDRWSSVVPRVVE
LKNNSNPDEVVVPLAHVGNPSGPGHQQSHPPWRTDDAPA-----------------------------
----

>TRPV4_Canis_familiaris

MADPSEGPHTGPGEVAETPGDESGTPGSEAFPLSSLANLFEGEDGSPSPSPADTGRPA
GPGDGRPNLRMKFQGAFRKGVPNPIDLLESTLYESSVVPGPKKAPMDSLFDYGTYRH
HPSDNKRWRKKVIEKQPQSPKAPAPQPPPILKVFNRPILFDIVSRGSTTDLDGLLPFLL
THKKRLTDEEFREPSTGKTCLPKALLNLSNGRNDTIPVLLDIAERTGNMREFINSPFRD
IYYRGQTALHIAIERRCKHYVELLVAQGADVHAQARGRFFQPKDEGGYFYFGELPLSLA
ACTNQPHIVNYLTENPHKKADMRRQDSRGNTVLHALVAIADNTRENTKFVTKMYDL
LLLKCARLFPDSNLEAVLNSDGLSPLMMAAKTGKIGVFQHIIRREVTDEDTRHLSRKF
KDWAYGPVYSSLYDLSSLDTCGEEASVLEILVYNSKIENRHEMLAVEPINELLRDKWR
KFGAVSFYINVVSYLCAMVIFTLTAYYQPLEGTPPYPYRTTVDYLRLAGEIITLFTGVLF
FFTNIKDLFMKKCPGVNSLFIDGSFQLLYFIYSVLVIVSAALYLAGIEAYLAVMVFALVLG
WMNALYFTRGLKLTGTYSIMIQKILFKDLFRFLLVYLLFMIGYASALVSLLNPCANMKV
CSEDHTNCTVPTYPSCRDSETFSTFLLDLFKLTIGMGDLEMLSSTKYPVVFIILLVTYIIL
TFVLLLNMLIALMGETVGQVSKESKHIWKLQWATTILDIERSFPVFLRKAFRSGEMVT
VGKSSDGTPDRRWCFRVDEVNWSHWNQNLGIINEDPGKSENYQYYGFSHTVGRLRR
DRWSSVVPRVVELNKNSNPDEVVVPLDNMGNPSCDGHQQSYPPKWRTDDAPL-------

>TRPV4_Normascus_leucogenys

MADSSEGPRTGPGEVAELPGDESGTPGGEAFPLSSLANLFEGEDGSPSPLPADASRPAG
PGDGRPNLRMKFQGAFRKGVPNPIDLLESTLYESSVVPGPKKAPMDSLFDYGTYRHHP
SDNKRWRKKIIDGLLLCFYSPIHHPPLYLFPPIRNRLGEEMLTARTSEPLPSALTASSC
MSLLLEPSTGKTCLPKALLNLSNGRNDTIPVLLDIAERTGNMREFINSPFRDIYYRGQT
ALHIAIERRCKHYVELLVAQGADVHAQARGRFFQPKDEGGYFYFGELPLSLAACTNQP
HIVNYLTENPHKKADMRRQDSRGNTVLHALVAIADNTRENTKFVTKMYDLLLLKCA
RLFPDNNLEAVLNNDGLSPLMMAAKTGKIGNRHEMLAVEPINELLRDKWRKFGAVS
FYINVVSYLCAMVIFTLTAYYQPLEGTPPYPYHTTVDYLRLAGEVITLFTGVLFFFTNIK
DLFMKKCPGVNSLFIDGSFQLLYFIYSVLVIVSAALYMAGIEAYLAVMVFALVLGWMN
ALYFTRGLKLTGTYSIMIQKILFKDLFRFLLVYLLFMIGYASALVSLLNPCANMKVCNE
DQTNCTVPTYPSCRDSETFSTFLLDLFKLTIGMGDLEMLSSTKYPVVFIILLVTYIILTFV
LLLNMLIALMGETVGQVSKESKHIWKLQWATTILDIERSFPVFLRKAFRSGEMVTVGK
SSDGTPDRRWCFRVDEVNWSHWNQNLGIINEDPGKNETYQYYGFSHTVGRLRRDR
WSSVVPRVVELNKNSNPDEVVVPLDSMGNPRCDGHQQGYPPKWRTDDAPL----------

>TRPV4_Gorilla_gorilla_fragment

RKQPQSPKAPAPQPAPILKVFNRPILFDIVSRGSTADLDGLLPFLLTHKKRLTDEEFRE
PSTGKTCLPKALLNLSNGRNDTIPVLLDIAERTGNMREFINSPFRDIYYRGQTALHIAIE
RRCKHYVELLVAQGADVHAQARGRFFQPKDEGGYFYFGELPLSLAACTNQPHIVNYLT
ENPHKKADMRRQDSRGNTVLHALVAIADNTRENTKFVTKMYDLLLLKCARLFPDSN
LEAVLNNDGLSPLMMAAKTGKIGIFQHIIRREVTDEDTRHLSRKFKDWAYGPVYSSLY
DLSSLDTCGEEASVLEILVYNSKIENRHEMLAVEPINELLRDKWRKFGAVSFYINVVSY
LCAMVIFTLTAYYQPLEGTPPYPYRTTVDYLRLAGEVITLFTGVLFFFTNIKDLFMKKC
PGVNSLFIDGSFQLLYFIYSVLVIVSAALYLAGIEAYLAVMVFALVLGWMNALYFTRGLK
LTGTYSIMIQKILFKDLFRFLLVYLLFMIGYASALVSLLNPCANMKVCNEDQTNCTVPT
YPSCRDSGTFSTFLLDLFKLTIGMGDLEMLSSTKYPVVFIILLVTYIILTFVLLLNMLIAL
MGETVGQVSKESKHIWKLQWATTILDIERSFPVFLRKAFRSGEMVTVGKSSDGTPDR
RWCFRVDEVNWSHWNQNLGIINEDPGKNETYQYYGFSHTVGRLRRDRWSSVVPRVV
ELNKNSNPDEVVVPLDSMGNPRCDGHQQGYPPKWRTDDAPL---------------------------
----

>TRPV4_Cavia_porcellus

MADPGDSPCAGSGELAEPLGDDSGTAGGEAFPLSSLANLFEGEEGSSAPSPAEAGRPTA
QGDGRPNLRMKFQGAFRKGVPNPIDLLESTLYESSVVPGPKKAPMDSLFDYGTYRHH
PSDNRRWRRKVIEKQPQSPKAPAPQPPPILKVFNRPILFDIVSRGSTADLDGLLPFLLT
HKKRLTDEEFREPSTGKTCLPKALLNLSNGRNDTIPMLLDIAERTGNMREFINSPFRDI
YYRGQTALHIAIERRCKHYVELLVAQGADVHAQARGRFFQPKDEGGYFYFGELPLSLA
ACTNQPHIVNYLTENPHKKADMRRQDSRGNTVLHALVAIADNTRENTKFVTKMYDL
LLLKCARLFPDSNLEAVLNNDGLSPLMMAAKTGKIGIFQHIIRREVTDEDTRHLSRKFK
DWAYGPVYSSLYDLSSLDTCGEEVSVLEILVYNSKIENRHEMLAVEPINELLRDKWRK
FGAVSFYINVVSYLCAMIIFTLTAYYQPLEGTPPYPYRTTVDYLRLAGEVITLFTGVLFFF
TNIKDLFMKKCPGVNSLFIDGSFQLLYFIYSVLVIISAALYLAGIEAYLAVMVFALVLGW
MNALYFTRGLKLTGTYSIMIQKILFKDLFRFLLVYLLFMIGYASALVSLLNPCANMKVC
NEDGTNCTVPTYPSCRDSDTFSTFLLDLFKLTIGMGDLEMLSSIKYPVVFIILLVTYIILT
FVLLLNMLIALMGETVGQVSKESKHIWKLQWATTILDIERSFPVFLRKAFRSGEMVTV
GKNSDGTPDRRWCFRVDEVNWSHWNQNLGIINEDPGKSDTYQYYGFSHTVGRLRRD
RWSSVVPRVVELNKNSSPDEVVVPLDNLGNPSCDGHQRGHPPKWRTDDAPL----------
----

>TRPV4_Spermophilus_tridecemlineatus

MADPSDSPRAGPGDPAEPPGEESGTPGGEAFPLSSLANLFEGEDGSPSPSPADTARPA
GPGDGRPNLRMKFQGAFRKGVPNPIDLLESTLYEASVVPGPKKAPMDSLFDYGTYRH
HPSDNRRWRRKVMEKQPQSPKAPAPQPPPILKVFNRPILFDIVSRGSTADLDGLLPFL
LTHKKRLTDEEFREPSTGKTCLPKALLNLSNGRNDTIPVLLDIAERTGNMREFINSPFR
DIYYRGQTALHIAIERRCKHYVELLVAQGADVHAQARGRFFQPKDEGGYFYFGELPLSL
AACTNQPHIVNYLTENPHKKADMRRQDSRGNTVLHALVAIADNTRENTKFVTKMYD
LLLLKCARLFPDSNLEAVLNNDGLSPLMMAAKTGKIGIFQHIIRREVTDEDTRHLSRKF
KDWAYGPVYSSLYDLSSLDTCGEEVSVLEILVYNSKIENRHEMLAVEPINELLRDKWR
KFGAVSFYINVVSYLCAMVIFTLTAYYQPLEGTPPYPYRTTVDYLRLAGEVITLFTGVLF
FFTNIKDLFMKKCPGVNSLFIDGSFQLLYFIYSVLVIVSAALYLAGIEAYLAVMVFALVLG
WMNALYFTRGLKLTGTYSIMIQKILFKDLFRFLLVYLLFMIGYASALVSLLHPCANMKV

CDEDQTNCTVPTYPSCRDSETFSTFLLDLFKLTIGMGDLEMLSSTKYPVVFIILLVTYIIL
TFVLLLNMLIALMGETVGQVSKESKHIWKLQWATTILDIERSFPVFLRKAFRSGEMVT
VGKSSDGTPDRRWCFRVDEVNWSHWNQNLGIINEDPGKSETYQYYGFSHTVGRLRR
DRWSSVVPRVVELNKNSSPDEVAVPLDNLGNPSCDSHQQSYPPKWRTDDAPL--------

>TRPV4_Otolemur_garnettii

MADSSEGPRGGPAEVAEPPGDDGGTPGGEAFPLSSLANLFEGEDGSPSPPADVGRPPG
PGDGRPNLRMKFQGAFRKGVPNPIDLLESTLYESSVVPGPKKAPMDSLFDYGTYRHHP
SDNKRWRRKVIEKQPQSPKAPAPQPPPILKVFNRPILFDIVSRGSTADLDGLLPFLLTH
KKRLTDEEFREPSTGKTCLPKALLNLSNGRNDTIPVLLDIAERTGNMREFINSPFRDIY
YRGQTALHIAIERRCKHYVELLVAQGADVHAQARGRFFQPKDEGGYFYFGELPLSLAA
CTNQPHIVNYLTENPHKKADMRRQDSRGNTVLHALVAIADNTRENTKFVTKMYDLL
LLKCARLFPDSNLEAVLNNDGLSPLMMAAKTGKIGIFQHIIRREVTDEDTRHLSRKFK
DWAYGPVYSSLYDLSSLDTCGEEASVLEILVYNSKIENRHEMLAVEPINELLRDKWRK
FGAVSFYINVVSYLCAMVIFTLTAYYQPVEGTPPYPYRTTVDYLRLAGEIITLLTGVLFF
FTNIKDLFMKKCPGVNSLFIDGSFQLLYFIYSVLVIVSAALYLAGIEAYLAVMVFALVLG
WMNALYFTRGLKLTGTYSIMIQKILFKDLFRFLLVYLLFMIGYASALVSLLKPCANMKV
CNGDPTNCTVPTYPSCRDSDTFSTFLLDLFKLTIGIGDLEMLNSTKYPVVFIILLVTYIIL
TFVLLLNMLIALMGETVGQVSKESKHIWKLQWATTILDIERSFPVFLRKAFRSGEMVT
VGKSSDGTPDRRWCFRVDEVNWSHWNQNLGIINEDPGKSETYQYYGFSHTVGRLRR
DRWSSVVPRVVELNKNSSPDEVVVPLDNVGNPSCDGHQQSYPPKWRTDDAPL--------

>TRPV4_Sus_scrofa

MVEPSEGPRTGPGEVAEPPGDESGTSGGEAFPLSSLANLFEGEDGSPSPSLADPGRPTG
PGDGRPNLRMKFQGAFRKGVPNPIDLLESTLYESSVVPGPKKAPMDSLFDYGTYRHHP
SDNKRWRRKVIEKQPQSPKAPAPQPPPILKVFNRPILFDIVSRGSTTDLDGLLPFLLTH
KKRLTDEEFREPSTGKTCLPKALLNLSNGRNDTIPVLLDIAERTGNMREFINSPFRDIY
YRGQTALHIAIERRCKHYVELLVAQGADVHAQARGRFFQPKDEGGYFYFGELPLSLAA
CTNQPHIVNYLTENPHKKADMRRQDSRGNTVLHALVAIADNTRENTKFVTKMYDLL
LLKCARLFPDSNLEAVLNNDGLSPLMMAAKTGKIGIFQHIIRREVTDEDTRHLSRKFK
DWAYGPVYSSLYDLSSLDTCGEEASVLEILVYNSKIENRHEMLAVEPINELLRDKWRK
FGAVSFYINVVSYLCAMVIFTLTAYYQPLEGTPPYPYHTTVDYLRLAGEIITLFTGVLFF
FTNIKDLFMKKCPGVNSLFIDGSFQLLYFIYSVLVIVSAGLYLAGIEAYLAVMVFALVLG
WMNALYFTRGLKLTGTYSIMIQKILFKDLFRFLLVYLLFMIGYASALVSLLNPCANMKV
CSEDHTNCTVPTYPSCRDSETFSTFLLDLFKLTIGMGDLEMLSSTKYPVVFIILLVTYIIL
TFVLLLNMLIALMGETVGQVSKESKHIWKLQWATTILDIERSFPVFLRKAFRSGEMVT
VGKSSDGTPDRRWCFRVDEVNWSHWNQNLGIINEDPGKNESYQYYGFSHTVGRLRR
DRWSSVVPRVVELNKNSNPDEVVVPLDNMGKPSCDGHQQSYPPKWRTDDAPL-------

>TRPV4_Ailuropoda_melanoleuca

MADPSEGPHAGPGEVAESPGDESGTPGGEAFPLSSLANLFEGEDGSPSPSPADPGRPA
GPGDGRPNLRMKFQGAFRKGVPNPIDLLESTLYESSVVPGPKKAPMDSLFDYGTYRH
HPSDNKRWRKKVIEKQPQSPKAPAPQPPPILKVFNRPILFDIVSRGSTTDLDGLLPFLL

THKKRLTDEEFREPSTGKTCLPKALLNLSNGRNDTIPALLDIAERTGNMREFINSPFR
DIYYRGPLSVLHIAIERRCKHYVELLVAQGADVHAQARGRFFQPKDEGGYFYFGELPLS
LAACTNQPHIVNYLTENPHKKADMRRQDSRGNTVLHALVAIADNTRENTKFVTKMY
DLLLLKCARLFPDSNLEAVLNNDGLSPLMMAAKTGKIGVFQHIIRREVTDEDTRHLSR
KFKDWAYGPVYSSLYDLSSLDTCGEEASVLEILVYNSKIENRHEMLAVEPINELLRDK
WRKFGAVSFYINVVSYLCAMVIFTLTAYYQPLEGTPPYPYRTTVDYLRLAGEVITLFTG
VLFFFTNIKDLFMKKCPGVNSLFIDGSFQLLYFIYSVLVIVSAALYLAGIEAYLAVMVFAL
VLGWMNALYFTRGLKLTGTYSIMIQKILFKDLFRFLLVYLLFMIGYASALVSLLNPCAN
MKVCSEDHTNCTVPTYPSCRDSETFSTFLLDLFKLTIGMGDLEMLGSTKYPVVFIILLV
TYIILTFVLLLNMLIALMGETVGQVSKESKHIWKLQWATTILDIERSFPVFLRKAFRSG
EMVTVGKSSDGTPDRRWCFRVDEVNWSHWNQNLGIINEDPGKSENYQYYGFSHTVG
RLRRDRWSSVVPRVVELNKNSNQDEVVVPLDNMGNPSCDGHQQSYPPKWRTDDAP
L--

>TRPV4_Mus_musculus

MADPGDGPRAAPGEVAEPPGDESGTSGGEAFPLSSLANLFEGEEGSSSLSPVDASRPAG
PGDGRPNLRMKFQGAFRKGVPNPIDLLESTLYESSVVPGPKKAPMDSLFDYGTYRHHP
SDNKRWRRKVVEKQPQSPKAPAPQPPPILKVFNRPILFDIVSRGSTADLDGLLSFLLTH
KKRLTDEEFREPSTGKTCLPKALLNLSNGRNDTIPVLLDIAERTGNMREFINSPFRDIY
YRGQTSLHIAIERRCKHYVELLVAQGADVHAQARGRFFQPKDEGGYFYFGELPLSLAA
CTNQPHIVNYLTENPHKKADMRRQDSRGNTVLHALVAIADNTRENTKFVTKMYDLL
LLKCSRLFPDSNLETVLNNDGLSPLMMAAKTGKIGVFQHIIRREVTDEDTRHLSRKFK
DWAYGPVYSSLYDLSSLDTCGEEVSVLEILVYNSKIENRHEMLAVEPINELLRDKWRK
FGAVSFYINVVSYLCAMVIFTLTAYYQPLEGTPPYPYRTTVDYLRLAGEVITLFTGVLFF
FTSIKDLFTKKCPGVNSLFVDGSFQLLYFIYSVLVVVSAALYLAGIEAYLAVMVFALVLG
WMNALYFTRGLKLTGTYSIMIQKILFKDLFRFLLVYLLFMIGYASALVTLLNPCTNMK
VCDEDQSNCTVPTYPACRDSETFSAFLLDLFKLTIGMGDLEMLSSAKYPVVFILLLVTYI
ILTFVLLLNMLIALMGETVGQVSKESKHIWKLQWATTILDIERSFPVFLRKAFRSGEMV
TVGKSSDGTPDRRWCFRVDEVNWSHWNQNLGIINEDPGKSEIYQYYGFSHTVGRLRR
DRWSSVVPRVVELNKNSSADEVVVPLDNLGNPNCDGHQQGYAPKWRTDDAPL-------

>TRPV4_Latimeria_chalumnae

MGDVEEQNKLNSSGDANDTRTEDGSQQNDAFPLSSLANLFENEEGATATDTAKAPQ
TAGDGKQNLRMKFHGAFKKGMPNPMDLLESTIYESPVVPAPKKAPMDSLFDYGTYH
QYPTENKRRRKRIIHDKQSPNQKTQAPNPPPILKVFNRPILFDIVSRGTAAELDGLLAF
LVTHKKRLADEEFREPSTGKTCLPKALLNLTNGKNDTIPILVEIAEKTGNLREFINSPF
RDVYYRGQTALHIAIERRCKQYVELLVEKGADVHAQARGRFFQPRDEGGYFYFGELPL
SLAACTNQPDIVHYLIENAHKKADLRRQDSRGNTVLHALVAIADNTRENTKFVTKMY
DMLLIKCAKLFPECNLEAILNNDGMSPLMMAAKLGKIGIFKYIIRREVMDENARHLSR
KFKDWAYGPVYSSLYDLSSLDTCGMEVSVLEILVYNSRIENRHEMLAVEPINELLRDK
WRKFGAVSFYISVVSYLVAMVIFTLIAYYRPVEGRPPYPYKTTIDYLRLAGEVITLLTGIF
FFFTNIKDLFLKKCPGVNSLLIDGSFQLLYFIYSVLVLVTAALYLTGIEAYVAVMVFALVL
GWMNTLYFTRGLKLTGTYSIMLQKILFKDLFRFLLVYVLFMIGYASALVSLLNPCLTTE
TCTANNASCTAPEYPYCRDSNTFGKFIMDLFKLTIGMGDLEMVNSAKYPGVFIILLVTY
IILTFVLLLNMLIALMGETVGQVSKESKQIWKLQWATTILDIERSFPVFLRRVFRSGEM
VTVGKNLDGTPDRRWCFRVDEVNWSHWNQNLGIINEDPGKNETYQYYGFSHTMGR

LRRDRWSTVVPRVVELNKTPRADEVVVSLDPMGMSNVHDTKRGDPHNWKKDETQI-

>TRPV4_Oreochromis_niloticus

MNEGRSAIFKRRHLALPKANAISSEPSISVDLGDSEAAQPEGDGAFPLSEFSHLFESQD
GSPATQDSSQESILEPAQPGHPADSRQYLRMKFHGAFKKGISNPMDLLESTIYESNVVP
APKKAPMDSLFDYGTYGNSSNQKKRRKKLPKGKTEASCDESQSSDPPKVVKVFNRSLL
FDCVSRGDPGELEGLLEYLQSNNKRLTDEEFREPYTGKTCLPKALMNLYGRQNNTIPV
LVDIAEKNGSLREFINTPFRDVYYRGQTALHIAIERRCKQYVKLLVEKGADVHAQARG
RFFQPKDEGGYFYFGELPLSLAACTNQPDIVHYLTENPHKKADVRRQDSRGNTVLHAL
VHIADNTKDNTRFLTKMYDLLLIKTAKLYPDCNLETVPNNDGMSPLMMAARLGKIGI
FQHIIRREIKDEEVRHLSRKFKDWAYGPVYSSLYDLSSLDTCGKESSVLEILVYTSHNEN
RHEMLAVEPINELLRAKWNRFAAVTFYISVFSYLITMIIFTLVAYYQPTHGKPPYPHTT
SSDYWRLAGEIVTLASGIFFFLTNIKDLFLKKCPGVKSLFIDGSFQLLYFIYSVLIIVTAAL
YLSGIEAYVSVMVFALALGWMNTLYFTRGLKLTGTYSIMIQKILFKDLFRFLLVYVLFM
IGFASALVSLLTVCPPPGTVCNGSCPTYPACRDNNTFSIFLLDLFKLTIGMGDLDMIYSA
QNPVVFLILLVTYIILTFVLLLNMLIALMGETVGQVSKESKKIWKLQWATTILDIERSFP
VCLRKSFRVGEMVTVGKNYDGTPDRRWCFRVDEVNWCHWNQNLAIINEDPGKSETI
QANGLQQGVRALRRDRWSTVVPRAVELSKGSQSHDLAVEMEPLSPRH----------------
----

>TRPV4_Meleagris_gallopavo

MADPEDPRDAGDALGDDSFPLSSLANLFEVEDTSSPAEPSRGPPGAGDGKQNLRMKF
HGAFRKGPPKPMELLESTIYESSVVPAPKKAPMDSLFDYGTYRQHPSENKRWRRRVV
EKPVAGTKGPAPSPPPVLKVFNRPILFDIVSRGSPDGLEGLLSFLLTHKKRLTDEEFREP
STGKTCLPKALLNLSAGRNDTIPILLDIAEKTGNMREFINSPFRDVYYRGQTALHIAIER
RCKHYVELLVEKGADVHAQARGRFFQPKDEGGYFYFGELPLSLAACTNQPHIVHYLTE
NGHKQADLRRQDSRGNTVLHALVAIADNTRENTKFVTKMYDLLLIKCAKLFPDTNLE
ALLNNDGLSPLMMAAKTGKIGIFQHIIRREIADEDVRHLSRKFKDWAYGPVYSSLYDLS
SLDTCGEEVSVLEILVYNSKIENRHEMLAVEPINELLRDKWRKFGAVSFYISVVSYLCA
MIIFTLIAYYRPMEGPPPYPYTTTIDYLRLAGEIITLLTGILFFFSNIKDLFMKKCPGVNS
FFIDGSFQLLYFIYSVLVIITAGLYLGGVEAYLAVMVFALVLGWMNALYFTRGLKLTGT
YSIMIQKILFKDLFRFLLVYLLFMIGYASALVSLLNPCPSSESCSEEHSNCTLPTYPSCRD
SQTFSTFLLDLFKLTIGMGDLEMLESAKYPGVFIILLVTYIILTFVLLLNMLIALMGETVG
QVSKESKHIWKLQWATTILDIERSFPLFLRRAFRSGEMVTVGKGTDGTPDRRWCFRV
DEVNWSHWNQNLGIISEDPGKSDTYQYYGFSHTVGRLRRDRWSTVVPRVVELNKSCP
TEDVVVPLGTMGTAEARERRHGQNSSSLL---

>TRPV4_Gallus_gallus

MADPEDPRDAGDVLGDDSFPLSSLANLFEVEDTPSPAEPSRGPPGAVDGKQNLRMKF
HGAFRKGPPKPMELLESTIYESSVVPAPKKAPMDSLFDYGTYRQHPSENKRWRRRVV
EKPVAGTKGPAPNPPPILKVFNRPILFDIVSRGSPDGLEGLLSFLLTHKKRLTDEEFREP
STGKTCLPKALLNLSAGRNDTIPILLDIAEKTGNMREFINSPFRDVYYRGQTALHIAIER
RCKHYVELLVEKGADVHAQARGRFFQPKDEGGYFYFGELPLSLAACTNQPHIVHYLTE
NGHKQADLRRQDSRGNTVLHALVAIADNTRENTKFVTKMYDLLLIKCAKLFPDTNLE

ALLNNDGLSPLMMAAKTGKIGIFQHIIRREIADEDVRHLSRKFKDWAYGPVYSSLYDLS
SLDTCGEEVSVLEILVYNSKIENRHEMLAVEPINELLRDKWRKFGAVSFYISVVSYLCA
MIIFTLIAYYRPMEGPPPYPYTTTIDYLRLAGEIITLLTGILFFFSNIKDLFMKKCPGVNS
FFIDGSFQLLYFIYSVLVIVTAGLYLGGVEAYLAVMVFALVLGWMNALYFTRGLKLTGT
YSIMIQKILFKDLFRFLLVYLLFMIGYASALVSLLNPCPSSESCSEDHSNCTLPTYPSCRD
SQTFSTFLLDLFKLTIGMGDLEMLESAKYPGVFIILLVTYIILTFVLLLNMLIALMGETVG
QVSKESKHIWKLQWATTILDIERSFPLFLRRAFRSGEMVTVGKGTDGTPDRRWCFRV
DEVNWSHWNQNLGIISEDPGKSDTYQYYGFSHTVGRLRRDRWSTVVPRVVELNKSCP
TEDVVVPLGTMGTAEARERRHGQTPSSPL--

>TRPV4_Oryzias_latipes_fragment

RADTLGSAGSAASEDCKEGDAAQAEGEAAFPLSELSQLFESEDGSQSAQDTSQESALEL
VQPGNPADSRQNLRTKFQGAFRKGISHPMDLFEATIYESNVVPAPKKAPMDSLFDYGT
YGNSSNQKKRRKKLPRGKTEASCDIVPNPDPPKVMKIFNRILLFDCVSRGDPEDLEGLL
EYLQVHEKRLTDEEFREPSTGKTCLPKALLNLYGGRNNTIPLLVDIAEKTGNLREFINT
PFRDVYYRGQTALHIAIERRCKHYVELLMEKGADVHAQARGRFFEPKDEGGYFYFGEL
PLSLAACTNQPNIVNYLTENPHKKADLRRQDSRGNTALHALVHIADNTKDNTRFLTK
MYDLLLIKCTKLYPECNLEKMANNDGLTPLMMAAKLGKIGVFQHIIRREIKDEEVRHL
SRKFKDWAYGPVYSSLYDLSSLETCGEEPSVLEILVYNSRNENCHEMLAVEPINELLRA
KWQKFAAVTFYISVVSYLITMIIFTLVAYYHPTEGKPPFPYTTSTDYLRMVGEIFTLASG
IFFFLTNIKDLFLKKRPGVKSLVMDGSFQLLYFIYSILIIITAALYLSGIKAYVSVMVFALV
LGWMNTLYFTRGLKLTGTYSIMIQKILLKDIFRFLLVYLLFMIGYASALVSLLTVCPTSG
PECEGGCPTYPKCREPGTFSTFLLDLFKLTIGMGDLEMINSAQYPEVFLILLVTYIILTFV
LLLNMLIALMGETVGQVSKESKKIWKLQWATTILDIEHSFPVCLRRSFRVGEMVTVGK
NLDGTPDRRWCFRVDEVNWCHWNQNLAIINEDPGRSDTSQTNGLRQSVKGLRRDR
WTTVVPRVMELSKSPQPHDLVVEMEPLTTRN--

>TRPV4_Tetraodon_nigroviridis_fragment

LSKADTLGSDPNGQTSQAEGDAAFPLSDLSQLFESEDASPSAQDTGQASVLGPVPPGQ
PADGRQNLRMKFQGAFKKGISNPMDLLESTIYESNVVPGPKKAPMDSLFDYGTYRNP
SNQKRRRKKLPRGLVKAEASCDDGQADDPPKVLKVFNRSLLFDCVSRADAEALEGLL
EYLQSREKRLTDEEFREPSTGKTCLPKALLNLYSGQNVTIPLLVDIAERTGNLRELLNT
PFRDVYYRGQTALHIAIERRCKQYVELLVEKGADVHAQARGRFFQPRDEGGYFYFGEL
PLSLAACTNQPDIVHYLTENPHKKADLRRQDSRGNTVLHALVHIADNTKDNTRFLTK
MYDLLLIKSAKLYPDCNLETVLNNDGMSPLMMAAKLGKIGVFQHIIRREIKDEEVRHL
SRKFKDWVYGPVYSSLYDLSSLDTCGEEPSVLEILVYNSRTENHHEMLAVEPINELLRA
KWQKFAAVSFYVSVVSYLTTMIIFTLVAYHHPVQGKPPHPYTTSSDYLRMAGEVITLA
SGIFFFLTNIKDVFLKKCPGVKSLVIDGSFQLLYFIYSVLIVVSAALYLSGIEAYMSVMVF
ALVLGWMNALYFTRGLKLTGTYSIMIQKILFKDLFRFLLVYVLFMIGYASALVSLLTVC
PPPGTECEGGCPTYPKCRDPDTFSTFLLDLFKLTIGMGDMIWDSARYPAVFLILLITYII
LTFVLLLNMLIALMGETVGQVSKQSKKVWKLQWATTILDIERSFPVCLRKSFRAGEMV
TVGRNSDGTPDRRWCFRVDEVNWCHWNQNLAIINEDPGKNETCQANGLQQGVRAL
RRDRWSTVVPRALELSKGPHHRDLVIEMEPLTPRH---

>TRPV4_Danio_rerio

QGFSASTLLKRYRLAMTESLSVSSPPDNSAQDSSEAADGDPNFPMSSMAALLENDDVS
QPTHELPRPGQQNDQKQNMRIRFPGPFKKGVPNPMDLLESDYTEYPKQAPMDSMFD
YGTCRQINNNKKGRRKKLPRGKAEIGMSCDEGSPEPPVLKVFNRWMLFEAVSRADPR
ALDGLLQYLQSHEKRLTDEEFKELSTGKTCLPKALLNLHNGQNDTIPILVDIAEQTGNL
REFINTPFRDVYYRGQMALHIAIERRCKQYVELLVEKGADVHAQARGRFFQPRDEGGY
FYFGELPLSLAACTNQPDMVHYLTENGHKKADLRRQDSRGNTVLHALVHIADNTRD
NTRFVTKMFDLLLIKCAKLYPDCNLENILNNDGMSPLMMAAKLGKIGVFQHIIRREIK
DEEARHLSRKFKDWAYGPVYSNLYDLSSLDTCGEEVSVLEILVYNSKIENRHEMLAVE
PINELLRAKWQKFAAVTFYISVFSYLVTMIIFTLVAYYRPSVGKPPYAYDTTEDKVRLA
GEIITVGSGLFFFVTNIKDLFLKKCPGVNSIFVDGSFQLLYFIYSVLVLVSAALYLSGIEAY
VSVMVFALTLGWMNTLYFTRGLKLTGTYSIMIQKILIKDLFRFLLVYVLFMIGYASALV
SLLTICPDKDTCKENCPTYPECRDTNTFSEFLLDLFKLTIGIGDLDNMLKGAQYPAVFLI
LLVTYIILTFVLLLNMLIALMGETVGQVSKESKKIWKLQWATTILDIERSFPVCLRRSFR
VGEMVTVGKGLDGKPDKRWCFRVDEVKWSHWNQNLGIINEDPGQKDLSEHTQGGR
GLRRDRWSTVVPRVVELNRGSRDHTVEMEPLTGRHRLKSES---------------------------
----

>TRPV4_Takifugu_rubripes

TLSSDCSGPAAHASANNGRTAQAEGDAAFPLSELSQLFESEDASPSAQGASQEPAPPG
QPPDSRQNLRMKFQGAFKKGISNPMDLLESTIYESNVVPGPKKAPMDSLFDYGTYRN
TSNQKRRRKKLPRGKTETSCDEGLAADPPKVLKVFNRSLLFDCVSRADAEALEGLLEY
LQSQEKRLTDEDFREPSTGKTCLPKALLNLYGGQNVTIPLLADIAEKTGNLREFINTPF
RDVYYRGQTALHIAIERRCKPYVELLVEKGADVHAQARGRFFQPRDEGGYFYFGELPL
SLAACTNQPDIVHYLTENPHKKADLRRQDSRGNTVLHALVHIADNTKDNTRFLTKMY
DLLLIKSAKLYPDCSLETVLNNDGMSPLMMAAKLGKIGVFQHIIRREIKDEEARHLSRK
FKDWAYGPVYSSLYDLSSLDTCGEEPSVLEILVYNSRNENRHEMLAVEPINELLRAKW
QKFAAVTFYISVVSYLITMIIFTLVAYYHPTQGKPPYPYTTSSDYLRMAGEVITLASGIFF
FLTNIKDVFLKKCPGVKSLFIDGSFQLLYFVYSVLIVISAALYLSGIEAYVSVMVFALVLG
WMNTLYFTRGLKLTGTYSIMIQKILFKDLFRFLLVYVLFMIGYASALVSLLTVCPPPGT
ECDGGCPTYPKCRDPDTFSTFLLDLFKLTIGMGELDMIHSAQYPAVFLILLVTYIILTFV
LLLNMLIALMGETVGQVSKQSKKIWKLQWATTILDIERSFPVCLRKSFRAGEMVTVGK
NCDGTPDRRWCFRVDEVNWCHWNQNLAIINEDPGKNETCQATGLQQGVRALRRDR
WSTVVPRALELSKGPHNRDLAIEMEPLTPRH--

>TRPV4_Xenopus_tropicalis

MADPSHLLKHNASVDIDDSQGDDGSNHNDSFPLSSLANLFENEESSAPNEGVRSPQVP
GDNKQNLRIRFQGPFRKGISNPMDLLESTIYESSAPKKAPMDSLFGYETYHHHPTENR
RKRKKILLEKENLNSQAPSPDPPPVIKMFNRHMLFDIVSRGSTAELEGFLPFLLAQKKR
LTDEEFREASTGKTCLTKALMNLNGGKNDTIPMLIDIAEKTGNLREFINSPFRDVYYR
GQTALHIAIERRCKHYVELLVEKGADVHAQARGRFFQPKDEGGYFYFGELPLSLAACT
NQPDIVHYLTENAHKKADIRRQDSRGNTVLHALVAIADNTRENTKFVTKVYDLLVIKC
VKLYPDSSLEAIFNNDSMSPLMMAAKLGKIGIFQHIIRLEIKDEEARHLSRKFRDWAYG
PVYSSLYDLSMLDTCGEEVSVLEILVYNSKVENRHEMLAVEPINELLRDKWQKFGAVS
FYISVVSYLIAMIIFTLIAYYRPMDGTPPYPYRTTMDYMRLAGEIVTLLTGVVFFITNIKD

LFMKKCPGVNSLFIDGSFQLLYFIYSVLVIITAVLYLVGIESYLAVMVFALVLGWMNALY
FTRGLKLTGTYSIMLQKILFKDLFRFLLVYLLFMIGYASALVSLLNPCTSQESCIETSSNC
TVPEYPSCRDSSTFSKFLLDLFKLTIGMGDLEMINSAKYPAVFIILLVTYIILTFVLLLNM
LIALMGETVGQVSKESKQIWKLQWATTILDIERSFPVCMRKAFRSGEMVTVGKNLDG
TPDRRWCFRVDEVNWSHWNQNLGIINEDPGRNDGYQYYGFSQTVGRLRRDRWSVV
VPRVVELNKAPQHSDDVVVPLGNIPQVQTYSQRQENAQNWKKDETHI-----------------
----

>TRPV4_Elaphe_quadrivirgata

MANLEDAAHASPSESTESPSEELSPQNDSFPLSSLANLFENEDGAPAAEAARTPPGAG
DGKQNLRMKFHGAFRKGVPNPMDLLESTIYESSVVPGPKKAPMDSLFDYGTYRHHPS
DNKRRRKKALEKKPPSTKGPAPHPPPILKVFNRPILFDIVSRGSTADLDGLLPFLLTHK
KRLTDEEFREPSTGKTCLPKALLNLNNGKNDTIPFLLDIAEKTGSTREFINSPFRDVYY
RGQTALHIAIERRCKHYVELLVEKGADVHAQARGRFFQPKDEGGYFYFGELPLSLAAC
TNQPHIVQYLTENAHKQADLRRQDSRGNTVLHALVAIADNTRENTKFVTKMYDLLLI
KCAKLFPDTNLEALLNNDGLSPLMMAAKTGKIGMFQHIIRREVKDEEARHLSRKFRD
WAYGPVYSSLYDLSSLDTCGEEVSVLEILVYNSKIENRHEMLAVEPINELLRDKWRKF
GAVSFYISVVSYLCAMVIFTLVAYYRPLEGTPPYPYTTTPDYLCLAGEIVTLFTGVLFFFT
NIKDLFMKKCPGVNSFFIDGSFQLLYFIYSVLVLVAAALYLAGIEAYLAVMVFALVLGW
MNALYFTRGLKLTGTYSIMIQKILFKDLFRFLLVYVLFMIGYASALVSLLNPCPSAESCR
GDHSNCTAPAYPSCRDSKTFSTFLLDLFKLTIGMGDLEMIENAKYPGVFVILLVTYIILT
FVLLLNMLIALMGETVGQVSKESKKIWKLQWATTILDIERSFPVFVRRAFRSGEMVTV
GKSLDGAPDRRWCFRVDEVNWSHWNQNLGIISEDPGKNDTYQYYGFSHTVGRLRRD
RWSTVVPRVVELNKNSQPDEVVVPLDSMCSAGANAHKPSYPHSWRKEDAQI----------
----

>TRPV4_Takydromus_tachydromoides

MANLEDAAHASPSESTESPSEELSPQNDSFPLSSLANLFENEDGAPAAEAARTPPGAG
DGKQNLRMKFHGAFRKGVPNPMDLLESTIYESSVVSGPKKAPMDSLFDYGTYRHHPS
DNKRRRKKALEKKPPSTKGPAPHPPPILKVFNRPILFDIVSRGSTADLDGLLPFLLTHK
KRLTDEEFREPSTGKTCLPKALLNLNNGKNDTIPFLLDIAEKTGSTREFINSPFRDVYY
RGQTALHIAIERRCKHYVELLVEKGADVHAQARGRFFQPKDEGGYFYFGELPLSLAAC
TNQPHIVHYLTENAHKQADLRRQDSRGNTVLHALVAIADNTRENTKFVTKMYDLLLI
KCAKLFPDTNLEALLNNDGLSPLMMAAKTGKIGAFQHIIRREIKDEDARHLSRKFKD
WAYGPVYSSLYDLSSLDTCGEEDSVLEILVYNSKMENRHEMLAVEPINELLRDKWRKF
GAVSFYISVVSYLCAMIIFTMVAYYRPLEGTPPYPYTTTTDYLRLAGEIVTLFTGVLFFF
TNIKDLFMKKCPGVNSFFIDGSFQLLYFIYSVLVLVAAALYLTGIEAYLAVMVFALVLG
WMNALYFTRGLKLTGTYSIMIQKILFEDLFRFLLVYVLFMIGYASALVSLLNPCPSSEAC
SEERSNCTAPAYPSCRDSKTFSNFLLDLFKLTIGMGDLEMIESAKYPGVFVILLVTYIILT
FVLLLNMLIALMGETVGQVSKESKQIWKLQWATTILDIERFFPVFVRKAFRSGEMVTV
GKSLDGTPDRRWCFRVDEVNWFHWNQNLGIINEDPGKNDTYQYYGFSHTVGRLRRD
RWSTVVPRVVELNKNLQPDEVVVPLDSMRSPAANEHKPSYPQSWRKEDSHI----------
----

>TRPV4_Oreochromis_mossambicus

MNEGRSAIFKRRHLALPKGNAISSEPSISVDLGDSEAAQPEGDGAFPLSEFSHLFESQD
GPPATQDSSQESILEPAQPGHPADSRQYLRMKFHGAFKKGISNPMDLLESTIYESNVVP
APKKAPMDSLFDYGTYGNSSNQKKRRKKLPKGKTEASCDESQSSDPPKVVKVFNRSLL
FDCVSRGDPGELEGLLEYLQSNNKRLTDEEFREPYTGKTCLPKALMNLYGRQNNTIPV
LVDIAEKNGSLREFINTPFRDVYYRGQTALHIAIERRCKQYVKLLVEKGADVHAQARG
RFFQPKDEGGYFYFGELPLSLAACTNQPDIVHYLTENPHKKADVRRQDSRGNTVLHAL
VHIADNTKDNTRFLTKMYDLLLIKTAKLYPDCNLETVPNNDGMSPLMMAARLGKIGI
FQHIIRREIKDEEVRHLSRKFKDWAYGPVYSSLYDLSSLDTCGKESSVLEILVYTSHNEN
RHEMLAVEPINELLRAKWNRFAAVTFYISVFSYLITMIIFTLVAYYQPTHGKPPYPHTT
SSDYWRLAGEIVTLASGIFFFLTNIKDLFLKKCPGVKSLFIDGSFQLLYFIYSVLIIVTAAL
YLSGIEAYVSVMVFALALGWMNTLYFTRGLKLTGTYSIMIQKILFKDLFRFLLVYVLFM
IGFASALVSLLTVCPPPGTVCNGSCPTYPACRDNNTFSIFLLDLFKLTIGMGDLDMIYSA
QNPVVFLILLVTYIILTFVLLLNMLIALMGETVGQVSKESKKIWKLQWATTILDIERSFP
VCLRKSFRVGEMVTVGKNYDGTPDRRWCFRVDEVNWCHWNQNLAIINEDPGKSETI
QANGLQQGVRALRRDRWSTVVPRAVELSKGSQSHDLAVEMEPLSPRH----------------
----

>TRPV4_Gasterosteus_aculeatus

MNEGRSALFRRCHLALSKADTVGSVPGRNAASVDSGDGAAPQPDGDAALGLSELSHL
FENDDGSPSTQDTSGGSAPELVQPGQPAEGRQNLRMKFQGAFRKGISTHMDLLESTIY
ESNVVQGPKKAPMDSLFDYGTCRNTSNQKRRRKKLPRGKTEASCDDDGQSSDPPKV
MKVFNRSLLFDAVSRADPEALEGLLEYLQSHEKRLTDEEFKELSTGKTCLPKALLNLY
GGQNVTIPLLVDVAEKTGNLREFINTPFRDVYYRGQTALHIAIERRCKQYVELMVEHG
ADVHAQARGRFFQPRDEGGYFYFGELPLSLAACTNQPNIVHYLTENPHKKADLRRQD
SRGNTVLHALVHIADNTKDNTRFLTKMYDLLLVKSAKLYPDCNLETVLNNDGMSPLM
MAAKLGKIGVFQHIIRREIKDEEVRQLSRKFKDWAYGPVYSSLYDLSSLDTCGEEPSVL
EILVYNSRNENRHEMLAVEPINELLRAKWQKFAAVTFYISVVSYLITMIIFTLVAYYHP
TQGTPPYPYTTSSDYLRMAGEILTLASGIFFFLTNIKDLFLKKCPGVKSLFMDGSFQLLY
FIYSVLIVVTAALYLSGIKAYVSVMVFALVLGWMNTLYFTRGLKLTGTYSIMIQKILFKD
LFRFLLVYVLFMIGYASALVSLLTMCPPPGTECEGGCPTYPKCRDPDTFSAFLLDLFKL
TIGMGELDMIHGAQYPAVFLILLVTYIILTFVLLLNMLIALMGETVGQVSKESKKIWKL
QWATTILDIERSFPVCLRKSFRAGEMVTVGKSWDGTPDRRWCFRVDEVNWCHWNQ
NLAIINEDPGKNEVCQANGLQQGVRALRRAVDRWSTVVPRAVELSKGPRPRDLSIEM
EPL--
